# Trends in public perceptions of patient safety during the COVID-19 pandemic: Findings from a repeated cross-sectional survey in Germany, 2019–2023

**DOI:** 10.1371/journal.pone.0329761

**Published:** 2025-08-05

**Authors:** Olga Amberger, Dorothea Lemke, Hardy Müller, David Schwappach, Max Geraedts, Beate S. Müller

**Affiliations:** 1 Institute of General Practice, Goethe University Frankfurt, Frankfurt am Main, Germany; 2 Gesundheitsamt Frankfurt am Main, Frankfurt am Main, Germany; 3 German Society for Patient Safety, Reutlingen, Germany; 4 Institute of Social and Preventive Medicine (ISPM), University Bern, Bern, Switzerland; 5 Institute of Health Services Research and Clinical Epidemiology, University of Marburg, Marburg, Germany; 6 Institute of General Practice, University of Cologne, Cologne, Germany; Charite Universitatsmedizin Berlin, GERMANY

## Abstract

In recent years, public perceptions of patient safety have evolved significantly, driven by media coverage, healthcare reforms, and greater awareness during the coronavirus disease 2019 (COVID-19) pandemic. The present study aimed to examine trends in public perceptions of patient safety, knowledge and self-efficacy in Germany during this pandemic between 2019 and 2023. A repeated cross-sectional study was conducted, using data from TK Monitor of Patient Safety. TK Monitor of Patient Safety in a nationwide survey assessing public perceptions of safety in medical treatment and diagnosis. Self-reported data were collected annually from a randomly selected sample of 1,000 different adults aged 18 and older residing in Germany. Statistical analyses included descriptive statistics, chi-square tests, and linear regressions for trend analyses. Our results revealed high perceived patient safety risk during the years of the study. Up to one third of respondents considered it very likely or somewhat likely that patients would be harmed when receiving medical treatment in hospital or ambulatory care, with lower perceived levels of risk before the COVID-19 pandemic. Regarding perceived prevalence of preventable adverse events, over half of respondents considered it very likely or somewhat likely that an illness of theirs would be diagnosed incorrectly, or that they would contract a nosocomial infection, at some stage in their lives. The majority of respondents considered themselves overall well informed about patient safety and reported higher levels of self-efficacy with regard to error prevention before and after the pandemic than during it. Given the facts that patient safety remains an important issue and that the German public perceives the level of patient safety risk but also of patient safety knowledge, and self-efficacy as high, actively involving patients in safety initiatives is essential for shaping positive public perception.

## Introduction

Patient safety plays a crucial role in maintaining high-quality care and is a key priority for healthcare systems globally [1]. The main goal of patient safety is to minimize risks, errors, and harm that may arise during healthcare delivery. Achieving this requires a collaborative, multidisciplinary effort involving policymakers, healthcare professionals, and patients. Numerous concerns regarding safety in both hospital and outpatient settings, such as medical care or service and medication errors, have been increasingly highlighted over the past 15 years [1–3]. It is estimated that one in ten patients experiences harm during healthcare, with over three million deaths occurring each year due to unsafe care worldwide [4]. This harm also impacts global economic growth, reducing it by 0.7% annually. In Germany, the exact frequency of preventable adverse events remains a subject of debate due to different methods of collecting data [5]. Preventable adverse events are caused by both active errors and latent system failures embedded within the healthcare system.

The extent of patient harm in healthcare was first brought to global and media attention in the 1990s. A landmark report by the Institute of Medicine ‘To Err is Human’, published in 1999, revealed that up to 98,000 deaths annually in the USA were due to preventable medical errors [6]. This report had a profound influence on public perception and healthcare policy. In 2005, a survey by the European Union revealed that 72% of Germans and 78% of EU citizens viewed medical errors as a significant issue [7]. Moreover, 29% of Germans surveyed expressed concern that they might personally experience a medical error. In a representative survey of over-40s in Germany, 14.2% of participants stated that they had experienced a patient safety incident in outpatient care in the last 12 months [6]. Two decades after the report, major advancements in healthcare technology, patient safety initiatives, and the development of public reporting systems play a major role in shifting public perception [8]. Moreover, the rise of digital health tools and patient advocacy groups has empowered patients to take an active role in their own care, demanding clearer communication and measures to prevent errors [9]. The World Health Organization (WHO) is encouraging patient engagement as one of the most powerful tools to improve patient safety [10]. Patients’ perception of self-efficacy can significantly influence their involvement in preventing errors [11]. Healthcare systems have thus placed more emphasis on patient-centered care and the adoption of safety protocols, improving public confidence to some extent. However, barriers, such as limited financial resources and lack of knowledge, continue to influence public trust in patient safety efforts [12]. In Germany, these trends have mirrored broader global concerns, with increased expectations for transparency and efforts to involve patients in safety measures [13]. The COVID-19 pandemic further spotlighted patient safety, especially in areas like infection control, and the pressures on overwhelmed healthcare systems [14,15]. It exposed both the strengths and weaknesses of safety protocols, raising public awareness of safety challenges in healthcare environments. Germany faced three waves of COVID-19 infection. The first wave from March to May 2020 was characterized by shortages of protective equipment and the need for safety measures. The government imposed social distancing rules and lockdowns to prevent the spread of the disease. The second wave from September 2020 to May 2021 resulted in a large number of hospitalizations and deaths. Lockdowns and restrictions were reintroduced and the process of a mass vaccination was rolled out. The third wave from summer 2021 to early 2022 brought a renewed spike in COVID-19 cases with new variants such as Delta and Omicron, despite vaccination efforts. In the post-pandemic era, healthcare systems addressed backlogs of procedures, revised safety protocols, and prioritized staff mental health and resilience. Telemedicine has since become a significant part of healthcare delivery [16]. Overall, the pandemic highlighted gaps in preparedness and has reinforced the critical need for adaptable, resilient healthcare systems [17].

Understanding this progression helps people appreciate how public trust in healthcare systems has changed. The main objective of the study is hence to explore nationwide patterns and trends in perceived patient safety, knowledge and self-efficacy from a population perspective before, during, and after the COVID-19 pandemic from 2019 to 2023 in Germany.

## Methods

### Data

This study draws on the Techniker Krankenkasse (TK) Monitor of Patient Safety, an annual large-scale national survey study designed to analyze the German general public’s perception of patient safety trends. Detailed information about the study design can be found in other sources [14]. It collects population-related data on perceptions, experiences, and knowledge related to patient safety from 1,000 randomly selected participants through computer-assisted telephone interviews (CATI). The survey is also updated annually to include questions on specific issues. The initial survey was conducted between October 29 and November 15, 2019, with later surveys August 3–30, 2020, June 6–21, 2021, April 25 to May 6, 2022, and June 1–23, 2023. Selected findings from these have been published in various reports [14,18,19]. The Strengthening the Reporting of Observational Studies in Epidemiology (STROBE) checklist served as the reporting guideline for this study (S1 Appendix).

### Participants

Self-reported data were collected from nationally representative samples of 1,000 different adults in each survey wave from 2019 to 2023. The inclusion criteria required participants to be at least 18 years old, and to live in a private household (70.37 million adults living in Germany in 2023). The exclusion criterion was insufficient German language skills. The sample’s representativeness was ensured through random selections from the Association of German Market and Social Research Institutes (Arbeitskreis Deutscher Marktforscher, ADM) samples and comparisons with data from the German Federal Statistical Office [20]. The ADM sampling system is based on the Gabler-Häder method [21] and takes a multi-level stratified random sampling approach with a dual frame design of 70% landline and 30% mobile phone numbers. In Germany, around 83% of households have a landline telephone connection and 82.2% of the population use cell phones. For selection of household respondents, we applied the last-birthday method, which identifies the household member with the most recent birthday. In the ADM sampling system, numbers from the German landline-based telephone network are generated as blocks of numbers with a range of 10 (*Festnetzdatei*). Numbers from the German cellular telephone network are generated as number blocks with a range of 10000 (*Mobilfunkdatei*). The samples accurately reflected the German population in age, gender, educational level, and region. The Society for Social Research and Statistical Analysis Ltd., or forsa [22], collected the data in accordance with German data protection laws. Contact details and quotas, including response rates, were deleted immediately following the interviews.

Sociodemographic characteristics are presented in Table 1. The data in all five samples were comparable, with the exception of educational level, as more respondents had a higher education level in the years 2020 and 2021.

**Table 1:** Sociodemographic characteristics from 2019 to 2023.

Demographic	Characteristics	2019 n	2020 n	2021 n	2022 n	2023 n
**Gender**	Male	489	490	489	490	489
	Female	511	510	511	511	511
**Age**	18-39 years	319	317	316	316	315
	40-59 years	348	321	324	348	332
	≥60 years	333	362	360	347	352
**Employment status**	Employed	524	551	537	514	516
	Unemployed*	476	449	463	487	484
**Chronic condition**	Yes	366	311	287	342	339
	No	634	689	713	658	660
**Self-rated health status**	Very good/ good	572	679	677	589	578
	Satisfactory/Not so good/ poor	428	321	323	411	422
**Number of prescription medications**	No	458	508	533	453	473
	Yes	542	508	480	548	527
**Education level**	Up to secondary school	618	341	320	522	565
	Tertiary/ university	355	637	663	295	382
**Insurance status**	Statutory health insurance	875	786	807	859	875
	Private health insurance	125	214	193	141	125

*Economically inactive

### Ethical considerations

Ethical approval was not required for this study, as general public survey studies in Germany are exempt from ethical review requirements [23]. The polling institute forsa, mentioned above, signed the international ethics code for public opinion research (ICC/ESOMAR Code). The survey was conducted anonymously, participation was voluntary, and no financial compensation was provided. All participants gave their oral informed consent before beginning each survey (documented by the surveyor).

### Questionnaire

The TK Monitor of Patient Safety survey was developed in collaboration with experts, including patient safety researchers, social scientists, medical practitioners, health scientists, and psychologists. The questions and response items were designed drawing on existing surveys found in the literature [24–27] and developer discussions. The questionnaire was reviewed, pre-tested and validated by a panel of survey methodology experts, and minor adjustments made. It included closed-ended questions grouped into three sections (S2 Appendix). Section A focused on perceptions, experiences, and subjective information relating to patient safety in medical care, with responses scored on a Likert scale that ranged from “very likely” to “unlikely”. Section B treated perceptions about special issues, while Section C collected sociodemographic and socioeconomic data. Sections A and C remained largely the same from one year to the next, while Section B varied with each survey. The data reported here stem from sections A and C.

### Data analysis

Statistical analyses included descriptive statistics, chi-square tests comparing variables across the five surveys, and linear regressions for trend analysis. Since all explanatory variables were treated as categorical, absolute and relative frequencies were calculated, and associations were tested using the chi-squared test or Fisher’s exact test if any expected cell frequency was 5 or less. To examine trends over time, a Mantel-Haenszel chi-squared test for linear trend was applied to assess whether a linear association existed between the variables. This test is appropriate for assessing trends in ordinal or binary outcomes over time. For response variables with more than two categories, pairwise comparisons of p-values were conducted, with adjustments for multiple testing using the Benjamini & Hochberg (1995) method (suitable for non-normally distributed variables) [28]. Data were weighted based on gender, age, education level, and urban/rural population distribution, utilizing iterative proportional fitting. A two-sided p value of less than 0.05 was considered indicative of statistical significance. All analyses were conducted using the compareGroups R package (version 4.9.1) [29].

For trend visualization, locally weighted regression (LOESS) was applied using the ggplot2 package (version 3.5.1) [30]. LOESS fits local polynomial regressions (typically of degree 2) to subsets of the data, weighted by proximity to the focal point. This approach yields a flexible, non-parametric curve that effectively captures gradual changes and nonlinear patterns over time [31].

## Results

### Patient safety perceptions

**Fig 1:**
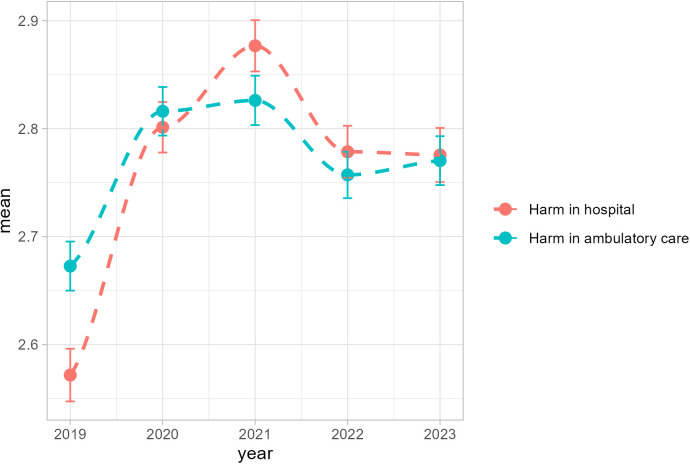
LOESS curves of participants’ perceptions of the likelihood of being harmed when receiving hospital or ambulatory care from 2019 to 2023 (N = 1000 per year).

A significant linear trend was observed in participants’ perceptions of the likelihood of being harmed when receiving hospital care (Table 2). Up to one third of respondents considered it very likely or somewhat likely that patients would be harmed if they received medical treatment in hospital, with a significantly lower mean-level before the COVID-19 pandemic in 2019 (Fig 1 and S3 Appendix). A similar pattern with a significantly lower mean-level in 2019 was observed in the assessment of risk associated with ambulatory care, however, no statistically significant linear trend was found over the years.

**Table 2:** Participants’ perceptions of the likelihood of being harmed when receiving hospital or ambulatory care from 2019 to 2023 (N = 1000 per year).

	2019	2020	2021	2022	2023	p overall	p trend
**Likelihood of being harmed when receiving ambulatory care** ^**a**^						**< 0.05**	0.06
Very likely (%)	9	7	11	7	7		
Somewhat likely (%)	30	24	21	25	24		
As likely as not (%)	53	58	57	60	58		
Unlikely (%)	7	10	10	7	9		
Not specified (%)	0	1	1	0	1		
**Likelihood of being harmed when receiving hospital care** ^**b**^						**< 0.05**	**< 0.05**
Very likely (%)	10	8	8	11	10		
Somewhat likely (%)	35	24	19	20	21		
As likely as not (%)	47	57	55	58	57		
Unlikely (%)	7	10	18	11	11		
Not specified (%)	1	1	0	0	1		

^a^How likely do you think it is that patients will be harmed by medical care provided outside hospital in Germany, e.g., when being treated as an outpatient at a doctor’s surgery?

^b^How likely do you think it is that patients in Germany will come to harm as a result of medical treatment in hospital?

### Risk perception

With regard to participants’ perceptions of the likelihood of adverse events, significant linear trends were observed for hospital-acquired infection, error during operation, and adverse events involving medical devices, with significant lower mean-rates before the COVID-19 pandemic in 2019 (Table 3 and S3 Appendix). Over half of respondents considered it very likely or somewhat likely that an illness would be diagnosed incorrectly at some stage in their lives (59% in 2019, 51% in 2020, 53% in 2021, 50% in 2022, 61% in 2023). Approximately two thirds of respondents believed that patient harm is largely preventable if appropriate measures are taken, with significant linear trends for error during operation, and medical device adverse events (Table 3).

**Table 3:** Participants’ perceptions of the likelihood of adverse events and their prevention from 2019 to 2023 (N = 1000 per year).

	Likelihood of adverse events^a^							Likelihood of prevention of adverse events^b^						
	2019	2020	2021	2022	2023	p overall	p trend	2019	2020	2021	2022	2023	p overall	p trend
**Hospital-acquired infection**														
Yes, definitely (%)	16	12	15	10	12	**< 0.05**	**< 0.05**	21	13	13	13	16	**< 0.05**	0.09
Yes, probably (%)	47	44	47	50	45			43	47	54	39	45		
Probably not (%)	31	35	33	35	37			29	33	27	38	33		
Certainly not (%)	6	8	4	4	6			7	6	6	10	6		
Not specified (%)	1	1	2	1	0			0	0	0	0	0		
**Incorrect diagnosis**														
Yes, definitely (%)	14	14	13	11	17	**< 0.05**	0.43	17	15	16	14	15	**< 0.05**	0.79
Yes, probably (%)	45	36	40	39	43			42	44	45	39	41		
Probably not (%)	36	42	42	45	33			34	34	33	39	38		
Certainly not (%)	5	6	5	5	6			6	6	6	8	6		
Not specified (%)	0	1	0	0	0			1	1	1	0	1		
**Error during operation**														
Yes, definitely (%)	6	7	8	5	6	**< 0.05**	**< 0.05**	16	14	15	15	15	**< 0.05**	**< 0.05**
Yes, probably (%)	36	24	27	20	23			39	45	43	40	45		
Probably not (%)	51	55	55	65	59			36	31	33	36	34		
Certainly not (%)	7	13	9	10	12			7	8	8	8	5		
Not specified (%)	0	1	1	0	0			1	1	1	1	0		
**Medication error**														
Yes, definitely (%)	8	8	12	7	10	**< 0.05**	0.08	20	19	19	19	18	**< 0.05**	0.08
Yes, probably (%)	40	30	32	26	33			42	37	45	40	41		
Probably not (%)	43	48	45	54	46			32	36	29	32	36		
Certainly not (%)	9	13	11	13	10			5	8	6	8	4		
Not specified (%)	0	0	0	0	1			1	1	1	0	0		
**Medical device adverse event**														
Yes, definitely (%)	5	3	3	1	3	**< 0.05**	**< 0.05**	18	20	18	18	20	**< 0.05**	**< 0.05**
Yes, probably (%)	26	20	21	16	19			41	40	47	44	41		
Probably not (%)	58	62	62	66	63			31	30	28	29	32		
Certainly not (%)	12	14	12	17	14			7	8	6	8	5		
Not specified (%)	0	1	2	0	1			2	2	2	0	1		

^a^How likely is it that the following will happen to you?

^b^Could the following be largely avoided in the future?

### Patient safety knowledge and self–efficacy

**Fig 2:**
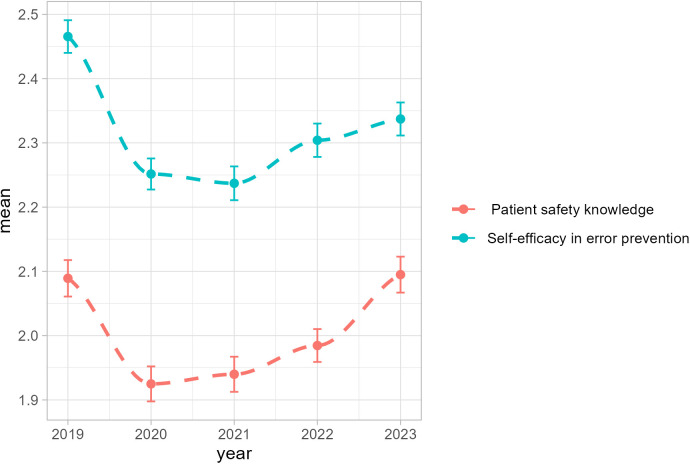
LOESS curves of participants’ perceptions relating to patient safety knowledge and self-efficacy in error prevention from 2019 to 2023 (N = 1000 per year).

A significant linear trend was observed in participants’ perception relating to patient safety knowledge (Table 4). From 2019 to 2023, the majority of respondents rated their level of knowledge about patient safety as good or moderate (68% in 2020, 69% in 2021, 64% in 2022, 63% in 2023) with a significant lower mean-rate during the COVID-19 pandemic in 2020 than pre- and post-COVID-19 pandemic rates (Fig 2 and S3 Appendix). Over the years of the study, up to three-quarters of respondents indicated a moderate to high self-efficacy level regarding the prevention of medical errors, with significantly lower mean-rates during the COVID-19 pandemic from 2020 to 2022 than in 2019 and 2023 (S3 Appendix). However, no statistically significant linear trend was found for participants’ perceptions relating to self-efficacy in error prevention over the years surveyed (Table 4).

**Table 4:** Participants’ perceptions relating to patient safety knowledge and self-efficacy in error prevention from 2019 to 2023 (N = 1000 per year).

	2019	2020	2021	2022	2023	p overall	p trend
**Self-efficacy in error prevention** ^**a**^						**<0.05**	0.40
Yes, definitely (%)	26	34	34	30	29		
Yes, probably (%)	43	41	40	44	41		
Probably not (%)	23	20	18	22	23		
Not at all (%)	6	5	8	4	6		
Not specified (%)	2	0	0	0	0		
**Patient safety knowledge** ^**b**^						**<0.05**	**<0.05**
Good (%)	9	11	18	14	12		
Moderate (%)	46	57	51	50	51		
Poor (%)	34	24	23	27	30		
None at all (%)	11	8	8	9	7		
Not specified (%)	0	0	1	0	0		

^a^Do you think that as a patient you can contribute towards improving the care you receive at the doctor’s surgery or in hospital?

^b^How well informed do you feel you are about patient safety in general?

## Discussion

This study showed a high level of perceived patient safety risk with lower levels before the COVID-19 pandemic, among the general German population. During the COVID-19 pandemic, perceived levels of knowledge and self-efficacy were lower than pre- and post-COVID-19 pandemic levels. From 2019 to 2023, every third respondent considered harm from treatment as likely, both in ambulatory settings and hospitals, with a significant lower mean-level before the COVID-19 pandemic. Risk levels of common adverse events, such as hospital-acquired infection, and incorrect diagnosis, were generally perceived to be high. Although this study focuses on patient safety perceptions in Germany, the observed trends resonate with international findings [32,33]. Worldwide, healthcare-related harm affects one out of every ten patients in healthcare [1]. There is limited data on preventable adverse events in primary and ambulatory settings. Moreover, studies use different definitions and methods to measure and report, thus highlighting the challenges in consistently identifying preventable adverse events. In 2008, the Commonwealth Fund, an American foundation, surveyed adults with significant health needs across eight countries to compare various aspects of healthcare quality [34]. Reported treatment errors varied slightly between countries: 9% of respondents in France reported experiencing errors, 8% in the Netherlands, and 10% in the UK. Comparisons with data from Australia reveal considerably lower incident rates in general practice settings, potentially reflecting differences in reporting systems, healthcare structures, and public awareness. In one study, 86 Australian general practitioners anonymously reported critical incidents from their practices over a 12-month period, resulting in a reported rate of approximately two events per 1,000 consultations per year [35]. This result seems to be much lower than the 14.2% rate of over-40-year olds in Germany who experienced a patient safety incident in ambulatory care in the last 12 months [36]. In our study, the majority of respondents believe that the adoption of appropriate measures can prevent patient harm. Indeed, there is evidence that up to 80% of the cases of harm can be avoided in primary and ambulatory settings [37].

In hospital settings, operative adverse events are more common but less preventable than diagnostic adverse events [38]. A systematic review of studies from the USA, Canada, the UK, Australia and New Zealand in 2008 found that adverse events during hospital admission affect nearly one out of ten patients [39]. Results of the Harvard Medical Practice Study revealed through retrospective patient chart reviews that 3.7% of patients in the state of New York experienced adverse events in hospitals [40], with 58% of these events deemed avoidable. Germany has not yet conducted a similar study, however the scale of data from the USA has been corroborated by studies in the UK [41].

Similar perceptions of being well informed and having a high level of self-efficacy in error prevention have also been reported in America [42,43]. In agreement with a study conducted in the USA [43], the majority of respondents to the TK survey considered themselves well informed and indicated a high level of self-efficacy in error prevention with lower mean-rates during the COVID-19 pandemic. In fact, public perception shifted from confidence in quality care before the COVID-19 pandemic [44] to uncertainty during the pandemic, as patients had concerns about overwhelmed hospitals and shortages of personal protective equipment [45]. After the pandemic, there was a growing acceptance of telemedicine, and public perception has again shifted, toward a balanced confidence based on transparency and patient involvement [11]. This increased engagement, along with the adoption of digital health solutions and a stronger emphasis on self-efficacy in error prevention, mirrors developments in other high-income countries [46,47]. Informed and empowered patients play an active role in safeguarding their own care. Addressing barriers to self-efficacy and improving knowledge through education, support, and transparent communication are essential steps in building a safer healthcare environment.

Several factors should be taken into account when interpreting the results. Firstly, like all interviewer-based surveys, telephone interviews are prone to social desirability bias, potentially leading to more favorable reports on patient safety issues than may actually exist. However, the survey questions primarily focused on participants’ perceptions rather than their attitudes toward patient safety. Secondly, the questionnaire was developed based on a literature review in the field of patient safety although it has not been validated in research studies. Nonetheless, the selection of survey questions and response options underwent a rigorous development process to ensure that content, structure, and wording were suitable for respondents. Leading German patient safety researchers contributed to the content development, and we employed the same survey questions over five consecutive years, so allowing for an assessment of patterns and trends in patient safety. Thirdly, the contact database was deleted immediately after data collection in compliance with data protection regulations, making it impossible to ascertain the response rate and the reasons for non-participation. This reduces transparency, makes replication difficult, and limits the generalizability of the findings. The perspectives of those who chose to participate may differ from those who did not. However, the samples remain representative of the German population in terms of age, gender, educational level, and region.

A strength of our study is the large sample size and the selection framework used for the ADM sample, which is a well-established research tool for high-quality random samples from the general population [48]. Moreover, conducting telephone interviews constitutes a fully standardized survey method, enabling efficient and relatively fast data collection. The CATI-based data collection technique minimizes item non-response [49]. Additionally, potential interviewer effects are less pronounced in telephone interviews than face-to-face surveys [50].

In recent years, advanced analytic methods based on machine learning (ML) and deep learning (DL) have demonstrated promising potential for improving patient safety [51]. ML techniques can support error detection, medication management, and monitoring of hospital-acquired infections [52], while DL approaches can enhance image-based diagnostics [53]. Future research should focus on integrating these technologies into clinical workflows in a transparent, ethical, and evidence-based manner to reduce preventable harm and to address public concerns about patient safety.

## Conclusions

This study examines trends in public perception of patient safety before, during, and after the COVID-19 pandemic from 2019 to 2023 in Germany, and emphasizes the critical need to build more resilient healthcare system. The results illustrate a high level of perceived patient safety risk among the German public. Over the five-year period, respondents felt well informed about patient safety and reported high self-efficacy with regard to error prevention, with lower levels during the COVID-19 pandemic. As patient safety continues to be a top priority in the German healthcare system, involving patients in safety efforts and maintaining open, transparent communication will be crucial in shaping positive public perception.

## Supporting information

S1 Appendix STROBE Statement – Checklist of items that should be included in reports of observational studies. (PDF)

S2 Appendix Questionnaire – TK Monitor of Patient Safety 2023. (PDF)

S3 Appendix Statistical Analyses – Linear regression models and LOESS curves. (PDF)

S4 Appendix Data sets for all samples. (PDF)
