# Supplementary material for: Trends in public perceptions of patient safety during the COVID-19 pandemic: Findings from a repeated cross-sectional survey in Germany, 2019–2023
Source: PLoS One. 2025 Aug 5;20(8):e0329761. doi: 10.1371/journal.pone.0329761 (PMC12324127; doi:10.1371/journal.pone.0329761)
Supplement: S1 Appendix — (PDF) [file pone.0329761.s001.pdf]

STROBE Statement—checklist of items that should be included in reports of observational studies

	Item No.	Recommendation	Page No.	Relevant text from manuscript
**Title and abstract**	1	(*a*) Indicate the study's design with a commonly used term in the title or the abstract	1	Findings from a repeated cross-sectional survey in Germany in 2019-2023
		(*b*) Provide in the abstract an informative and balanced summary of what was done and what was found	2	Abstract
**Introduction**				
Background/rationale	2	Explain the scientific background and rationale for the investigation being reported	3-4	Background
Objectives	3	State specific objectives, including any prespecified hypotheses	4	The main objective of the study is hence to explore nationwide patterns and trends in perceived patient safety, knowledge and self-efficacy from a population perspective before, during, and after the COVID-19 pandemic from 2019 to 2023 in Germany.
**Methods**				
Study design	4	Present key elements of study design early in the paper	4	This study draws on the Techniker Krankenkasse (TK) Monitor of Patient Safety, an annual large-scale national survey study designed to analyze the German general public's perception of patient safety trends.
Setting	5	Describe the setting, locations, and relevant dates, including periods of recruitment, exposure, follow-up, and data collection	4	The initial survey was conducted between October and November 2019, with later

				surveys in August 2020, June 2021, April to May 2022, and June 2023. Selected findings from these have been published in various reports.
Participants	6	(*a*) *Cohort study*—Give the eligibility criteria, and the sources and methods of selection of participants. Describe methods of follow-up<br>*Case-control study*—Give the eligibility criteria, and the sources and methods of case ascertainment and control selection. Give the rationale for the choice of cases and controls<br>*Cross-sectional study*—Give the eligibility criteria, and the sources and methods of selection of participants	4-5	The inclusion criteria required participants to be at least 18 years old, and to live in a private household (70.37 million adults living in Germany in 2023). The exclusion criterion was insufficient German language skills.<br>For selection of household respondents, we applied the last-birthday method, which identifies the household member with the most recent birthday.
		(*b*) *Cohort study*—For matched studies, give matching criteria and number of exposed and unexposed<br>*Case-control study*—For matched studies, give matching criteria and the number of controls per case		n.a.
Variables	7	Clearly define all outcomes, exposures, predictors, potential confounders, and effect modifiers. Give diagnostic criteria, if applicable	5	Section A focused on perceptions, experiences, and subjective information relating to patient safety in medical care, with responses scored on a Likert scale that ranged from "very likely" to "unlikely". Section B treated perceptions

				about special issues, while Section C collected sociodemographic and socioeconomic data. Sections A and C remained largely the same from one year to the next, while Section B varied with each surveyy. The data reported here stem from sections A and C.
Data sources/ measurement	8*	For each variable of interest, give sources of data and details of methods of assessment (measurement). Describe comparability of assessment methods if there is more than one group	4	Self-reported data were collected from nationally representative samples of 1,000 adults in each survey wave from 2019 to 2023.
Bias	9	Describe any efforts to address potential sources of bias	5	Contact details and quotas, including response rates, were deleted immediately following the interviews.
Study size	10	Explain how the study size was arrived at	4	This study draws on the Techniker Krankenkasse (TK) Monitor of Patient Safety, an annual large-scale national survey study designed to analyze the German general public's perception of patient safety trends. Detailed information about the study design can be found in other sources [15, 19]. It collects population-related data on

				perceptions, experiences, and knowledge related to patient safety from 1,000 randomly selected participants through computer-assisted telephone interviews (CATI).
Quantitative variables	11	Explain how quantitative variables were handled in the analyses. If applicable, describe which groupings were chosen and why	5	Data were weighted based on gender, age, education level, and urban/rural population distribution, utilizing iterative proportional fitting.
Statistical methods	12	(*a*) Describe all statistical methods, including those used to control for confounding	5	Statistical analyses included descriptive statistics, chi-square tests comparing variables across the five surveys, and linear regressions for trend analysis.
		(*b*) Describe any methods used to examine subgroups and interactions	5	Smoothed loess curves were also plotted to illustrate the trend. Loess curves constitute a flexible, nonparametric method of describing associations between two variables that makes no assumptions about data distribution.
		(*c*) Explain how missing data were addressed	5	Contact details and quotas, including response rates, were deleted immediately following the interviews.
		(*d*) *Cohort study*—If applicable, explain how loss to follow-up was addressed<br>*Case-control study*—If applicable, explain how matching of cases and controls was addressed		n.a.

		Cross-sectional study—If applicable, describe analytical methods taking account of sampling strategy		
		(*e*) Describe any sensitivity analyses		
**Results**				
Participants	13*	(a) Report numbers of individuals at each stage of study—eg numbers potentially eligible, examined for eligibility, confirmed eligible, included in the study, completing follow-up, and analysed	4	The inclusion criteria required participants to be at least 18 years old, and to live in a private household (70.37 million adults living in Germany in 2023). The exclusion criterion was insufficient German language skills.
		(b) Give reasons for non-participation at each stage		
		(c) Consider use of a flow diagram		
Descriptive data	14*	(a) Give characteristics of study participants (eg demographic, clinical, social) and information on exposures and potential confounders		Table 1
		(b) Indicate number of participants with missing data for each variable of interest	5	Contact details and quotas, including response rates, were deleted immediately following the interviews.
		(c) *Cohort study*—Summarise follow-up time (eg, average and total amount)		
Outcome data	15*	*Cohort study*—Report numbers of outcome events or summary measures over time		Table 1
		Case-control study—Report numbers in each exposure category, or summary measures of exposure		
		Cross-sectional study—Report numbers of outcome events or summary measures		
Main results	16	(*a*) Give unadjusted estimates and, if applicable, confounder-adjusted estimates and their precision (eg, 95% confidence interval). Make clear which confounders were adjusted for and why they were included		Table 2-3
		(*b*) Report category boundaries when continuous variables were categorized		
		(*c*) If relevant, consider translating estimates of relative risk into absolute risk for a meaningful time period		
Other analyses	17	Report other analyses done—eg analyses of subgroups and interactions, and sensitivity analyses		

**Discussion**				
Key results	18	Summarise key results with reference to study objectives	11	This study showed a high level of perceived patient safety risk with lower levels before the COVID-19 pandemic, among the general German population.
Limitations	19	Discuss limitations of the study, taking into account sources of potential bias or imprecision. Discuss both direction and magnitude of any potential bias	12	Strengths and limitations of the study
Interpretation	20	Give a cautious overall interpretation of results considering objectives, limitations, multiplicity of analyses, results from similar studies, and other relevant evidence	12	Strengths and limitations of the study
Generalisability	21	Discuss the generalisability (external validity) of the study results	12-13	Thirdly, the contact database was deleted immediately after data collection in compliance with data protection regulations, making it impossible to ascertain the response rate and the reasons for non-participation. This has implications for the generalizability of the findings. The perspectives of those who chose to participate may differ from those who did not. However, the samples remain representative of the German population in terms of age, gender, educational level, and region.
**Other information**				
Funding	22	Give the source of funding and the role of the funders for the present study and, if applicable, for the original study on which the present article is based	15	The TK Monitor of Patient Safety is funded by the statutory health fund Techniker Krankenkasse, Germany. The funding source had no influence on the decision to publish

	this manuscript or on the interpretation of the data. Award/grant number: Not applicable.

*Give information separately for cases and controls in case-control studies and, if applicable, for exposed and unexposed groups in cohort and cross-sectional studies.

Note: An Explanation and Elaboration article discusses each checklist item and gives methodological background and published examples of transparent reporting. The STROBE checklist is best used in conjunction with this article (freely available on the Web sites of PLoS Medicine at http://www.plosmedicine.org/, Annals of Internal Medicine at http://www.annals.org/, and Epidemiology at http://www.epidem.com/). Information on the STROBE Initiative is available at www.strobe-statement.org.
