# Supplementary material for: Trends in public perceptions of patient safety during the COVID-19 pandemic: Findings from a repeated cross-sectional survey in Germany, 2019–2023
Source: PLoS One. 2025 Aug 5;20(8):e0329761. doi: 10.1371/journal.pone.0329761 (PMC12324127; doi:10.1371/journal.pone.0329761)
Supplement: S3 Appendix — (PDF) [file pone.0329761.s003.pdf]

**Table A.** Linear regression models of participants' perceptions of patient safety between 2019 and 2023.

| Item | 2019 vs 2020 | 2019 vs 2021 | 2019 vs 2022 | 2019 vs 2023 | 2020 vs 2021 | 2020 vs 2022 | 2020 vs 2023 | 2021 vs 2022 | 2021 vs 2023 | 2022 vs 2023 |
|---|---|---|---|---|---|---|---|---|---|---|
| **Harm in** | | | | | | | | | | |
| Hospital | <0.001 | <0.001 | <0.001 | <0.001 | 0.098 | 0.640 | 0.247 | 0.051 | 0.051 | 0.665 |
| Ambulatory care | <0.001 | <0.001 | 0.054 | 0.025 | 0.761 | 0.152 | 0.562 | 0.025 | 0.152 | 0.628 |
| **Likelihood of** | | | | | | | | | | |
| Hospital-acquired infection | <0.001 | 0.337 | 0.002 | 0.019 | 0.019 | 0.264 | 0.535 | 0.157 | 0.308 | 0.308 |
| Incorrect diagnosis | 0.001 | 0.001 | 0.011 | 0.320 | 0.338 | 0.252 | 0.001 | 0.762 | 0.001 | 0.001 |
| Error during operation | <0.001 | <0.001 | <0.001 | <0.001 | 0.244 | 0.002 | 0.350 | 0.081 | 0.541 | 0.081 |
| Medication error | <0.001 | <0.001 | <0.001 | 0.085 | 0.260 | 0.450 | 0.019 | 0.450 | 0.127 | 0.009 |
| Medical device adverse event | <0.001 | 0.009 | <0.001 | 0.001 | 0.238 | 0.410 | 0.410 | 0.079 | 0.929 | 0.238 |
| **Prevention of** | | | | | | | | | | |
| Hospital-acquired infection | 0.005 | 0.018 | <0.001 | 0.040 | 0.673 | 0.002 | 0.047 | <0.001 | 0.057 | 0.013 |
| Incorrect diagnosis | 0.794 | 0.794 | 0.794 | 0.794 | 0.794 | 0.794 | 0.794 | 0.794 | 0.794 | 0.794 |
| Error during operation | 0.721 | 0.721 | 0.900 | 0.496 | 0.721 | 0.721 | 0.702 | 0.875 | 0.721 | 0.721 |
| Medication error | 0.153 | 0.951 | 0.782 | 0.153 | 0.324 | 0.356 | 0.095 | 0.810 | 0.153 | 0.469 |
| Medical device adverse event | 0.258 | 0.838 | 0.235 | 0.258 | 0.365 | 0.235 | 0.301 | 0.448 | 0.579 | 0.838 |
| **Self-efficacy in error prevention** | 0.003 | 0.006 | 0.010 | 0.981 | 0.981 | 0.047 | 0.002 | 0.077 | 0.003 | 0.010 |
| **Patient safety knowledge** | <0.001 | <0.001 | <0.001 | 0.005 | 0.007 | 0.006 | 0.002 | 0.366 | 0.027 | 0.539 |

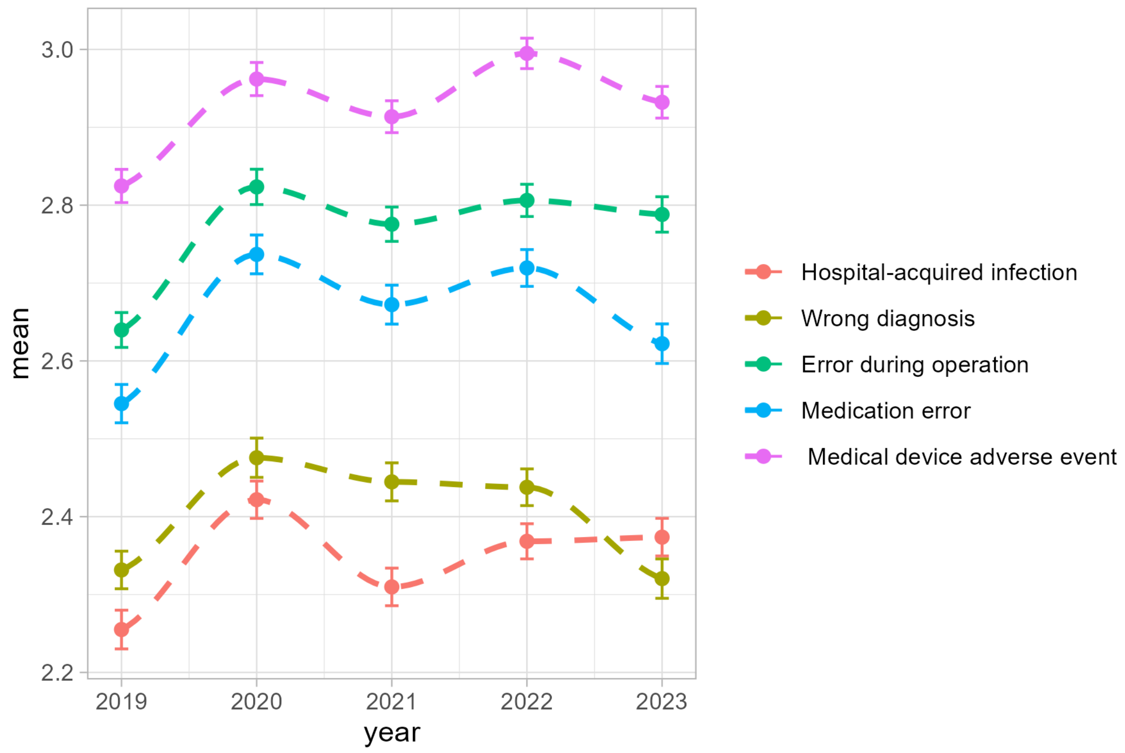


**Figure A.** LOESS curve of participants' perceptions of the likelihood of adverse events from 2019 to 2023.

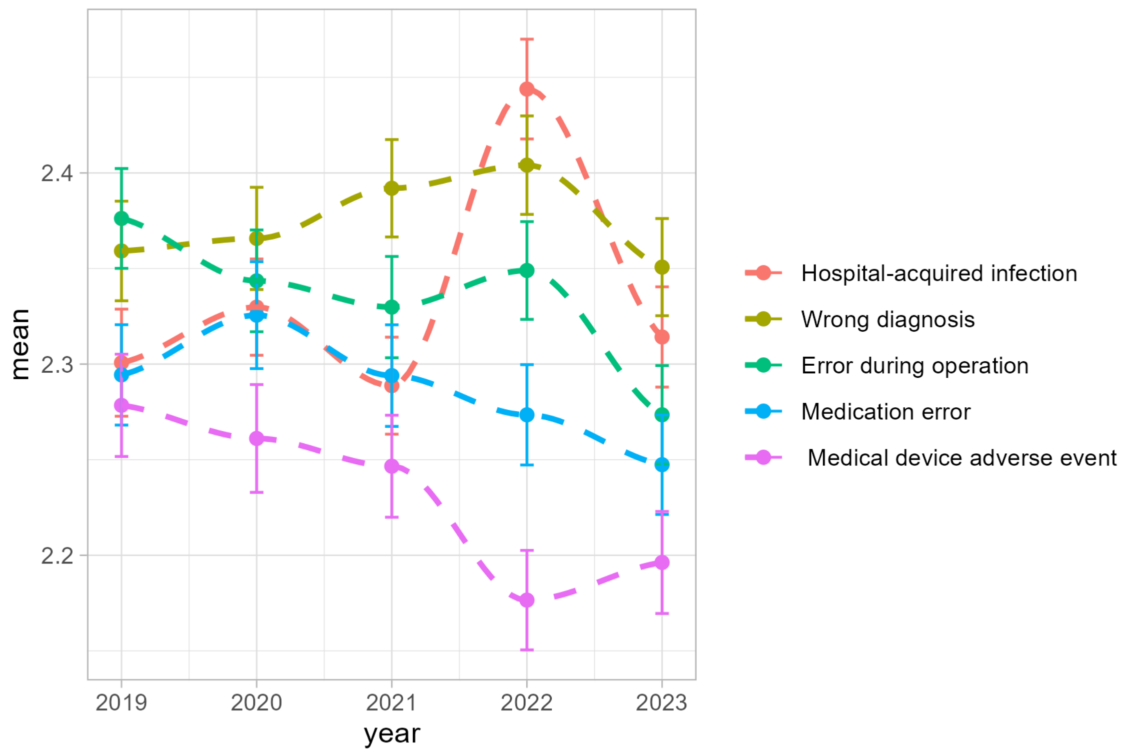


**Figure B.** LOESS curves of participants' perceptions of the likelihood of adverse events prevention from 2019 to 2023.

**Table B.** Medians and first-third quartiles [Q1–Q3] between 2019 and 2023.

| Item | 2019<br>*N=941* | 2020<br>*N=946* | 2021<br>*N=949* | 2022<br>*N=980* | 2023<br>*N=958* | p. overall |
|---|---|---|---|---|---|---|
| **Harm in** | | | | | | |
| Hospital | 3.00 [2.00;3.00] | 3.00 [2.00;3.00] | 3.00 [3.00;3.00] | 3.00 [2.00;3.00] | 3.00 [2.00;3.00] | <0.001 |
| Ambulatory care | 3.00 [2.00;3.00] | 3.00 [2.25;3.00] | 3.00 [3.00;3.00] | 3.00 [2.00;3.00] | 3.00 [2.00;3.00] | <0.001 |
| **Likelihood of** | | | | | | |
| Hospital-acquired infection | 2.00 [2.00;3.00] | 2.00 [2.00;3.00] | 2.00 [2.00;3.00] | 2.00 [2.00;3.00] | 2.00 [2.00;3.00] | <0.001 |
| Incorrect diagnosis | 2.00 [2.00;3.00] | 3.00 [2.00;3.00] | 3.00 [2.00;3.00] | 2.00 [2.00;3.00] | 2.00 [2.00;3.00] | <0.001 |
| Error during operation | 3.00 [2.00;3.00] | 3.00 [2.00;3.00] | 3.00 [2.00;3.00] | 3.00 [3.00;3.00] | 3.00 [2.00;3.00] | <0.001 |
| Medication error | 3.00 [2.00;3.00] | 3.00 [2.00;3.00] | 3.00 [2.00;3.00] | 3.00 [2.00;3.00] | 3.00 [2.00;3.00] | <0.001 |
| Medical device adverse event | 3.00 [2.00;3.00] | 3.00 [3.00;3.00] | 3.00 [3.00;3.00] | 3.00 [3.00;3.00] | 3.00 [3.00;3.00] | <0.001 |
| **Prevention of** | | | | | | |
| Hospital-acquired infection | 2.00 [2.00;3.00] | 2.00 [2.00;3.00] | 2.00 [2.00;3.00] | 2.00 [2.00;3.00] | 2.00 [2.00;3.00] | <0.001 |
| Incorrect diagnosis | 2.00 [2.00;3.00] | 2.00 [2.00;3.00] | 2.00 [2.00;3.00] | 2.00 [2.00;3.00] | 2.00 [2.00;3.00] | 0.498 |
| Error during operation | 2.00 [2.00;3.00] | 2.00 [2.00;3.00] | 2.00 [2.00;3.00] | 2.00 [2.00;3.00] | 2.00 [2.00;3.00] | 0.082 |
| Medication error | 2.00 [2.00;3.00] | 2.00 [2.00;3.00] | 2.00 [2.00;3.00] | 2.00 [2.00;3.00] | 2.00 [2.00;3.00] | 0.463 |
| Medical device adverse event | 2.00 [2.00;3.00] | 2.00 [2.00;3.00] | 2.00 [2.00;3.00] | 2.00 [2.00;3.00] | 2.00 [2.00;3.00] | 0.046 |
| **Self-efficacy in error prevention** | 2.00 [1.00;3.00] | 2.00 [1.00;2.00] | 2.00 [1.00;2.00] | 2.00 [1.00;2.25] | 2.00 [1.00;3.00] | <0.001 |
| **Patient safety knowledge** | 2.00 [2.00;3.00] | 2.00 [2.00;3.00] | 2.00 [2.00;3.00] | 2.00 [2.00;3.00] | 2.00 [2.00;3.00] | <0.001 |
