# Supplementary material for: Trends in public perceptions of patient safety during the COVID-19 pandemic: Findings from a repeated cross-sectional survey in Germany, 2019–2023
Source: PLoS One. 2025 Aug 5;20(8):e0329761. doi: 10.1371/journal.pone.0329761 (PMC12324127; doi:10.1371/journal.pone.0329761)
Supplement: S4 Appendix — (PDF) [file pone.0329761.s004.pdf]

forsa.

TK Patient Safety Monitor: Time comparison 2019 to 2024

f24.0125 Sh/Cr/Ul, May 2024

Question 1: Not applicable

Question 2: 'Patient safety' is the successful endeavour to ensure error-free and harm-free medical treatment and medical healthcare. In your opinion, how likely is it that patients in Germany will come to harm as a result of medical treatment in hospital? Do you think this is 'very likely' - 'fairly likely' - 'not very likely' - or 'unlikely'?

	Total						Gender											
	2019	2020	2021	2022	2023	2024	2019 / Men	2020 / men	2021 / men	2022 / men	2023 / men	2024 / Men	2019 / women	2020 / women	2021 / Women	2022 / Women	2023 / Women	2024 / Women
Basis (100%)	1000	1000	1000	1001	1000	1000	489	489	490	490	489	488	511	511	511	511	511	512
very likely	10%	8%	8%	11%	10%	9%	9%	9%	4%	11%	7%	11%	11%	8%	12%	10%	13%	7%
Fairly likely	35%	24%	19%	20%	21%	22%	31%	21%	16%	16%	17%	17%	39%	26%	23%	24%	25%	26%
not very likely	47%	57%	55%	58%	57%	55%	49%	54%	63%	59%	63%	55%	44%	59%	47%	57%	52%	56%
unlikely	7%	10%	18%	11%	11%	13%	10%	13%	18%	15%	13%	17%	4%	7%	18%	8%	10%	10%
wn/kA	1%	1%			1%		1%	2%			-		1%		-		1%	
Sum	100%	100%	100%	100%	100%	100%	100%	100%	100%	100%	100%	100%	100%	100%	100%	100%	100%	100%
very/somewhat likely	45%	32%	27%	31%	31%	31%	39%	31%	19%	26%	24%	28%	51%	34%	35%	35%	37%	33%
not very likely/unlikely	54%	67%	73%	69%	69%	69%	59%	68%	80%	73%	76%	71%	48%	66%	65%	65%	62%	66%

Question 3: And in your opinion, how likely is it that
that patients are harmed by medical care outside of a hospital in

Germany, e.g. through outpatient treatment by a doctor or
incorrect medication? Do you think this is 'very likely' - 'fairly
likely' - 'not very likely'?
'fairly likely' - 'not very likely' -
or 'unlikely'?

Basis (100%)	1000	1000	1000	1001	1000	1000	489	489	490	490	489	488	511	511	511	511	511	512
very likely	9%	7%	11%	7%	7%	6%	9%	6%	4%	7%	6%	6%	10%	8%	17%	7%	8%	6%
Fairly likely	30%	24%	21%	25%	24%	24%	28%	21%	21%	22%	18%	24%	31%	28%	22%	29%	30%	24%
not very likely	53%	58%	57%	60%	58%	59%	52%	62%	63%	63%	64%	56%	54%	54%	51%	58%	54%	62%
unlikely	7%	10%	10%	7%	9%	9%	10%	10%	12%	8%	10%	12%	5%	10%	8%	6%	8%	7%
wn/kA		1%	1%		1%	1%		1%			2%	2%			2%		1%	1%
total	100%	100%	100%	100%	100%	100%	100%	100%	100%	100%	100%	100%	100%	100%	100%	100%	100%	100%
very/somewhat likely	39%	31%	32%	32%	31%	30%	37%	27%	24%	28%	24%	30%	41%	36%	39%	36%	38%	30%
not very likely/unlikely	61%	68%	67%	67%	67%	69%	63%	72%	75%	71%	74%	68%	59%	64%	59%	64%	61%	69%

Question 4: I will now tell you about possible harmful events in medical
care, which are also called
called 'adverse events'.

Please tell me in each case whether you think this will happen to you:
'yes, definitely' - 'yes, probably' - 'probably not' - or 'definitely not'?
How likely is it that the following will happen to you: ...?

Overview table: yes, definitely/probably

Basis (100%)	1000	1000	1000	1001	1000	1000	489	489	490	490	489	488	511	511	511	511	511	512
Infection with dangerous germs in hospital	63%	56%	62%	60%	57%	60%	57%	52%	54%	53%	55%	57%	68%	59%	69%	67%	59%	63%
an incorrect diagnosis	59%	51%	53%	50%	61%	54%	54%	51%	47%	45%	57%	52%	64%	50%	59%	55%	65%	55%
an error during an operation, e.g. a surgical error surgical error	42%	31%	34%	25%	29%	29%	38%	32%	25%	26%	28%	25%	45%	30%	43%	24%	29%	33%
an error with medicines due to incorrect prescription, incorrect dosage or incorrect method of use	49%	38%	44%	33%	42%	35%	43%	39%	36%	29%	38%	32%	53%	37%	52%	37%	47%	38%
an error caused by a medical treatment device	30%	22%	24%	17%	22%	17%	27%	20%	17%	16%	17%	15%	33%	24%	31%	18%	27%	19%

f24.0125 Sh/Cr/Ul, May 2024

Question 4: I would now like to name possible harmful incidents in medical care, which are also called called 'adverse events'.

Please tell me in each case whether you think this will happen to you: 'yes, definitely' - 'yes, probably' - 'probably not' - or 'definitely not'? How likely is it that the following will happen to you: ...?

... an infection with dangerous germs in hospital

	Total						Gender											
	2019	2020	2021	2022	2023	2024	2019 / Men	2020 / men	2021 / men	2022 / men	2023 / Men	2024 / Men	2019 / women	2020 / women	2021 / Women	2022 / Women	2023 / Women	2024 / Women
Basis (100%)	1000	1000	1000	1001	1000	1000	489	489	490	490	489	488	511	511	511	511	511	512
yes, definitely (incl. has already happened to me)	16%	12%	15%	10%	12%	14%	11%	14%	11%	8%	11%	14%	22%	9%	18%	11%	12%	14%
yes, probably	47%	44%	47%	50%	45%	46%	47%	38%	44%	44%	43%	44%	47%	50%	50%	56%	47%	49%
probably not	31%	35%	33%	35%	37%	35%	36%	38%	40%	40%	39%	36%	26%	32%	26%	30%	36%	33%
definitely not	6%	8%	4%	4%	6%	5%	6%	9%	5%	6%	6%	6%	6%	7%	2%	3%	5%	4%
wn/kA	1%	1%	2%	1%			1%	1%		1%		-		1%	3%	-		
Total	100%	100%	100%	100%	100%	100%	100%	100%	100%	100%	100%	100%	100%	100%	100%	100%	100%	100%
yes, definitely/probably	63%	56%	62%	60%	57%	60%	57%	52%	54%	53%	55%	57%	68%	59%	69%	67%	59%	63%
definitely not/probably not	36%	43%	37%	39%	43%	40%	42%	47%	45%	46%	45%	43%	32%	39%	28%	33%	41%	37%

Question 4: I will now tell you about possible harmful events in medical care, which are also called called 'adverse events'.

Please tell me in each case whether you think this will happen to you: 'yes, definitely' - 'yes, probably' - 'probably not' - or 'definitely not'? How likely is it that the following will happen to you: ...?

... an incorrect diagnosis

Basis (100%)	1000	1000	1000	1001	1000	1000	489	489	490	490	489	488	511	511	511	511	511	512
yes, definitely (incl. has already happened to me)	14%	14%	13%	11%	17%	13%	11%	12%	11%	9%	12%	10%	17%	17%	15%	13%	23%	16%
yes, probably	45%	36%	40%	39%	43%	41%	43%	39%	36%	37%	45%	42%	47%	34%	43%	41%	42%	40%
probably not	36%	42%	42%	45%	33%	38%	43%	43%	47%	50%	38%	37%	28%	42%	36%	40%	28%	38%
definitely not	5%	6%	5%	5%	6%	8%	3%	5%	6%	5%	5%	9%	7%	7%	5%	5%	7%	7%
wn/kA		1%				1%		1%				1%		1%		-		
Total	100%	100%	100%	100%	100%	100%	100%	100%	100%	100%	100%	100%	100%	100%	100%	100%	100%	100%
yes, definitely/probably	59%	51%	53%	50%	61%	54%	54%	51%	47%	45%	57%	52%	64%	50%	59%	55%	65%	55%
definitely not/probably not	41%	49%	47%	50%	39%	45%	46%	48%	53%	55%	43%	47%	35%	49%	41%	45%	35%	44%

Question 4: I will now tell you about possible harmful incidents in medical care, which are also called
called 'adverse events'.

Please tell me in each case whether you think this will happen to you: 'yes, definitely' - 'yes, probably' - 'probably not' - or 'definitely not'? How likely is it that the following will happen to you: ...?

... an error during an operation, e.g. a surgical error

Basis (100%)	1000	1000	1000	1001	1000	1000	489	489	490	490	489	488	511	511	511	511	511	512
yes, definitely (incl. has already happened to me)	6%	7%	8%	5%	6%	5%	5%	4%	6%	4%	7%	4%	8%	9%	10%	7%	4%	5%
yes, probably	36%	24%	27%	20%	23%	24%	33%	28%	20%	22%	21%	21%	38%	21%	34%	17%	25%	27%
probably not	51%	55%	55%	65%	59%	62%	55%	54%	66%	63%	60%	64%	48%	57%	46%	68%	58%	60%
definitely not	7%	13%	9%	10%	12%	9%	7%	14%	9%	11%	12%	10%	7%	12%	9%	8%	12%	7%
wn/kA		1%	1%											2%	2%			
total	100%	100%	100%	100%	100%	100%	100%	100%	100%	100%	100%	100%	100%	100%	100%	100%	100%	100%

f24.0125 Sh/Cr/Ul, May 2024	Total						Gender											
	2019	2020	2021	2022	2023	2024	2019 / Men	2020 / men	2021 / men	2022 / men	2023 / men	2024 / Men	2019 / women	2020 / women	2021 / Women	2022 / Women	2023 / Women	2024 / Women
yes, definitely/probably	42%	31%	34%	25%	29%	29%	38%	32%	25%	26%	28%	25%	45%	30%	43%	24%	29%	33%
definitely not/probably not	58%	68%	64%	75%	71%	71%	62%	67%	74%	74%	72%	75%	54%	69%	55%	76%	71%	67%

Question 4: I will now tell you about possible harmful events in medical care, which are also called
called 'adverse events'.

Please tell me in each case whether you think this will happen to you: 'yes, definitely' - 'yes, probably' - 'probably not' - or 'definitely not'?
How likely is it that the following will happen to you: ...?

... an error with medicines due to incorrect prescription, incorrect dosage or incorrect method of use

	2019	2020	2021	2022	2023	2024	2019 / Men	2020 / men	2021 / men	2022 / men	2023 / men	2024 / Men	2019 / women	2020 / women	2021 / Women	2022 / Women	2023 / Women	2024 / Women
Basis (100%)	1000	1000	1000	1001	1000	1000	489	489	490	490	489	488	511	511	511	511	511	512
yes, definitely (incl. has already happened to me)	8%	8%	12%	7%	10%	7%	5%	7%	8%	7%	4%	6%	11%	8%	16%	8%	15%	7%
yes, probably	40%	30%	32%	26%	33%	28%	38%	32%	28%	22%	34%	26%	42%	29%	36%	29%	32%	31%
probably not	43%	48%	45%	54%	46%	53%	48%	48%	54%	57%	51%	57%	38%	49%	38%	52%	41%	48%
definitely not	9%	13%	11%	13%	10%	12%	8%	13%	10%	14%	10%	10%	9%	14%	11%	11%	11%	14%
wn/kA					1%			1%			1%					-	1%	
total	100%	100%	100%	100%	100%	100%	100%	100%	100%	100%	100%	100%	100%	100%	100%	100%	100%	100%
yes, definitely/probably	49%	38%	44%	33%	42%	35%	43%	39%	36%	29%	38%	32%	53%	37%	52%	37%	47%	38%
definitely not/probably not	51%	62%	56%	67%	56%	65%	56%	60%	64%	71%	61%	68%	46%	63%	48%	63%	52%	62%

Question 4: I will now tell you about possible harmful events in medical care, which are also called
called 'adverse events'.

Please tell me in each case whether you think this will happen to you: 'yes, definitely' - 'yes, probably' - 'probably not' - or 'definitely not'?
How likely is it that the following will happen to you: ...?

... an error caused by a medical treatment device

Basis (100%)	1000	1000	1000	1001	1000	1000	489	489	490	490	489	488	511	511	511	511	511	512
yes, definitely (incl. has already happened to me)	5%	3%	3%	1%	3%	3%	4%	1%	2%	1%	3%	3%	5%	4%	5%	2%	4%	3%
yes, probably	26%	20%	21%	16%	19%	15%	23%	19%	15%	15%	14%	13%	29%	20%	26%	17%	23%	17%
probably not	58%	62%	62%	66%	63%	66%	61%	64%	68%	66%	64%	67%	55%	61%	57%	66%	62%	66%
definitely not	12%	14%	12%	17%	14%	16%	11%	15%	14%	18%	18%	18%	12%	14%	10%	16%	10%	14%
wn/kA		1%	2%		1%			1%	1%		1%			1%	2%		1%	1%
Total	100%	100%	100%	100%	100%	100%	100%	100%	100%	100%	100%	100%	100%	100%	100%	100%	100%	100%
yes, definitely/probably	30%	22%	24%	17%	22%	17%	27%	20%	17%	16%	17%	15%	33%	24%	31%	18%	27%	19%
definitely not/probably not	69%	77%	74%	83%	77%	82%	72%	79%	81%	84%	82%	84%	67%	75%	66%	82%	73%	80%

Question 5: I will now read these 'adverse events' to you again. Please tell me in each case whether you think that this can be largely avoided in future by 'suitable measures': 'yes, definitely' - 'yes, probably' - 'probably not' - or 'definitely not'? Can the following be largely avoided in future ... ?

Overview table: yes, definitely/probably

Basis (100%)	1000	1000	1000	1001	1000	1000	489	489	490	490	489	488	511	511	511	511	511	512
Infection with dangerous germs in hospital	64%	60%	67%	52%	62%	56%	61%	62%	68%	51%	62%	57%	66%	59%	66%	54%	62%	56%
an incorrect diagnosis	59%	59%	61%	53%	56%	59%	58%	58%	58%	52%	55%	53%	60%	59%	63%	53%	57%	65%
an error during an operation, e.g. a surgical error	55%	60%	58%	55%	60%	58%	54%	61%	56%	52%	59%	56%	56%	59%	60%	57%	62%	60%

f24.0125 Sh/Cr/Ul, May 2024	Total						Gender											
	2019	2020	2021	2022	2023	2024	2019 / Men	2020 / men	2021 / men	2022 / men	2023 / men	2024 / Men	2019 / women	2020 / women	2021 / Women	2022 / Women	2023 / Women	2024 / Women
an error with medicinal products due to incorrect prescription, incorrect dosage or incorrect method of use	62%	55%	64%	59%	59%	63%	61%	55%	66%	55%	59%	60%	63%	56%	62%	64%	60%	66%
an error caused by a medical treatment device	59%	60%	64%	62%	61%	64%	59%	57%	62%	61%	59%	61%	59%	63%	66%	63%	64%	66%

Question 5: I will now read these 'adverse events' to you again. Please tell me in each case whether you think that this can be largely avoided in future by
'suitable measures': 'yes, definitely' - 'yes, probably' - 'probably not' - or 'definitely not'? Can the following be largely avoided in future ... ?

... infection with dangerous germs in hospital

	Total						Gender											
	2019	2020	2021	2022	2023	2024	2019 / Men	2020 / men	2021 / men	2022 / men	2023 / men	2024 / Men	2019 / women	2020 / women	2021 / Women	2022 / Women	2023 / Women	2024 / Women
Basis (100%)	1000	1000	1000	1001	1000	1000	489	489	490	490	489	488	511	511	511	511	511	512
yes, definitely	21%	13%	13%	13%	16%	14%	18%	12%	12%	13%	15%	11%	24%	14%	15%	13%	18%	17%
yes, probably	43%	47%	54%	39%	45%	42%	43%	50%	56%	38%	47%	46%	42%	44%	51%	41%	44%	39%
probably not	29%	33%	27%	38%	33%	35%	32%	31%	27%	39%	33%	33%	26%	35%	28%	37%	32%	37%
definitely not	7%	6%	6%	10%	6%	8%	7%	6%	5%	11%	5%	10%	8%	6%	6%	9%	6%	7%
wn/kA		-	-	-	-	-		-	-	-	-	-	1%	-	-	-	-	-
Total	100%	100%	100%	100%	100%	100%	100%	100%	100%	100%	100%	100%	100%	100%	100%	100%	100%	100%
yes, definitely/probably	64%	60%	67%	52%	62%	56%	61%	62%	68%	51%	62%	57%	66%	59%	66%	54%	62%	56%
definitely not/probably not	36%	40%	33%	48%	38%	44%	39%	38%	32%	49%	38%	43%	34%	41%	34%	46%	38%	44%

Question 5: I will now read these 'adverse events' to you again. Please tell me in each case whether you think that this can be largely avoided in future by
'suitable measures': 'yes, definitely' - 'yes, probably' - 'probably not' - or 'definitely not'? Can the following be largely avoided in future ... ?

... an incorrect diagnosis

Basis (100%)	1000	1000	1000	1001	1000	1000	489	489	490	490	489	488	511	511	511	511	511	512
yes, definitely	17%	15%	16%	14%	15%	16%	15%	13%	13%	14%	15%	12%	19%	16%	19%	15%	15%	20%
yes, probably	42%	44%	45%	39%	41%	43%	43%	45%	45%	38%	40%	41%	41%	43%	45%	39%	43%	45%
probably not	34%	34%	33%	39%	38%	35%	36%	36%	34%	39%	39%	40%	32%	33%	32%	39%	37%	29%
definitely not	6%	6%	6%	8%	6%	6%	5%	5%	7%	9%	6%	7%	7%	8%	4%	7%	5%	5%
wn/kA	1%	1%	1%		1%			1%	1%	-			1%				1%	
total	100%	100%	100%	100%	100%	100%	100%	100%	100%	100%	100%	100%	100%	100%	100%	100%	100%	100%
yes, definitely/probably	59%	59%	61%	53%	56%	59%	58%	58%	58%	52%	55%	53%	60%	59%	63%	53%	57%	65%
definitely not/probably not	40%	41%	39%	47%	43%	41%	42%	40%	41%	48%	45%	47%	39%	41%	37%	46%	42%	35%

Question 5: I will now read these 'adverse events' to you again. Please tell me in each case whether you think that this can be largely avoided in future by 'suitable measures': 'yes, definitely' - 'yes, probably' - 'probably not' - or 'definitely not'? Can the following be largely avoided in future ... ?

... an error during an operation, e.g. a surgical error

Basis (100%)	1000	1000	1000	1001	1000	1000	489	489	490	490	489	488	511	511	511	511	511	512
yes, definitely	16%	14%	15%	15%	15%	17%	13%	14%	13%	13%	17%	16%	20%	14%	16%	16%	14%	17%
yes, probably	39%	45%	43%	40%	45%	41%	41%	46%	43%	39%	42%	40%	36%	45%	43%	41%	48%	43%
probably not	36%	31%	33%	36%	34%	34%	38%	30%	36%	37%	36%	32%	35%	32%	31%	36%	32%	36%
definitely not	7%	8%	8%	8%	5%	7%	7%	8%	7%	10%	5%	12%	8%	8%	9%	6%	6%	3%
wn/kA	1%	1%	1%	1%		1%	1%	2%	1%	1%			1%	1%				1%

f24.0125 Sh/Cr/Ul, May 2024	Total gender						Gender											
	2019	2020	2021	2022	2023	2024	2019 / Men	2020 / men	2021 / men	2022 / men	2023 / men	2024 / Men	2019 / women	2020 / women	2021 / Women	2022 / Women	2023 / Women	2024 / Women
Total	100%	100%	100%	100%	100%	100%	100%	100%	100%	100%	100%	100%	100%	100%	100%	100%	100%	100%
yes, definitely/probably	55%	60%	58%	55%	60%	58%	54%	61%	56%	52%	59%	56%	56%	59%	60%	57%	62%	60%
definitely not/probably not	44%	39%	41%	44%	40%	41%	45%	38%	43%	46%	41%	44%	43%	40%	40%	42%	38%	39%

Question 5: I will now read these 'adverse events' to you again. Please tell me in each case whether you think that this can be largely avoided in future by
'suitable measures': 'yes, definitely' - 'yes, probably' - 'probably not' - or 'definitely not'? Can the following be largely avoided in future ... ?

... an error with medicines due to incorrect prescription, incorrect dosage or incorrect method of use

	2019	2020	2021	2022	2023	2024	2019 / Men	2020 / men	2021 / men	2022 / men	2023 / men	2024 / Men	2019 / women	2020 / women	2021 / Women	2022 / Women	2023 / Women	2024 / Women
Basis (100%)	1000	1000	1000	1001	1000	1000	489	489	490	490	489	488	511	511	511	511	511	512
yes, definitely	20%	19%	19%	19%	18%	22%	16%	17%	19%	17%	17%	20%	25%	21%	19%	20%	19%	23%
yes, probably	42%	37%	45%	40%	41%	42%	45%	38%	47%	37%	42%	40%	39%	35%	43%	43%	41%	43%
probably not	32%	36%	29%	32%	36%	30%	33%	37%	27%	35%	37%	31%	30%	34%	30%	29%	36%	29%
definitely not	5%	8%	6%	8%	4%	6%	5%	8%	6%	10%	4%	9%	6%	8%	6%	7%	4%	3%
wn/kA	1%	1%	1%	-		1%	1%	1%	1%	-		-	1%	1%	2%	-		1%
total	100%	100%	100%	100%	100%	100%	100%	100%	100%	100%	100%	100%	100%	100%	100%	100%	100%	100%
yes, definitely/probably	62%	55%	64%	59%	59%	63%	61%	55%	66%	55%	59%	60%	63%	56%	62%	64%	60%	66%
definitely not/probably not	37%	44%	35%	41%	40%	36%	38%	44%	33%	45%	41%	40%	36%	43%	36%	36%	40%	32%

Question 5: I will now read these 'adverse events' to you again. Please tell me in each case whether you think that this can be largely avoided in future by
'suitable measures': 'yes, definitely' - 'yes, probably' - 'probably not' - or 'definitely not'? Can the following be largely avoided in future ... ?

... an error caused by a medical treatment device

Basis (100%)	1000	1000	1000	1001	1000	1000	489	489	490	490	489	488	511	511	511	511	511	512
yes, definitely	18%	20%	18%	18%	20%	22%	18%	18%	16%	19%	20%	23%	18%	22%	20%	18%	20%	20%
yes, probably	41%	40%	47%	44%	41%	42%	41%	40%	47%	43%	39%	38%	42%	40%	47%	46%	43%	46%
probably not	31%	30%	28%	29%	32%	29%	31%	31%	29%	31%	33%	29%	32%	29%	27%	28%	32%	29%
definitely not	7%	8%	6%	8%	5%	6%	8%	9%	7%	7%	7%	9%	7%	7%	5%	8%	4%	4%
wn/kA	2%	2%	2%		1%	1%	2%	2%	2%		1%		1%	2%	1%		1%	2%
Total	100%	100%	100%	100%	100%	100%	100%	100%	100%	100%	100%	100%	100%	100%	100%	100%	100%	100%
yes, definitely/probably	59%	60%	64%	62%	61%	64%	59%	57%	62%	61%	59%	61%	59%	63%	66%	63%	64%	66%
definitely not/probably not	39%	38%	34%	37%	38%	36%	39%	40%	36%	38%	40%	38%	39%	36%	32%	36%	36%	33%

Question 6 and question 7: Not applicable

Question 8: We have now talked a little about the topic of 'patient safety'. How well informed do you feel about patient safety in general: 'very well' - 'well' - 'less well' - or 'not informed at all'?
'well' - 'less well' - or 'not informed at all'?

Basis (100%)	1000	1000	1000	1001	1000	1000	489	489	490	490	489	488	511	511	511	511	511	512
very good	9%	11%	18%	14%	12%	12%	9%	10%	15%	13%	10%	10%	9%	11%	21%	16%	13%	14%
good	46%	57%	51%	50%	51%	49%	49%	54%	54%	49%	52%	45%	43%	60%	48%	52%	51%	53%
less good	34%	24%	23%	27%	30%	30%	31%	29%	24%	29%	30%	33%	37%	20%	21%	24%	30%	27%
Not informed at all	11%	8%	8%	9%	7%	8%	11%	6%	7%	9%	7%	11%	10%	10%	8%	9%	7%	6%
wn/kA			1%			1%									1%			1%
total	100%	100%	100%	100%	100%	100%	100%	100%	100%	100%	100%	100%	100%	100%	100%	100%	100%	100%
(very) good	55%	68%	69%	64%	63%	61%	57%	65%	69%	62%	62%	55%	53%	70%	69%	67%	64%	67%
less good/not at all	45%	32%	30%	35%	37%	38%	42%	35%	31%	38%	38%	45%	47%	29%	30%	33%	36%	32%

f24.0125 Sh/Kr/Ul, May 2024

	Age																	
	2019 / 18-39 years	2020 / 18-39 years	2021 / 18-39 years	2022 / 18-39 years	2023 / 18-39 years	2024 / 18-39 years	2019 / 40-59 years	2020 / 40-59 years	2021 / 40-59 years	2022 / 40-59 years	2023 / 40-59 years	2024 / 40-59 years	2019 / 60 years and older	2020 / 60 years and older	2021 / 60 years and older	2022 / 60 y. and older	2023 / 60 y. and older	2024 / 60 years and older

Question 1: Not applicable

Question 2: 'Patient safety' is the successful endeavour to ensure error-free and harm-free medical treatment and medical healthcare. In your opinion, how likely is it that patients in Germany will come to harm as a result of medical treatment in hospital? Do you think this is 'very likely' - 'fairly likely' - 'not very likely' - or 'unlikely'?

	2019 / 18-39 years	2020 / 18-39 years	2021 / 18-39 years	2022 / 18-39 years	2023 / 18-39 years	2024 / 18-39 years	2019 / 40-59 years	2020 / 40-59 years	2021 / 40-59 years	2022 / 40-59 years	2023 / 40-59 years	2024 / 40-59 years	2019 / 60 years and older	2020 / 60 years and older	2021 / 60 years and older	2022 / 60 y. and older	2023 / 60 y. and older	2024 / 60 years and older
Basis (100%)	319	318	318	316	315	317	348	348	340	337	332	328	333	334	342	347	352	355
very likely	7%	3%	8%	9%	7%	6%	13%	12%	8%	8%	10%	10%	10%	9%	8%	15%	12%	11%
Fairly likely	30%	24%	14%	17%	19%	15%	41%	24%	26%	25%	21%	28%	33%	23%	18%	19%	23%	22%
not very likely	49%	56%	54%	53%	55%	59%	41%	58%	53%	62%	62%	51%	49%	56%	57%	59%	56%	56%
unlikely	13%	16%	24%	21%	19%	19%	3%	6%	14%	5%	7%	11%	6%	9%	17%	8%	8%	10%
wn/kA	-		-		-	-	2%		-		1%		1%	2%			1%	
Sum	100%	100%	100%	100%	100%	100%	100%	100%	100%	100%	100%	100%	100%	100%	100%	100%	100%	100%
very/somewhat likely	38%	27%	22%	25%	26%	22%	54%	37%	34%	33%	31%	37%	43%	32%	25%	33%	35%	34%
not very likely/unlikely	62%	73%	78%	74%	74%	78%	44%	63%	66%	67%	69%	62%	56%	65%	74%	67%	64%	66%

Question 3: And in your opinion, how likely is it that
that patients are harmed by medical care outside of a hospital in

Germany, e.g. through outpatient treatment by a doctor or incorrect medication? Do you think this is 'very likely' - 'fairly likely' - 'not very likely'?
'fairly likely' - 'not very likely' -
or 'unlikely'?

	2019 / 18-39 years	2020 / 18-39 years	2021 / 18-39 years	2022 / 18-39 years	2023 / 18-39 years	2024 / 18-39 years	2019 / 40-59 years	2020 / 40-59 years	2021 / 40-59 years	2022 / 40-59 years	2023 / 40-59 years	2024 / 40-59 years	2019 / 60 years and older	2020 / 60 years and older	2021 / 60 years and older	2022 / 60 y. and older	2023 / 60 y. and older	2024 / 60 years and older
Basis (100%)	319	318	318	316	315	317	348	348	340	337	332	328	333	334	342	347	352	355
very likely	7%	3%	9%	8%	6%	6%	10%	11%	14%	6%	6%	6%	11%	6%	9%	7%	8%	6%
Fairly likely	29%	24%	22%	21%	25%	19%	34%	19%	24%	27%	23%	28%	27%	30%	18%	27%	25%	25%
not very likely	54%	57%	56%	59%	56%	62%	49%	62%	52%	61%	65%	57%	57%	56%	63%	61%	55%	59%
unlikely	10%	15%	13%	11%	12%	13%	6%	7%	10%	5%	6%	7%	6%	7%	7%	5%	9%	8%
wn/kA					1%	-					1%	1%		1%	3%		2%	2%
Total	100%	100%	100%	100%	100%	100%	100%	100%	100%	100%	100%	100%	100%	100%	100%	100%	100%	100%
very/somewhat likely	35%	27%	31%	29%	31%	26%	44%	30%	38%	33%	29%	34%	37%	36%	27%	34%	34%	31%
not very likely/unlikely	64%	72%	69%	70%	68%	74%	56%	69%	62%	67%	71%	65%	63%	63%	70%	66%	64%	67%

Question 4: I will now tell you about possible harmful incidents in medical care, which are also called
called 'adverse events'.

Please tell me in each case whether you think this will happen to you: 'yes, definitely' - 'yes, probably' - 'probably not' - or 'definitely not'? How likely is it that the following will happen to you: ...?

Overview table: yes, definitely/probably

Age	2019 / 18-39 years	2020 / 18-39 years	2021 / 18-39 years	2022 / 18-39 years	2023 / 18-39 years	2024 / 18-39 years	2019 / 40-59 years	2020 / 40-59 years	2021 / 40-59 years	2022 / 40-59 years	2023 / 40-59 years	2024 / 40-59 years	2019 / 60 years and older	2020 / 60 years and older	2021 / 60 years and older	2022 / 60 y. and older	2023 / 60 y. and older	2024 / 60 Y. and older
Basis (100%)	319	318	318	316	315	317	348	348	340	337	332	328	333	334	342	347	352	355
Infection with dangerous germs in hospital	51%	47%	52%	47%	45%	46%	66%	61%	66%	69%	56%	66%	71%	59%	66%	63%	68%	68%
an incorrect diagnosis	65%	58%	59%	53%	74%	60%	61%	50%	57%	56%	56%	54%	52%	44%	43%	41%	53%	48%
an error during an operation, e.g. a surgical error surgical error	33%	26%	27%	26%	22%	25%	45%	37%	41%	28%	31%	26%	47%	30%	35%	21%	32%	35%
an error with medicines due to incorrect prescription, incorrect dosage or incorrect method of use	52%	38%	48%	39%	45%	36%	54%	41%	43%	36%	48%	38%	39%	35%	41%	25%	35%	32%
an error caused by a medical treatment device	25%	18%	25%	19%	19%	13%	35%	22%	28%	16%	20%	17%	30%	27%	20%	16%	27%	22%

f24.0125 Sh/Kr/Ul, May 2024

Question 4: I will now tell you about possible harmful events in medical care, which are also called
called 'adverse events'.

Please tell me in each case whether you think this will happen to you: 'yes, definitely' - 'yes, probably' - 'probably not' - or 'definitely not'? How likely is it that the following will happen to you: ...?

... an infection with dangerous germs in hospital

Age	2019 / 18-39 years	2020 / 18-39 years	2021 / 18-39 years	2022 / 18-39 years	2023 / 18-39 years	2024 / 18-39 years	2019 / 40-59 years	2020 / 40-59 years	2021 / 40-59 years	2022 / 40-59 years	2023 / 40-59 years	2024 / 40-59 years	2019 / 60 years and older	2020 / 60 years and older	2021 / 60 years and older	2022 / 60 y. and older	2023 / 60 y. and older	2024 / 60 Y. and older
Basis (100%)	319	318	318	316	315	317	348	348	340	337	332	328	333	334	342	347	352	355
yes, definitely (incl. has already happened to me)	8%	5%	10%	6%	9%	8%	19%	18%	17%	14%	11%	18%	22%	12%	17%	9%	16%	15%
yes, probably	43%	42%	42%	41%	37%	38%	47%	43%	50%	55%	45%	48%	50%	47%	49%	55%	52%	53%
probably not	38%	46%	39%	45%	46%	49%	31%	30%	29%	28%	39%	30%	24%	30%	31%	33%	28%	26%
definitely not	10%	6%	8%	8%	8%	5%	3%	8%	2%	3%	4%	4%	4%	8%	1%	2%	4%	6%
wn/kA	2%	-		-	1%	-	-	1%	2%	-	-			3%	2%	2%		
total	100%	100%	100%	100%	100%	100%	100%	100%	100%	100%	100%	100%	100%	100%	100%	100%	100%	100%
yes, definitely/probably	51%	47%	52%	47%	45%	46%	66%	61%	66%	69%	56%	66%	71%	59%	66%	63%	68%	68%
definitely not/probably not	48%	53%	47%	53%	54%	54%	34%	38%	31%	31%	44%	34%	28%	38%	32%	35%	32%	32%

Question 4: I will now tell you about possible harmful events in medical care, which are also called
called 'adverse events'.

Please tell me in each case whether you think this will happen to you: 'yes, definitely' - 'yes, probably' - 'probably not' - or 'definitely not'? How likely is it that the following will happen to you: ...?

... an incorrect diagnosis

Basis (100%)	319	318	318	316	315	317	348	348	340	337	332	328	333	334	342	347	352	355
yes, definitely (incl. has already happened to me)	19%	17%	18%	16%	24%	15%	12%	15%	15%	9%	17%	9%	13%	11%	7%	8%	11%	15%
yes, probably	46%	41%	41%	37%	50%	45%	49%	35%	42%	47%	39%	45%	39%	33%	36%	33%	42%	34%
probably not	28%	37%	37%	39%	23%	35%	36%	46%	42%	41%	37%	39%	42%	44%	45%	54%	39%	39%
definitely not	6%	5%	3%	8%	3%	5%	3%	4%	1%	2%	6%	7%	6%	10%	12%	4%	8%	11%
wn/kA		-	-	-	-	-	-	1%				1%		2%		-		1%
total	100%	100%	100%	100%	100%	100%	100%	100%	100%	100%	100%	100%	100%	100%	100%	100%	100%	100%
yes, definitely/probably	65%	58%	59%	53%	74%	60%	61%	50%	57%	56%	56%	54%	52%	44%	43%	41%	53%	48%
definitely not/probably not	34%	42%	41%	47%	26%	40%	39%	50%	43%	44%	43%	45%	48%	54%	57%	59%	47%	51%

Question 4: I will now tell you about possible harmful occurrences in medical care, which are also called
called 'adverse events'.

Please tell me in each case whether you think this will happen to you: 'yes, definitely' - 'yes, probably' - 'probably not' - or 'definitely not'? How likely is it that the following will happen to you: ...?

... an error during an operation, e.g. a surgical error

Basis (100%)	319	318	318	316	315	317	348	348	340	337	332	328	333	334	342	347	352	355
yes, definitely (incl. has already happened to me)	6%	7%	6%	8%	4%	5%	7%	8%	10%	4%	7%	6%	5%	4%	8%	5%	5%	4%
yes, probably	27%	19%	22%	18%	18%	20%	38%	28%	31%	24%	24%	20%	41%	26%	27%	17%	27%	32%
probably not	60%	60%	60%	62%	59%	67%	51%	50%	53%	65%	61%	65%	43%	56%	53%	68%	58%	55%
definitely not	8%	14%	13%	12%	19%	8%	4%	13%	5%	8%	8%	8%	9%	12%	10%	10%	10%	10%

f24.0125 Sh/Kr/Ul, May 2024	Age																	
	2019 / 18-39 years	2020 / 18-39 years	2021 / 18-39 years	2022 / 18-39 years	2023 / 18-39 years	2024 / 18-39 years	2019 / 40-59 years	2020 / 40-59 years	2021 / 40-59 years	2022 / 40-59 years	2023 / 40-59 years	2024 / 40-59 years	2019 / 60 years and older	2020 / 60 years and older	2021 / 60 years and older	2022 / 60 years and older	2023 / 60 y. and older	2024 / 60 y. and older
wn/kA			-	-	-	-		1%	1%	-			1%	2%	2%	1%		
total	100%	100%	100%	100%	100%	100%	100%	100%	100%	100%	100%	100%	100%	100%	100%	100%	100%	100%
yes, definitely/probably	33%	26%	27%	26%	22%	25%	45%	37%	41%	28%	31%	26%	47%	30%	35%	21%	32%	35%
definitely not/probably not	67%	74%	73%	74%	78%	75%	55%	63%	58%	72%	68%	73%	52%	68%	63%	78%	68%	64%

Question 4: I will now tell you about possible harmful events in medical care, which are also called
called 'adverse events'.

Please tell me in each case whether you think this will happen to you: 'yes, definitely' - 'yes, probably' - 'probably not' - or 'definitely not'?
How likely is it that the following will happen to you: ...?

... an error with medicines due to incorrect prescription, incorrect dosage or incorrect method of use	2019 / 18-39 years	2020 / 18-39 years	2021 / 18-39 years	2022 / 18-39 years	2023 / 18-39 years	2024 / 18-39 years	2019 / 40-59 years	2020 / 40-59 years	2021 / 40-59 years	2022 / 40-59 years	2023 / 40-59 years	2024 / 40-59 years	2019 / 60 years and older	2020 / 60 years and older	2021 / 60 years and older	2022 / 60 years and older	2023 / 60 y. and older	2024 / 60 y. and older
Basis (100%)	319	318	318	316	315	317	348	348	340	337	332	328	333	334	342	347	352	355
yes, definitely (incl. has already happened to me)	8%	5%	11%	10%	13%	6%	9%	11%	12%	7%	10%	8%	8%	6%	13%	5%	7%	7%
yes, probably	44%	32%	38%	28%	32%	29%	45%	30%	32%	29%	38%	30%	32%	29%	28%	20%	28%	26%
probably not	40%	52%	43%	50%	43%	53%	40%	46%	50%	54%	44%	53%	49%	47%	43%	59%	51%	53%
definitely not	8%	11%	9%	11%	11%	12%	6%	13%	6%	10%	8%	10%	12%	17%	16%	16%	11%	15%
wn/kA		-	-	-	1%	-	-		-		-			1%		-	3%	
total	100%	100%	100%	100%	100%	100%	100%	100%	100%	100%	100%	100%	100%	100%	100%	100%	100%	100%
yes, definitely/probably	52%	38%	48%	39%	45%	36%	54%	41%	43%	36%	48%	38%	39%	35%	41%	25%	35%	32%
definitely not/probably not	48%	62%	52%	61%	54%	64%	46%	59%	57%	64%	52%	62%	61%	64%	59%	75%	62%	67%

Question 4: I will now tell you about possible harmful events in medical care, which are also called
called 'adverse events'.

Please tell me in each case whether you think this will happen to you: 'yes, definitely' - 'yes, probably' - 'probably not' - or 'definitely not'?
How likely is it that the following will happen to you: ...?

... an error caused by a medical treatment device																		
Basis (100%)	319	318	318	316	315	317	348	348	340	337	332	328	333	334	342	347	352	355
yes, definitely (incl. has already happened to me)	5%	4%	3%	2%	2%	3%	5%	3%	4%	1%	6%	2%	4%	1%	4%	1%	2%	3%
yes, probably	20%	14%	22%	17%	17%	10%	30%	19%	24%	16%	14%	15%	27%	25%	17%	15%	25%	19%
probably not	61%	64%	61%	55%	66%	69%	57%	63%	61%	68%	65%	69%	56%	60%	64%	72%	60%	61%
definitely not	14%	18%	14%	26%	16%	18%	9%	14%	9%	15%	14%	14%	13%	11%	12%	12%	12%	16%
wn/kA	-	-		-	-	-		1%	1%		1%		1%	3%	4%		1%	1%
Sum	100%	100%	100%	100%	100%	100%	100%	100%	100%	100%	100%	100%	100%	100%	100%	100%	100%	100%
yes, definitely/probably	25%	18%	25%	19%	19%	13%	35%	22%	28%	16%	20%	17%	30%	27%	20%	16%	27%	22%
definitely not/probably not	75%	82%	75%	81%	81%	87%	65%	77%	70%	84%	79%	83%	69%	71%	76%	84%	72%	77%

Question 5: I will now read these 'adverse events' to you again. Please tell me in each case whether you think that this can be largely avoided in future by 'suitable measures': 'yes, definitely' - 'yes, probably' - 'probably not' - or 'definitely not'? Can the following be largely avoided in future ... ?

Overview table: yes, definitely/probably																		
Basis (100%)	319	318	318	316	315	317	348	348	340	337	332	328	333	334	342	347	352	355
Infection with dangerous germs in hospital	61%	59%	61%	51%	65%	57%	62%	59%	67%	51%	54%	47%	68%	63%	72%	55%	67%	64%
an incorrect diagnosis	62%	67%	64%	56%	61%	65%	59%	52%	59%	55%	58%	53%	56%	58%	59%	47%	49%	58%

f24.0125 Sh/Kr/Ul, May 2024

	Age																	
	2019 / 18-39 years	2020 / 18-39 years	2021 / 18-39 years	2022 / 18-39 years	2023 / 18-39 years	2024 / 18-39 years	2019 / 40-59 years	2020 / 40-59 years	2021 / 40-59 years	2022 / 40-59 years	2023 / 40-59 years	2024 / 40-59 years	2019 / 60 years and older	2020 / 60 years and older	2021 / 60 years and older	2022 / 60 years and older	2023 / 60 y. and older	2024 / 60 Y. and older
an error during an operation, e.g. a surgical error	55%	60%	64%	64%	67%	63%	56%	62%	57%	55%	61%	60%	54%	56%	53%	46%	53%	52%
an error with medicines due to incorrect prescription, wrong dosage or wrong type of application	66%	64%	70%	67%	66%	71%	66%	53%	61%	65%	60%	58%	55%	50%	62%	47%	53%	61%
an error caused by a medical treatment device	68%	70%	76%	74%	73%	77%	64%	60%	64%	67%	64%	65%	46%	51%	54%	47%	49%	50%

Question 5: I will now read these 'adverse events' to you again. Please tell me now in each case whether you think that this can be largely avoided in future by
'suitable measures': 'yes, definitely' - 'yes, probably' - 'probably not' - or 'definitely not'? Can the following be largely avoided in future ... ?

... infection with dangerous germs in hospital

	2019 / 18-39 years	2020 / 18-39 years	2021 / 18-39 years	2022 / 18-39 years	2023 / 18-39 years	2024 / 18-39 years	2019 / 40-59 years	2020 / 40-59 years	2021 / 40-59 years	2022 / 40-59 years	2023 / 40-59 years	2024 / 40-59 years	2019 / 60 years and older	2020 / 60 years and older	2021 / 60 years and older	2022 / 60 years and older	2023 / 60 y. and older	2024 / 60 Y. and older
Basis (100%)	319	318	318	316	315	317	348	348	340	337	332	328	333	334	342	347	352	355
yes, definitely	18%	17%	14%	14%	22%	18%	23%	11%	14%	15%	14%	10%	21%	12%	12%	11%	14%	15%
yes, probably	43%	42%	48%	37%	43%	39%	39%	49%	53%	36%	40%	37%	47%	50%	60%	44%	52%	49%
probably not	27%	36%	32%	42%	31%	37%	31%	34%	27%	38%	37%	41%	28%	30%	24%	34%	30%	28%
definitely not	13%	5%	7%	8%	4%	6%	6%	7%	6%	11%	9%	12%	4%	7%	5%	10%	4%	7%
wn/kA		-	-	-	-	-	1%	-	-	-	-	-	-	-	-	-	-	-
Total	100%	100%	100%	100%	100%	100%	100%	100%	100%	100%	100%	100%	100%	100%	100%	100%	100%	100%
yes, definitely/probably	61%	59%	61%	51%	65%	57%	62%	59%	67%	51%	54%	47%	68%	63%	72%	55%	67%	64%
definitely not/probably not	39%	41%	39%	49%	35%	43%	37%	41%	33%	49%	46%	53%	32%	37%	28%	45%	33%	36%

Question 5: I will now read these 'adverse events' to you again. Please tell me in each case whether you think that this can be largely avoided in future by
'suitable measures': 'yes, definitely' - 'yes, probably' - 'probably not' - or 'definitely not'? Can the following be largely avoided in future ... ?

... an incorrect diagnosis

Basis (100%)	319	318	318	316	315	317	348	348	340	337	332	328	333	334	342	347	352	355
yes, definitely	21%	20%	20%	17%	24%	18%	16%	12%	11%	18%	14%	14%	14%	13%	16%	8%	8%	15%
yes, probably	41%	47%	44%	39%	38%	47%	43%	40%	48%	37%	44%	39%	41%	45%	42%	39%	41%	43%
probably not	31%	27%	29%	33%	32%	30%	36%	40%	33%	37%	36%	39%	34%	35%	37%	47%	44%	35%
definitely not	6%	6%	6%	11%	6%	4%	4%	7%	8%	8%	5%	8%	9%	6%	3%	6%	6%	7%
wn/kA			1%	-		-		1%	1%	-			2%	1%	1%	1%	1%	
Sum	100%	100%	100%	100%	100%	100%	100%	100%	100%	100%	100%	100%	100%	100%	100%	100%	100%	100%
yes, definitely/probably	62%	67%	64%	56%	61%	65%	59%	52%	59%	55%	58%	53%	56%	58%	59%	47%	49%	58%
definitely not/probably not	38%	33%	35%	44%	38%	35%	40%	48%	41%	45%	41%	46%	43%	41%	41%	53%	50%	41%

Question 5: I will now read these 'adverse events' to you again. Please tell me in each case whether you think that this can be largely avoided in future by 'suitable measures': 'yes, definitely' - 'yes, probably' - 'probably not' - or 'definitely not'? Can the following be largely avoided in future ... ?

... an error during an operation, e.g. a surgical error

Basis (100%)	319	318	318	316	315	317	348	348	340	337	332	328	333	334	342	347	352	355
yes, definitely	20%	12%	22%	18%	25%	23%	15%	16%	11%	17%	14%	16%	15%	14%	11%	8%	8%	12%
yes, probably	36%	48%	42%	46%	42%	39%	40%	46%	46%	38%	47%	44%	40%	42%	42%	38%	45%	41%

f24.0125 Sh/Kr/Ul, May 2024

	Age																	
	2019 / 18-39 years	2020 / 18-39 years	2021 / 18-39 years	2022 / 18-39 years	2023 / 18-39 years	2024 / 18-39 years	2019 / 40-59 years	2020 / 40-59 years	2021 / 40-59 years	2022 / 40-59 years	2023 / 40-59 years	2024 / 40-59 years	2019 / 60 years and older	2020 / 60 years and older	2021 / 60 years and older	2022 / 60 years and older	2023 / 60 y. and older	2024 / 60 y. and older
probably not	33%	32%	28%	27%	27%	32%	41%	30%	35%	36%	32%	30%	36%	31%	37%	45%	42%	39%
definitely not	12%	7%	8%	9%	6%	5%	3%	7%	7%	9%	6%	10%	8%	10%	9%	7%	4%	7%
wn/kA		1%	1%	-	-	-		1%	1%				2%	3%	1%	2%		1%
Total	100%	100%	100%	100%	100%	100%	100%	100%	100%	100%	100%	100%	100%	100%	100%	100%	100%	100%
yes, definitely/probably	55%	60%	64%	64%	67%	63%	56%	62%	57%	55%	61%	60%	54%	56%	53%	46%	53%	52%
definitely not/probably not	44%	39%	35%	36%	33%	37%	44%	37%	42%	45%	39%	40%	44%	41%	47%	52%	46%	46%

Question 5: I will now read these 'adverse events' to you again. Please tell me in each case whether you think that this can be largely avoided in future by 'suitable measures': 'yes, definitely' - 'yes, probably' - 'probably not' - or 'definitely not'? Can the following be largely avoided in future ... ?

... an error with medicines due to incorrect prescription, incorrect dosage or incorrect method of use

	2019 / 18-39 years	2020 / 18-39 years	2021 / 18-39 years	2022 / 18-39 years	2023 / 18-39 years	2024 / 18-39 years	2019 / 40-59 years	2020 / 40-59 years	2021 / 40-59 years	2022 / 40-59 years	2023 / 40-59 years	2024 / 40-59 years	2019 / 60 years and older	2020 / 60 years and older	2021 / 60 years and older	2022 / 60 years and older	2023 / 60 y. and older	2024 / 60 y. and older
Basis (100%)	319	318	318	316	315	317	348	348	340	337	332	328	333	334	342	347	352	355
yes, definitely	23%	24%	25%	22%	27%	30%	23%	18%	16%	22%	15%	17%	15%	15%	16%	13%	13%	19%
yes, probably	43%	40%	45%	44%	39%	41%	42%	35%	45%	43%	45%	42%	40%	35%	46%	34%	40%	42%
probably not	28%	31%	24%	23%	32%	26%	29%	39%	30%	28%	35%	34%	38%	36%	32%	45%	41%	31%
definitely not	6%	5%	6%	10%	2%	3%	5%	8%	6%	7%	5%	8%	6%	12%	6%	8%	5%	7%
wn/kA	1%			-	-	-	-		3%	-			1%	2%	1%	-		2%
total	100%	100%	100%	100%	100%	100%	100%	100%	100%	100%	100%	100%	100%	100%	100%	100%	100%	100%
yes, definitely/probably	66%	64%	70%	67%	66%	71%	66%	53%	61%	65%	60%	58%	55%	50%	62%	47%	53%	61%
definitely not/probably not	33%	36%	30%	33%	34%	29%	34%	47%	36%	35%	40%	42%	44%	48%	38%	53%	47%	37%

Question 5: I will now read these 'adverse events' to you again. Please tell me now in each case whether you think that this can be largely avoided in future by
'suitable measures': 'yes, definitely' - 'yes, probably' - 'probably not' - or 'definitely not'? Can the following be largely avoided in future ... ?

... an error caused by a medical treatment device

Basis (100%)	319	318	318	316	315	317	348	348	340	337	332	328	333	334	342	347	352	355
yes, definitely	20%	27%	26%	27%	29%	26%	23%	18%	16%	20%	21%	25%	10%	16%	11%	8%	12%	14%
yes, probably	48%	43%	50%	47%	44%	51%	41%	42%	47%	47%	43%	39%	36%	35%	43%	39%	37%	36%
probably not	22%	26%	19%	19%	23%	17%	28%	27%	31%	22%	29%	27%	44%	37%	33%	46%	43%	41%
definitely not	9%	4%	4%	7%	4%	6%	5%	11%	4%	11%	5%	7%	8%	8%	10%	6%	7%	6%
wn/kA					-	-	3%	2%	1%		2%	1%	2%	4%	4%	1%	1%	2%
Sum	100%	100%	100%	100%	100%	100%	100%	100%	100%	100%	100%	100%	100%	100%	100%	100%	100%	100%
yes, definitely/probably	68%	70%	76%	74%	73%	77%	64%	60%	64%	67%	64%	65%	46%	51%	54%	47%	49%	50%
definitely not/probably not	32%	30%	23%	26%	27%	23%	33%	38%	35%	33%	34%	35%	52%	45%	42%	52%	50%	48%

Question 6 and question 7: Not applicable

Question 8: We have now talked a little about the topic of 'patient safety'. How well informed do you feel about patient safety in general: 'very well' - 'well' - 'less well' - or 'not informed at all'?
'well' - 'less well' - or 'not informed at all'?

Basis (100%)	319	318	318	316	315	317	348	348	340	337	332	328	333	334	342	347	352	355
Very good	11%	9%	16%	15%	11%	8%	7%	12%	17%	13%	11%	8%	9%	12%	20%	14%	13%	18%
good	34%	48%	46%	43%	40%	44%	43%	52%	45%	49%	51%	51%	60%	71%	62%	58%	61%	52%

	2019 / 18-39 years	2020 / 18-39 years	2021 / 18-39 years	2022 / 18-39 years	2023 / 18-39 years	2024 / 18-39 years	2019 / 40-59 years	2020 / 40-59 years	2021 / 40-59 years	2022 / 40-59 years	2023 / 40-59 years	2024 / 40-59 years	2019 / 60 years and older	2020 / 60 years and older	2021 / 60 years and older	2022 / 60 years and older	2023 / 60 y. and older	2024 / 60 years and older
less good	39%	31%	31%	31%	43%	38%	39%	28%	27%	26%	27%	30%	25%	15%	12%	22%	21%	23%
Not informed at all	16%	13%	7%	10%	7%	10%	11%	8%	11%	11%	9%	10%	5%	3%	4%	6%	5%	6%
wn/kA		-	-	-	-				-			1%	-		2%			1%

TK Patient Safety Monitor: Time comparison 2019 to 2024

f24.0125 Sh/Kr/Ul, May 2024	Age																	
	2019 / 18-39 years	2020 / 18-39 years	2021 / 18-39 years	2022 / 18-39 years	2023 / 18-39 years	2024 / 18-39 years	2019 / 40-59 years	2020 / 40-59 years	2021 / 40-59 years	2022 / 40-59 years	2023 / 40-59 years	2024 / 40-59 years	2019 / 60 years and older	2020 / 60 years and older	2021 / 60 years and older	2022 / 60 years and older	2023 / 60 y. and older	2024 / 60 years and older
Total	100%	100%	100%	100%	100%	100%	100%	100%	100%	100%	100%	100%	100%	100%	100%	100%	100%	100%
(very) good	45%	56%	62%	59%	51%	52%	50%	64%	62%	63%	63%	59%	70%	82%	82%	71%	74%	70%
less good/not at all	55%	44%	38%	41%	49%	47%	49%	36%	38%	37%	37%	40%	30%	18%	16%	28%	26%	29%

Question 9: All in all, what do you think: As a patient, can you yourself contribute to ensuring that you receive safe care at the doctor's surgery or hospital?
'yes, definitely' - 'rather yes' - 'rather not' - or
can you as a patient 'not at all' contribute to this?

	2019 / 18-39 years	2020 / 18-39 years	2021 / 18-39 years	2022 / 18-39 years	2023 / 18-39 years	2024 / 18-39 years	2019 / 40-59 years	2020 / 40-59 years	2021 / 40-59 years	2022 / 40-59 years	2023 / 40-59 years	2024 / 40-59 years	2019 / 60 years and older	2020 / 60 years and older	2021 / 60 years and older	2022 / 60 years and older	2023 / 60 y. and older	2024 / 60 years and older
Basis (100%)	319	318	318	316	315	317	348	348	340	337	332	328	333	334	342	347	352	355
yes, definitely	23%	31%	26%	29%	26%	26%	21%	37%	35%	31%	25%	24%	36%	35%	41%	29%	37%	34%
rather yes	42%	40%	47%	45%	38%	38%	46%	36%	40%	40%	46%	43%	41%	47%	34%	46%	40%	37%
rather not	24%	24%	23%	23%	27%	31%	28%	22%	19%	24%	25%	21%	16%	14%	13%	20%	19%	24%
not at all	9%	5%	4%	3%	8%	5%	5%	6%	6%	6%	4%	10%	5%	4%	12%	4%	5%	5%
wn/kA	2%		-				1%	-		-		1%	2%			1%	-	1%
total	100%	100%	100%	100%	100%	100%	100%	100%	100%	100%	100%	100%	100%	100%	100%	100%	100%	100%
definitely/rather yes	64%	71%	73%	74%	64%	64%	67%	72%	74%	70%	71%	68%	77%	82%	75%	75%	77%	71%
rather not/not at all	33%	28%	27%	25%	35%	36%	32%	28%	25%	30%	29%	31%	21%	18%	25%	24%	23%	28%

f24.0125 Sh/Kr/Ul, May 2024

	State of health											
	2019 / very good/good	2020 / very good/good	2021 / very good/good	2022 / very good/good	2023 / very good/good	2024 / very good/good	2019 / at-peace fair/low less good/poor bad	2020 / at-peace good/low good/bad good	2021 / too-peaceful good/low good/bad good	2022 / too-peaceful-good/bad good/bad good	2023 / too-peaceful-good/bad good/bad good	2024 / too-peaceful-good/bad good/bad good
Question 1: Not applicable												
Question 2: 'Patient safety' is the successful endeavour to ensure error-free and harm-free medical treatment and medical healthcare. In your opinion, how likely is it that patients in Germany will come to harm as a result of medical treatment in hospital? Do you think this is 'very likely' - 'fairly likely' - 'not very likely' - or 'unlikely'?												
Basis (100%)	572	637	626	589	578	608	428	363	374	411	422	393
very likely	8%	8%	6%	7%	8%	7%	12%	10%	11%	16%	12%	12%
Fairly likely	32%	21%	17%	18%	23%	23%	39%	28%	23%	23%	19%	20%
not very likely	50%	60%	56%	63%	56%	55%	42%	51%	52%	51%	59%	57%
unlikely	8%	10%	20%	12%	13%	15%	6%	10%	15%	11%	9%	11%
wn/kA	2%	1%					-	1%	-		1%	1%
Sum	100%	100%	100%	100%	100%	100%	100%	100%	100%	100%	100%	100%
very/somewhat likely	40%	29%	23%	25%	31%	30%	51%	38%	34%	38%	30%	32%
not very likely/unlikely	58%	70%	76%	74%	69%	70%	49%	61%	66%	62%	69%	67%

Question 3: And in your opinion, how likely is it that patients are harmed by medical care outside a hospital in

Germany, e.g. through outpatient treatment by a doctor or the wrong medication? Do you think this is 'very likely' - 'fairly likely' - 'not very likely'?
'fairly likely' - 'not very likely' - or
'unlikely'?

Basis (100%)	572	637	626	589	578	608	428	363	374	411	422	393
very likely	3%	7%	9%	4%	4%	6%	17%	6%	14%	11%	10%	5%
Fairly likely	30%	22%	22%	21%	21%	22%	30%	29%	21%	31%	29%	27%
not very likely	59%	60%	56%	67%	63%	60%	46%	56%	58%	51%	52%	58%
unlikely	8%	11%	13%	7%	11%	10%	7%	8%	4%	7%	7%	8%
wn/kA					1%	1%		1%	2%		2%	1%
Total	100%	100%	100%	100%	100%	100%	100%	100%	100%	100%	100%	100%
very/somewhat likely	33%	29%	30%	25%	26%	29%	47%	35%	35%	42%	39%	33%
not very likely/unlikely	66%	71%	69%	74%	73%	70%	53%	64%	63%	58%	59%	66%

f24.0125 Sh/Kr/Ul, May 2024

	State of health											
	2019 / very good/go od	2020 / very good/go od	2021 / very good/go od	2022 / very good/go od	2023 / very good/go od	2024 / very good/go od	2019 / at-peace goo d/low less good/poor bad	2020 / at-peace goo d/low go od/bad good	2021 / too-peaceful goo d/low go od/bad good	2022 / too-peaceful-goo d/bad go od/bad good	2023 / too-peaceful-goo d/bad go od/bad good	2024 / too-peaceful-goo d/bad go od/bad good
Question 4: I will now tell you about possible harmful incidents in medical care, which are also called called 'adverse events'. Please tell me in each case whether you think this will happen to you: 'yes, definitely' - 'yes, probably' - 'probably not' - or 'definitely not'? How likely is it that the following will happen to you: ...?												
Overview table: yes, definitely/probably												
Basis (100%)	572	637	626	589	578	608	428	363	374	411	422	393
Infection with dangerous germs in hospital	58%	53%	60%	56%	54%	57%	69%	62%	65%	66%	61%	65%
an incorrect diagnosis	54%	48%	50%	44%	57%	51%	66%	55%	58%	59%	66%	58%
an error during an operation, e.g. a surgical error	31%	31%	32%	21%	23%	28%	56%	31%	39%	31%	37%	31%
an error with medicines due to incorrect prescription, incorrect dosage or incorrect method of use	46%	36%	40%	27%	36%	34%	51%	42%	51%	41%	51%	37%
an error caused by a medical treatment device	23%	21%	21%	13%	16%	14%	40%	24%	30%	22%	31%	23%

Question 4: I will now tell you about possible harmful events in medical care, which are also called
called 'adverse events'.

Please tell me in each case whether you think this will happen to you: 'yes, definitely' - 'yes, probably' - 'probably not' - or 'definitely not'? How likely is it that the following will happen to you: ...?

... an infection with dangerous germs in hospital

Basis (100%)	572	637	626	589	578	608	428	363	374	411	422	393
yes, definitely (incl. has already happened to me)	13%	13%	10%	8%	12%	10%	20%	10%	23%	11%	12%	19%
yes, probably	45%	40%	50%	48%	42%	47%	49%	52%	42%	54%	49%	46%
probably not	35%	38%	35%	38%	40%	37%	25%	30%	29%	32%	34%	31%
definitely not	6%	9%	4%	5%	6%	6%	6%	6%	2%	3%	4%	4%
wn/kA	1%	1%		1%	-			2%	4%	-	1%	
total	100%	100%	100%	100%	100%	100%	100%	100%	100%	100%	100%	100%
yes, definitely/probably	58%	53%	60%	56%	54%	57%	69%	62%	65%	66%	61%	65%
definitely not/probably not	41%	47%	40%	43%	46%	43%	30%	36%	31%	34%	39%	35%

f24.0125 Sh/Kr/Ul, May 2024

Question 4: I will now tell you about possible harmful incidents in medical care, which are also called
called 'adverse events'.

Please tell me in each case whether you think this will happen to you: 'yes, definitely' - 'yes, probably' - 'probably not' - or 'definitely not'? How likely is it that the following will happen to you: ...?

... an incorrect diagnosis

	State of health											
	2019 / very good/good	2020 / very good/good	2021 / very good/good	2022 / very good/good	2023 / very good/good	2024 / very good/good	2019 / at-peace fair/low less good/poor bad	2020 / at-peace good/low good/bad good	2021 / too-peaceful good/low good/bad good	2022 / too-peaceful-good/low good/bad good	2023 / too-peaceful-good/bad good/bad good	2024 / too-peaceful-good/bad good/bad good
Basis (100%)	572	637	626	589	578	608	428	363	374	411	422	393
yes, definitely (incl. has already happened to me)	10%	12%	9%	6%	15%	11%	21%	19%	20%	18%	21%	16%
yes, probably	45%	36%	41%	37%	42%	40%	45%	36%	38%	42%	45%	42%
probably not	39%	45%	44%	50%	38%	41%	31%	38%	38%	38%	27%	33%
definitely not	6%	6%	6%	6%	5%	7%	3%	6%	4%	3%	7%	8%
wn/kA							-	1%		-	-	1%
Sum	100%	100%	100%	100%	100%	100%	100%	100%	100%	100%	100%	100%
yes, definitely/probably	54%	48%	50%	44%	57%	51%	66%	55%	58%	59%	66%	58%
definitely not/probably not	45%	51%	50%	56%	43%	48%	34%	44%	42%	41%	34%	41%

Question 4: I will now tell you about possible harmful occurrences in medical care, which are also called
called 'adverse events'.

Please tell me in each case whether you think this will happen to you: 'yes, definitely' - 'yes, probably' - 'probably not' - or 'definitely not'? How likely is it that the following will happen to you: ...?

... an error during an operation, e.g. a surgical error

Basis (100%)	572	637	626	589	578	608	428	363	374	411	422	393
yes, definitely (incl. has already happened to me)	3%	6%	5%	3%	5%	4%	11%	8%	13%	9%	6%	6%
yes, probably	29%	26%	27%	18%	17%	24%	45%	23%	26%	22%	31%	25%
probably not	61%	55%	59%	68%	63%	63%	38%	55%	50%	61%	53%	61%
definitely not	8%	12%	9%	11%	14%	9%	6%	14%	9%	8%	9%	8%
wn/kA	1%	1%							2%			
total	100%	100%	100%	100%	100%	100%	100%	100%	100%	100%	100%	100%
yes, definitely/probably	31%	31%	32%	21%	23%	28%	56%	31%	39%	31%	37%	31%
definitely not/probably not	68%	67%	68%	79%	77%	72%	44%	69%	59%	69%	63%	69%

f24.0125 Sh/Kr/Ul, May 2024

	State of health											
	2019 / very good/good	2020 / very good/good	2021 / very good/good	2022 / very good/good	2023 / very good/good	2024 / very good/good	2019 / at-peace fair/low less good/poor bad	2020 / at-peace good/low good/bad good	2021 / too-peaceful good/low good/bad good	2022 / too-peaceful-good/bad good/bad good	2023 / too-peaceful-good/bad good/bad good	2024 / too-peaceful-good/bad good/bad good
Question 4: I will now tell you about possible harmful incidents in medical care, which are also called called 'adverse events'. Please tell me in each case whether you think this will happen to you: 'yes, definitely' - 'yes, probably' - 'probably not' - or 'definitely not'? How likely is it that the following will happen to you: ...?												
... an error with medicines due to incorrect prescription, incorrect dosage or incorrect method of use												
Basis (100%)	572	637	626	589	578	608	428	363	374	411	422	393
yes, definitely (incl. has already happened to me)	6%	6%	7%	4%	7%	5%	11%	10%	19%	12%	13%	10%
yes, probably	40%	29%	32%	24%	29%	29%	40%	32%	32%	29%	38%	27%
probably not	43%	49%	48%	58%	51%	52%	42%	47%	41%	49%	39%	53%
definitely not	10%	15%	12%	14%	13%	14%	7%	11%	8%	10%	7%	9%
wn/kA						-	-	1%		-	3%	
total	100%	100%	100%	100%	100%	100%	100%	100%	100%	100%	100%	100%
yes, definitely/probably	46%	36%	40%	27%	36%	34%	51%	42%	51%	41%	51%	37%
definitely not/probably not	53%	64%	60%	73%	64%	66%	49%	57%	49%	59%	46%	62%

Question 4: I will now tell you about possible harmful occurrences in medical care, which are also called
called 'adverse events'.

Please tell me in each case whether you think this will happen to you: 'yes, definitely' - 'yes, probably' - 'probably not' - or 'definitely not'? How likely is it that the following will happen to you: ...?

... an error caused by a medical treatment device

Basis (100%)	572	637	626	589	578	608	428	363	374	411	422	393
yes, definitely (incl. has already happened to me)	2%	2%	3%	1%	4%	1%	8%	3%	4%	1%	3%	6%
yes, probably	21%	19%	18%	12%	12%	13%	32%	21%	26%	21%	28%	17%
probably not	65%	62%	67%	66%	70%	69%	49%	63%	54%	65%	55%	63%
definitely not	12%	16%	12%	20%	14%	18%	11%	11%	11%	13%	14%	13%
wn/kA		1%			1%			2%	4%	-		1%
Sum	100%	100%	100%	100%	100%	100%	100%	100%	100%	100%	100%	100%
yes, definitely/probably	23%	21%	21%	13%	16%	14%	40%	24%	30%	22%	31%	23%

f24.0125 Sh/Kr/Ul, May 2024

	State of health											
	2019 / very good/good	2020 / very good/good	2021 / very good/good	2022 / very good/good	2023 / very good/good	2024 / very good/good	2019 / at-peace fair/low less good/poor bad	2020 / at-peace good/low good/bad good	2021 / too-peaceful good/low good/bad good	2022 / too-peaceful-good/bad good/bad good	2023 / too-peaceful-good/bad good/bad good	2024 / too-peaceful-good/bad good/bad good
definitely not/probably not	77%	78%	79%	86%	83%	86%	60%	74%	66%	78%	69%	76%

Question 5: I will now read these 'adverse events' to you again. Please tell me in each case whether you think that this can be largely avoided in future by 'suitable measures': 'yes, definitely' - 'yes, probably' - 'probably not' - or 'definitely not'? Can the following be largely avoided in future ... ?

Overview table: yes, definitely/probably												
Basis (100%)	572	637	626	589	578	608	428	363	374	411	422	393
Infection with dangerous germs in hospital	60%	59%	64%	53%	61%	58%	69%	62%	72%	52%	63%	54%
an incorrect diagnosis	59%	55%	59%	54%	60%	62%	59%	65%	63%	50%	51%	54%
an error during an operation, e.g. a surgical error surgical error	54%	60%	57%	54%	62%	60%	57%	59%	59%	56%	57%	55%
an error with medicines due to incorrect prescription, incorrect dosage or incorrect method of use	63%	54%	62%	60%	58%	65%	61%	57%	68%	58%	61%	61%
an error caused by a medical treatment device	60%	63%	65%	66%	60%	68%	58%	56%	62%	58%	63%	57%

Question 5: I will now read these 'adverse events' to you again. Please tell me now in each case whether you think that this can be largely avoided in future by 'suitable measures': 'yes, definitely' - 'yes, probably' - 'probably not' - or 'definitely not'? Can the following be largely avoided in future ... ?

... infection with dangerous germs in hospital

Basis (100%)	572	637	626	589	578	608	428	363	374	411	422	393
yes, definitely	22%	11%	12%	14%	19%	16%	20%	16%	16%	13%	13%	12%
yes, probably	38%	48%	52%	39%	42%	42%	49%	46%	57%	39%	50%	42%
probably not	32%	34%	30%	38%	35%	35%	24%	31%	22%	38%	30%	36%
definitely not	8%	7%	6%	9%	4%	8%	7%	6%	5%	11%	8%	9%
wn/kA		-	-	-	-	-	1%	-	-	-	-	-
Total	100%	100%	100%	100%	100%	100%	100%	100%	100%	100%	100%	100%
yes, definitely/probably	60%	59%	64%	53%	61%	58%	69%	62%	72%	52%	63%	54%
definitely not/probably not	40%	41%	36%	47%	39%	42%	31%	38%	28%	48%	37%	46%

f24.0125 Sh/Kr/Ul, May 2024

Question 5: I will now read these 'undesirable events' to you again. Please tell me in each case whether you think that this can be largely avoided in future by 'suitable measures': 'yes, definitely' - 'yes, probably' - 'probably not' - or 'definitely not'? Can the following be largely avoided in future ... ?

	State of health											
	2019 / very good/good	2020 / very good/good	2021 / very good/good	2022 / very good/good	2023 / very good/good	2024 / very good/good	2019 / at-peace fair/low less good/poor bad	2020 / at-peace good/low good/bad good	2021 / too-peaceful good/low good/bad good	2022 / too-peaceful-good/bad good/bad good	2023 / too-peaceful-good/bad good/bad good	2024 / too-peaceful-good/bad good/bad good
... an incorrect diagnosis												
Basis (100%)	572	637	626	589	578	608	428	363	374	411	422	393
yes, definitely	16%	14%	13%	17%	17%	17%	19%	16%	20%	10%	12%	13%
yes, probably	43%	41%	46%	37%	43%	45%	40%	49%	43%	40%	39%	41%
probably not	34%	37%	34%	38%	35%	30%	34%	30%	32%	41%	41%	41%
definitely not	6%	7%	6%	8%	4%	7%	7%	4%	5%	8%	8%	5%
wn/kA	1%	1%	1%					1%	1%		1%	
Sum	100%	100%	100%	100%	100%	100%	100%	100%	100%	100%	100%	100%
yes, definitely/probably	59%	55%	59%	54%	60%	62%	59%	65%	63%	50%	51%	54%
definitely not/probably not	40%	44%	40%	46%	40%	38%	41%	34%	37%	49%	48%	46%

Question 5: I will now read these 'adverse events' to you again. Please tell me now in each case whether you think that this can be largely avoided in future by 'suitable measures': 'yes, definitely' - 'yes, probably' - 'probably not' - or 'definitely not'? Can the following be largely avoided in future ... ?

... an error during an operation, e.g. a surgical error

Basis (100%)	572	637	626	589	578	608	428	363	374	411	422	393
yes, definitely	17%	15%	16%	16%	17%	17%	15%	13%	12%	13%	13%	17%
yes, probably	36%	45%	41%	38%	45%	43%	42%	46%	46%	43%	44%	39%
probably not	39%	31%	33%	36%	32%	33%	33%	31%	34%	37%	37%	36%
definitely not	6%	8%	9%	9%	5%	7%	9%	7%	7%	7%	6%	9%
wn/kA	1%	1%	1%	1%		1%	1%	2%				
Total	100%	100%	100%	100%	100%	100%	100%	100%	100%	100%	100%	100%
yes, definitely/probably	54%	60%	57%	54%	62%	60%	57%	59%	59%	56%	57%	55%
definitely not/probably not	45%	39%	42%	45%	37%	39%	42%	38%	41%	44%	42%	44%

f24.0125 Sh/Kr/Ul, May 2024

Question 5: I will now read these 'undesirable events' to you again. Please tell me in each case whether you think that this can be largely avoided in future by 'suitable measures': 'yes, definitely' - 'yes, probably' - 'probably not' - or 'definitely not'? Can the following be largely avoided in future ... ?

... an error with medicines due to incorrect prescription, incorrect dosage or incorrect method of use

	State of health											
	2019 / very good/good	2020 / very good/good	2021 / very good/good	2022 / very good/good	2023 / very good/good	2024 / very good/good	2019 / at-peace fair/low less good/poor bad	2020 / at-peace good/low good/bad good	2021 / too-peaceful good/low good/bad good	2022 / too-peaceful-good/bad good/bad good	2023 / too-peaceful-good/bad good/bad good	2024 / too-peaceful-good/bad good/bad good
Basis (100%)	572	637	626	589	578	608	428	363	374	411	422	393
yes, definitely	21%	17%	17%	21%	20%	22%	20%	22%	22%	16%	15%	22%
yes, probably	42%	37%	45%	39%	38%	44%	41%	36%	45%	42%	45%	39%
probably not	31%	36%	28%	30%	38%	29%	32%	34%	29%	35%	34%	32%
definitely not	5%	9%	8%	9%	4%	5%	6%	7%	3%	7%	5%	7%
wn/kA	1%	1%	2%	-		1%	1%	2%		-		
Total	100%	100%	100%	100%	100%	100%	100%	100%	100%	100%	100%	100%
yes, definitely/probably	63%	54%	62%	60%	58%	65%	61%	57%	68%	58%	61%	61%
definitely not/probably not	36%	45%	37%	40%	41%	34%	38%	41%	32%	42%	39%	39%

Question 5: I will now read these 'adverse events' to you again. Please tell me in each case whether you think that this can be largely avoided in future by 'suitable measures': 'yes, definitely' - 'yes, probably' - 'probably not' - or 'definitely not'? Can the following be largely avoided in future ... ?

... an error caused by a medical treatment device

Basis (100%)	572	637	626	589	578	608	428	363	374	411	422	393
yes, definitely	21%	19%	18%	19%	23%	22%	14%	21%	17%	17%	16%	21%
yes, probably	39%	43%	47%	47%	37%	45%	44%	34%	45%	40%	47%	37%
probably not	32%	27%	26%	27%	33%	25%	31%	35%	31%	33%	30%	36%
definitely not	8%	8%	7%	7%	5%	7%	7%	7%	4%	9%	7%	6%
wn/kA		2%	1%		2%	1%	3%	2%	2%	1%		1%
Sum	100%	100%	100%	100%	100%	100%	100%	100%	100%	100%	100%	100%
yes, definitely/probably	60%	63%	65%	66%	60%	68%	58%	56%	62%	58%	63%	57%
definitely not/probably not	39%	35%	33%	34%	38%	31%	38%	42%	35%	42%	37%	42%

Question 6 and question 7: Not applicable

f24.0125 Sh/Kr/Ul, May 2024

	State of health											
	2019 / very good/go od	2020 / very good/go od	2021 / very good/go od	2022 / very good/go od	2023 / very good/go od	2024 / very good/go od	2019 / at-peace fair/l ow less good/poor bad	2020 / at-peace goo d/low go od/bad good	2021 / too-peaceful goo d/low go od/bad good	2022 / too-peaceful-goo d/bad go od/bad good	2023 / too-peaceful-goo d/bad go od/bad good	2024 / too-peaceful-goo d/bad go od/bad good
Question 8: We have now talked a little about the topic of 'patient safety'. How well informed do you feel about patient safety: 'very well' - 'well' - 'less well' - or 'not informed at all'? 'well' - 'less well' - or 'not informed at all'?												
Basis (100%)	572	637	626	589	578	608	428	363	374	411	422	393
very good	8%	11%	18%	15%	12%	15%	10%	10%	17%	13%	11%	7%
good	46%	58%	52%	52%	51%	47%	45%	54%	49%	48%	51%	52%
less good	35%	25%	23%	26%	28%	31%	34%	24%	22%	27%	32%	28%
Not informed at all	11%	6%	6%	7%	8%	6%	11%	11%	10%	12%	5%	11%
wn/kA	-	-	-						2%			1%
total	100%	100%	100%	100%	100%	100%	100%	100%	100%	100%	100%	100%
(very) good	55%	69%	71%	67%	63%	62%	55%	65%	66%	61%	62%	60%
less good/not at all	45%	31%	29%	33%	37%	38%	44%	35%	32%	39%	38%	40%

f24.0125 Sh/Kr/Ul, May 2024

Question 8: We have now talked a little about the topic of 'patient safety'. How well informed do you feel about patient safety: 'very well' - 'well' - 'less well' - or 'not informed at all'? 'well' - 'less well' - or 'not informed at all'?

	State of health											
	2019 / very good/good	2020 / very good/good	2021 / very good/good	2022 / very good/good	2023 / very good/good	2024 / very good/good	2019 / at-peace fair/low less good/poor bad	2020 / at-peace good/low good/bad good	2021 / too-peaceful good/low good/bad good	2022 / too-peaceful-good/bad good/bad good	2023 / too-peaceful-good/bad good/bad good	2024 / too-peaceful-good/bad good/bad good
Basis (100%)	572	637	626	589	578	608	428	363	374	411	422	393
very good	8%	11%	18%	15%	12%	15%	10%	10%	17%	13%	11%	7%
good	46%	58%	52%	52%	51%	47%	45%	54%	49%	48%	51%	52%
less good	35%	25%	23%	26%	28%	31%	34%	24%	22%	27%	32%	28%
Not informed at all	11%	6%	6%	7%	8%	6%	11%	11%	10%	12%	5%	11%
wn/kA	-	-	-						2%			1%
total	100%	100%	100%	100%	100%	100%	100%	100%	100%	100%	100%	100%
(very) good	55%	69%	71%	67%	63%	62%	55%	65%	66%	61%	62%	60%
less good/not at all	45%	31%	29%	33%	37%	38%	44%	35%	32%	39%	38%	40%

Question 9: All in all, what do you think: Can you yourself as a patient contribute to ensuring that you receive safe care at the doctor's surgery or hospital?
'yes, definitely' - 'rather yes' - 'rather not' - or
can you as a patient 'not at all' contribute to this?

Basis (100%)	572	637	626	589	578	608	428	363	374	411	422	393
yes, definitely	27%	34%	31%	29%	29%	29%	26%	35%	39%	30%	30%	28%
rather yes	44%	42%	46%	48%	42%	39%	41%	40%	30%	37%	41%	40%
rather not	23%	19%	19%	18%	26%	26%	22%	20%	17%	28%	20%	25%
not at all	5%	5%	4%	5%	3%	6%	9%	4%	13%	4%	9%	7%
wn/kA	1%					1%	3%			1%	-	1%
total	100%	100%	100%	100%	100%	100%	100%	100%	100%	100%	100%	100%
definitely/rather yes	71%	75%	77%	78%	71%	68%	66%	75%	70%	67%	71%	67%
rather not/not at all	28%	24%	23%	22%	29%	31%	30%	25%	30%	32%	29%	32%

f24.0125 Sh/Kr/Ul, May 2024

Question 1: Not applicable

Question 2: 'Patient safety' refers to the successful endeavour to ensure error-free and harm-free medical treatment and medical healthcare. In your opinion, how likely is it that patients in Germany will come to harm as a result of medical treatment in hospital? Do you think this is 'very likely' - 'fairly likely' - 'not very likely' - or
'unlikely'?

	Employment											
	2019 / yes	2020 / yes	2021 / yes	2022 / yes	2023 / yes	2024 / yes	2019 / no	2020 / no	2021 / no	2022 / no	2023 / no	2024 / no
Basis (100%)	524	529	510	514	516	528	476	471	490	487	484	472
Very likely	11%	6%	7%	9%	8%	10%	9%	11%	9%	12%	12%	8%
Fairly likely	37%	24%	21%	21%	21%	24%	33%	24%	17%	19%	21%	20%
not very likely	44%	61%	56%	59%	59%	52%	49%	52%	54%	56%	56%	60%
unlikely	6%	9%	16%	10%	11%	15%	8%	11%	20%	12%	11%	12%
wn/kA	1%						1%	2%	-		1%	
Sum	100%	100%	100%	100%	100%	100%	100%	100%	100%	100%	100%	100%
very/somewhat likely	48%	30%	28%	30%	29%	33%	42%	35%	26%	31%	32%	28%
not very likely/unlikely	50%	70%	72%	70%	70%	66%	58%	63%	74%	69%	67%	71%

Question 3: And in your opinion, how likely is it that patients are harmed by medical care outside a hospital in

Germany, e.g. through outpatient treatment by a doctor or incorrect medication? Do you think this is 'very likely' - 'fairly likely' - 'not very likely'?
'fairly likely' - 'not very likely' - or
'unlikely'?

Basis (100%)	524	529	510	514	516	528	476	471	490	487	484	472
very likely	8%	6%	8%	4%	6%	6%	10%	8%	13%	10%	7%	6%
Fairly likely	29%	19%	26%	25%	26%	27%	31%	30%	16%	26%	23%	21%
not very likely	55%	64%	54%	63%	59%	57%	51%	52%	60%	57%	58%	61%
unlikely	8%	10%	11%	7%	9%	9%	7%	9%	9%	7%	9%	10%
wn/kA						1%		1%	2%		3%	2%
Total	100%	100%	100%	100%	100%	100%	100%	100%	100%	100%	100%	100%
very/somewhat likely	37%	25%	35%	29%	32%	33%	42%	38%	29%	36%	30%	27%
not very likely/unlikely	63%	75%	65%	71%	68%	66%	58%	61%	69%	64%	67%	71%

f24.0125 Sh/Kr/Ul, May 2024

Please tell me in each case whether you think this will happen to you: 'yes, definitely' - 'yes, probably' - 'probably not' - or 'definitely not'? How likely is it that the following will happen to you: ...?

Overview table: yes, definitely/probably

	Employment											
	2019 / yes	2020 / yes	2021 / yes	2022 / yes	2023 / yes	2024 / yes	2019 / no	2020 / no	2021 / no	2022 / no	2023 / no	2024 / no
Basis (100%)	524	529	510	514	516	528	476	471	490	487	484	472
Infection with dangerous germs in hospital	60%	56%	62%	62%	58%	63%	66%	56%	61%	58%	56%	57%
an incorrect diagnosis	61%	52%	57%	53%	66%	56%	58%	48%	48%	47%	55%	52%
an error during an operation, e.g. a surgical error surgical error	39%	30%	32%	26%	25%	25%	45%	33%	37%	24%	32%	33%
an error with medicines due to incorrect prescription, incorrect dosage or incorrect method of use	51%	35%	43%	37%	41%	35%	46%	41%	45%	28%	44%	35%
an error caused by a medical treatment device	28%	16%	26%	17%	18%	17%	33%	29%	22%	17%	26%	18%

Question 4: I will now tell you about possible harmful events in medical care, which are also called
called 'adverse events'.

Please tell me in each case whether you think this will happen to you: 'yes, definitely' - 'yes, probably' - 'probably not' - or 'definitely not'? How likely is it that the following will happen to you: ...?

... an infection with dangerous germs in hospital

Basis (100%)	524	529	510	514	516	528	476	471	490	487	484	472
yes, definitely (incl. has already happened to me)	14%	11%	14%	10%	12%	16%	19%	13%	15%	10%	11%	11%
yes, probably	46%	45%	48%	52%	45%	47%	47%	43%	46%	48%	45%	46%
probably not	34%	37%	33%	34%	37%	32%	27%	33%	33%	36%	38%	38%
definitely not	6%	6%	3%	4%	5%	5%	6%	10%	4%	4%	6%	5%
wn/kA	-	1%	2%	-	-		1%	1%	2%	1%	1%	
Total	100%	100%	100%	100%	100%	100%	100%	100%	100%	100%	100%	100%
yes, definitely/probably	60%	56%	62%	62%	58%	63%	66%	56%	61%	58%	56%	57%
definitely not/probably not	40%	43%	36%	38%	42%	37%	33%	43%	37%	41%	43%	43%

Question 4: I will now tell you about possible harmful events in medical care, which are also called called 'adverse events'.

Please tell me in each case whether you think this will happen to you: 'yes, definitely' - 'yes, probably' - 'probably not' - or 'definitely not'? How likely is it that the following will happen to you happen to you: ...?

f24.0125 Sh/Kr/Ul, May 2024

	Employment											
	2019 / yes	2020 / yes	2021 / yes	2022 / yes	2023 / yes	2024 / yes	2019 / no	2020 / no	2021 / no	2022 / no	2023 / no	2024 / no
... an incorrect diagnosis												
Basis (100%)	524	529	510	514	516	528	476	471	490	487	484	472
yes, definitely (incl. has already happened to me)	15%	12%	16%	10%	18%	13%	13%	17%	10%	12%	16%	13%
yes, probably	45%	40%	41%	43%	48%	42%	45%	32%	38%	35%	39%	39%
probably not	36%	43%	40%	44%	29%	37%	36%	42%	43%	47%	38%	39%
definitely not	3%	4%	3%	4%	5%	7%	7%	9%	8%	6%	6%	8%
wn/kA								1%		-		1%
total	100%	100%	100%	100%	100%	100%	100%	100%	100%	100%	100%	100%
yes, definitely/probably	61%	52%	57%	53%	66%	56%	58%	48%	48%	47%	55%	52%
definitely not/probably not	39%	47%	42%	47%	34%	44%	42%	50%	52%	53%	45%	47%

Question 4: I will now tell you about possible harmful events in medical care, which are also called
called 'adverse events'.

Please tell me in each case whether you think this will happen to you: 'yes, definitely' - 'yes, probably' - 'probably not' - or 'definitely not'? How likely is it that the following will happen to you: ...?

... an error during an operation, e.g. a surgical error

Basis (100%)	524	529	510	514	516	528	476	471	490	487	484	472
yes, definitely (incl. has already happened to me)	7%	3%	8%	3%	6%	5%	6%	11%	7%	8%	5%	4%
yes, probably	32%	27%	24%	23%	20%	20%	39%	22%	30%	16%	27%	29%
probably not	55%	58%	61%	67%	63%	64%	47%	52%	50%	63%	55%	60%
definitely not	6%	12%	7%	7%	12%	10%	8%	14%	11%	12%	13%	7%
wn/kA		1%	1%	-				1%	2%			
Sum	100%	100%	100%	100%	100%	100%	100%	100%	100%	100%	100%	100%
yes, definitely/probably	39%	30%	32%	26%	25%	25%	45%	33%	37%	24%	32%	33%
definitely not/probably not	61%	70%	68%	74%	74%	74%	55%	66%	61%	76%	68%	67%

Question 4: I will now tell you about possible harmful events in medical care, which are also called
called 'adverse events'.

Please tell me in each case whether you think this will happen to you: 'yes, definitely' - 'yes, probably' - 'probably not' - or 'definitely not'? How likely is it that the following will happen to you: ...?

... an error with medicines due to incorrect prescription, incorrect dosage or incorrect method of use

Basis (100%)	524	529	510	514	516	528	476	471	490	487	484	472

f24.0125 Sh/Kr/Ul, May 2024	Employment											
	2019 / yes	2020 / yes	2021 / yes	2022 / yes	2023 / yes	2024 / yes	2019 / no	2020 / no	2021 / no	2022 / no	2023 / no	2024 / no
yes, definitely (incl. has already happened to me)	9%	8%	10%	6%	7%	6%	8%	7%	14%	8%	13%	7%
yes, probably	42%	27%	33%	31%	34%	29%	38%	34%	31%	20%	31%	28%
probably not	41%	52%	48%	52%	49%	55%	45%	44%	42%	57%	43%	50%
definitely not	8%	13%	9%	10%	10%	10%	9%	14%	12%	15%	11%	15%
wn/kA			-				-	1%		-	3%	
total	100%	100%	100%	100%	100%	100%	100%	100%	100%	100%	100%	100%
yes, definitely/probably	51%	35%	43%	37%	41%	35%	46%	41%	45%	28%	44%	35%
definitely not/probably not	49%	65%	57%	63%	59%	65%	54%	58%	55%	72%	54%	65%

Question 4: I will now tell you about possible harmful events in medical care, which are also called
called 'adverse events'.

Please tell me in each case whether you think this will happen to you: 'yes, definitely' - 'yes, probably' - 'probably not' - or 'definitely not'? How likely is it that the following will happen to you: ...?

... an error caused by a medical treatment device

	2019 / yes	2020 / yes	2021 / yes	2022 / yes	2023 / yes	2024 / yes	2019 / no	2020 / no	2021 / no	2022 / no	2023 / no	2024 / no
Basis (100%)	524	529	510	514	516	528	476	471	490	487	484	472
yes, definitely (incl. has already happened to me)	6%	1%	4%	1%	4%	3%	3%	4%	2%	2%	3%	3%
yes, probably	22%	15%	22%	17%	15%	14%	30%	24%	20%	15%	23%	15%
probably not	59%	67%	64%	65%	67%	69%	57%	58%	60%	67%	60%	64%
definitely not	13%	16%	9%	18%	14%	14%	10%	12%	15%	16%	13%	18%
wn/kA		1%	1%		1%			1%	3%		1%	1%
total	100%	100%	100%	100%	100%	100%	100%	100%	100%	100%	100%	100%
yes, definitely/probably	28%	16%	26%	17%	18%	17%	33%	29%	22%	17%	26%	18%
definitely not/probably not	72%	82%	73%	83%	81%	83%	67%	70%	75%	83%	73%	82%

Question 5: I will now read these 'adverse events' to you again. Please tell me in each case whether you think that this can be largely avoided in future by

'suitable measures': 'yes, definitely' - 'yes, probably' - 'probably not' - or 'definitely not'? Can the following be largely avoided in future ... ?

f24.0125 Sh/Kr/Ul, May 2024

Overview table: yes, definitely/probably

	Employment											
	2019 / yes	2020 / yes	2021 / yes	2022 / yes	2023 / yes	2024 / yes	2019 / no	2020 / no	2021 / no	2022 / no	2023 / no	2024 / no
Basis (100%)	524	529	510	514	516	528	476	471	490	487	484	472
Infection with dangerous germs in hospital	58%	59%	65%	51%	55%	48%	69%	62%	69%	54%	69%	65%
an incorrect diagnosis	58%	55%	59%	56%	59%	57%	60%	62%	62%	49%	53%	61%
an error during an operation, e.g. a surgical error	54%	58%	63%	59%	65%	60%	56%	62%	53%	51%	56%	56%
an error with medicinal products due to incorrect prescription, incorrect dosage or incorrect method of use	65%	55%	64%	62%	63%	63%	59%	56%	64%	57%	55%	64%
an error caused by a medical treatment device	60%	63%	68%	70%	66%	67%	59%	56%	61%	54%	56%	59%

Question 5: I will now read these 'adverse events' to you again. Please tell me in each case whether you think that this can be largely avoided in future by 'suitable measures': 'yes, definitely' - 'yes, probably' - 'probably not' - or 'definitely not'? Can the following be largely avoided in future ... ?

... infection with dangerous germs in hospital

	2019 / yes	2020 / yes	2021 / yes	2022 / yes	2023 / yes	2024 / yes	2019 / no	2020 / no	2021 / no	2022 / no	2023 / no	2024 / no
Basis (100%)	524	529	510	514	516	528	476	471	490	487	484	472
yes, definitely	19%	13%	15%	12%	15%	12%	23%	13%	12%	14%	18%	17%
yes, probably	39%	46%	50%	39%	40%	36%	47%	48%	57%	39%	51%	48%
probably not	32%	35%	28%	39%	39%	40%	25%	31%	27%	37%	26%	30%
definitely not	10%	6%	7%	10%	6%	11%	5%	7%	4%	10%	6%	5%
wn/kA		-	-	-	-	-		-	-	-	-	-
Total	100%	100%	100%	100%	100%	100%	100%	100%	100%	100%	100%	100%
yes, definitely/probably	58%	59%	65%	51%	55%	48%	69%	62%	69%	54%	69%	65%
definitely not/probably not	41%	41%	35%	49%	45%	52%	30%	38%	31%	46%	31%	35%

Question 5: I will now read these 'adverse events' to you again. Please tell me in each case whether you think that this can be largely avoided in future by
'suitable measures': 'yes, definitely' - 'yes, probably' - 'probably not' - or 'definitely not'? Can the following be largely avoided in future ... ?

... an incorrect diagnosis

Basis (100%)	524	529	510	514	516	528	476	471	490	487	484	472
yes, definitely	17%	15%	14%	15%	17%	15%	17%	14%	17%	14%	13%	17%
yes, probably	41%	40%	44%	41%	42%	42%	43%	48%	45%	36%	40%	44%
probably not	37%	37%	34%	37%	34%	34%	30%	31%	33%	41%	42%	35%
definitely not	4%	7%	7%	7%	7%	8%	8%	6%	5%	9%	4%	4%
wn/kA			1%	-			1%	1%			1%	
Sum	100%	100%	100%	100%	100%	100%	100%	100%	100%	100%	100%	100%
yes, definitely/probably	58%	55%	59%	56%	59%	57%	60%	62%	62%	49%	53%	61%
definitely not/probably not	42%	44%	40%	44%	41%	43%	39%	37%	37%	50%	46%	39%

f24.0125 Sh/Kr/Ul, May 2024

Question 5: I will now read you these 'undesirable events' once again. Please tell me in each case whether you think that this can be largely avoided in future by 'suitable measures': 'yes, definitely' - 'yes, probably' - 'probably not' - or 'definitely not'? Can the following be largely avoided in future ... ?

... an error during an operation, e.g. a surgical error

	Employment											
	2019 / yes	2020 / yes	2021 / yes	2022 / yes	2023 / yes	2024 / yes	2019 / no	2020 / no	2021 / no	2022 / no	2023 / no	2024 / no
Basis (100%)	524	529	510	514	516	528	476	471	490	487	484	472
yes, definitely	15%	12%	18%	17%	19%	20%	18%	17%	11%	12%	11%	13%
yes, probably	39%	46%	45%	42%	45%	40%	38%	45%	41%	38%	45%	43%
probably not	38%	33%	30%	34%	30%	30%	34%	29%	37%	39%	38%	38%
definitely not	7%	8%	6%	7%	5%	10%	8%	8%	10%	9%	6%	5%
wn/kA	1%	1%	1%	-			2%	2%	1%	2%		1%
Total	100%	100%	100%	100%	100%	100%	100%	100%	100%	100%	100%	100%
yes, definitely/probably	54%	58%	63%	59%	65%	60%	56%	62%	53%	51%	56%	56%
definitely not/probably not	45%	41%	37%	41%	35%	40%	43%	37%	47%	48%	44%	43%

Question 5: I will now read these 'adverse events' to you again. Please tell me in each case whether you think that this can be largely avoided in future by 'suitable measures': 'yes, definitely' - 'yes, probably' - 'probably not' - or 'definitely not'? Can the following be largely avoided in future ... ?

... an error with medicines due to incorrect prescription, incorrect dosage or incorrect method of use

Basis (100%)	524	529	510	514	516	528	476	471	490	487	484	472
yes, definitely	22%	20%	18%	20%	21%	21%	18%	18%	19%	18%	16%	22%
yes, probably	43%	35%	45%	42%	42%	41%	41%	38%	45%	39%	40%	42%
probably not	28%	37%	28%	30%	33%	30%	35%	33%	30%	35%	40%	30%
definitely not	6%	7%	6%	8%	4%	7%	5%	9%	6%	9%	4%	4%
wn/kA			2%	-			1%	2%		-		1%
total	100%	100%	100%	100%	100%	100%	100%	100%	100%	100%	100%	100%
yes, definitely/probably	65%	55%	64%	62%	63%	63%	59%	56%	64%	57%	55%	64%
definitely not/probably not	34%	45%	34%	38%	37%	37%	40%	42%	36%	43%	44%	35%

f24.0125 Sh/Kr/Ul, May 2024

'suitable measures' can be largely avoided: 'yes, definitely' - 'yes, probably' - 'probably not' - or 'definitely not'? Can the following be largely avoided in future ... ?

... an error caused by a medical treatment device

	Employment											
	2019 / yes	2020 / yes	2021 / yes	2022 / yes	2023 / yes	2024 / yes	2019 / no	2020 / no	2021 / no	2022 / no	2023 / no	2024 / no
Basis (100%)	524	529	510	514	516	528	476	471	490	487	484	472
yes, definitely	20%	21%	22%	21%	26%	24%	16%	20%	14%	15%	14%	19%
yes, probably	40%	43%	46%	49%	40%	43%	43%	37%	47%	39%	43%	41%
probably not	29%	27%	27%	24%	28%	25%	34%	33%	29%	35%	37%	34%
definitely not	8%	8%	5%	6%	5%	7%	6%	7%	7%	10%	6%	5%
wn/kA	2%	1%	1%		1%		1%	3%	3%		1%	1%
Sum	100%	100%	100%	100%	100%	100%	100%	100%	100%	100%	100%	100%
yes, definitely/probably	60%	63%	68%	70%	66%	67%	59%	56%	61%	54%	56%	59%
definitely not/probably not	38%	35%	32%	30%	33%	32%	40%	41%	37%	45%	43%	39%

Question 6 and question 7: Not applicable

Question 8: We have now talked a little about the topic of 'patient safety'. How well informed do you feel about patient safety in general: 'very well' - 'well' - 'less well' - or 'not informed at all'?
'well' - 'less well' - or 'not informed at all'?

Basis (100%)	524	529	510	514	516	528	476	471	490	487	484	472
Very good	11%	10%	16%	12%	12%	10%	7%	12%	19%	17%	11%	13%
good	39%	55%	47%	50%	45%	46%	53%	59%	55%	51%	57%	53%
less good	36%	28%	28%	27%	33%	32%	32%	20%	18%	26%	27%	28%
Not informed at all	13%	7%	9%	11%	10%	11%	8%	9%	6%	6%	4%	5%
wn/kA			-			1%	-		1%			1%
total	100%	100%	100%	100%	100%	100%	100%	100%	100%	100%	100%	100%
(very) good	51%	65%	63%	61%	58%	56%	60%	71%	75%	68%	68%	67%
less good/not at all	49%	35%	37%	39%	42%	43%	40%	29%	24%	32%	31%	33%

Question 9: All in all, what do you think: As a patient, can you yourself contribute to ensuring that you receive safe care at the doctor's surgery or hospital?
'yes, definitely' - 'rather yes' - 'rather not' - or can you as a patient 'not at all' contribute to this?

Basis (100%)	524	529	510	514	516	528	476	471	490	487	484	472
yes, definitely	22%	33%	33%	31%	28%	25%	32%	36%	35%	29%	31%	33%
rather yes	45%	39%	41%	43%	42%	40%	40%	43%	38%	45%	41%	38%
rather not	27%	23%	19%	20%	26%	26%	18%	16%	17%	24%	21%	24%
not at all	5%	5%	6%	7%	4%	8%	7%	5%	9%	2%	7%	4%
wn/kA	1%					1%	2%			1%		
total	100%	100%	100%	100%	100%	100%	100%	100%	100%	100%	100%	100%

f24.0125 Sh/Kr/Ul, May 2024

	Employment											
	2019 / yes	2020 / yes	2021 / yes	2022 / yes	2023 / yes	2024 / yes	2019 / no	2020 / no	2021 / no	2022 / no	2023 / no	2024 / no
definitely / rather yes	67%	72%	74%	73%	70%	65%	72%	79%	74%	73%	72%	71%
rather not/not at all	32%	28%	26%	27%	30%	34%	26%	21%	26%	26%	28%	28%

f24.0125 Sh/Kr/Ul, May 2024

Question 1: Not applicable

Question 2: 'Patient safety' refers to the successful endeavour to ensure error-free and harm-free medical treatment and medical healthcare. In your opinion, how likely is it that patients in Germany will come to harm as a result of medical treatment in hospital? Do you think this is 'very likely' - 'fairly likely' - 'not very likely' - or 'unlikely'?

	Chronic patients											
	2019 / yes	2020 / yes	2021 / yes	2022 / yes	2023 / yes	2024 / yes	2019 / no	2020 / no	2021 / no	2022 / no	2023 / no	2024 / no
Basis (100%)	366	356	320	342	339	314	634	643	676	658	660	686
very likely	9%	14%	12%	12%	14%	10%	11%	5%	6%	10%	8%	9%
Fairly likely	39%	26%	28%	25%	22%	20%	33%	23%	15%	18%	20%	23%
not very likely	45%	51%	43%	53%	57%	58%	48%	60%	60%	60%	57%	54%
unlikely	6%	9%	17%	10%	6%	12%	8%	11%	18%	12%	14%	14%
wn/kA	1%	-	-		1%		1%	1%				
total	100%	100%	100%	100%	100%	100%	100%	100%	100%	100%	100%	100%
very/somewhat likely	48%	40%	40%	37%	36%	29%	43%	28%	21%	27%	28%	32%
not very likely/unlikely	51%	60%	60%	63%	63%	70%	55%	71%	79%	72%	71%	68%

Question 3: And in your opinion, how likely is it that patients are harmed by medical care outside a hospital in

Germany, e.g. through outpatient treatment by a doctor or incorrect medication? Do you think this is 'very likely' - 'fairly likely' - 'not very likely'?
'fairly likely' - 'not very likely' - or
'unlikely'?

Basis (100%)	366	356	320	342	339	314	634	643	676	658	660	686
very likely	13%	10%	15%	9%	10%	5%	7%	5%	9%	6%	5%	7%
Fairly likely	31%	30%	28%	29%	29%	25%	29%	21%	18%	23%	22%	24%
not very likely	48%	53%	51%	55%	54%	61%	56%	61%	60%	63%	61%	58%
unlikely	7%	6%	6%	6%	5%	8%	7%	12%	12%	7%	11%	10%
wn/kA					2%	1%		1%	1%		1%	1%
total	100%	100%	100%	100%	100%	100%	100%	100%	100%	100%	100%	100%
very/somewhat likely	44%	40%	43%	38%	39%	30%	36%	26%	27%	29%	27%	30%
not very likely/unlikely	56%	60%	57%	61%	59%	69%	63%	73%	72%	71%	72%	68%

Question 4: I will now tell you about possible harmful events in medical care, which are also called
called 'adverse events'.

f24.0125 Sh/Kr/Ul, May 2024

Please tell me in each case whether you think this will happen to you: 'yes, definitely' - 'yes, probably' - 'probably not' - or 'definitely not'? How likely is it that the following will happen to you: ...?

Overview table: yes, definitely/probably

	Chronic patients											
	2019 / yes	2020 / yes	2021 / yes	2022 / yes	2023 / yes	2024 / yes	2019 / no	2020 / no	2021 / no	2022 / no	2023 / no	2024 / no
Basis (100%)	366	356	320	342	339	314	634	643	676	658	660	686
Infection with dangerous germs in hospital	72%	58%	69%	63%	63%	63%	58%	55%	58%	58%	54%	59%
an incorrect diagnosis	61%	52%	57%	53%	65%	56%	58%	50%	51%	49%	59%	53%
an error during an operation, e.g. a surgical error	53%	32%	42%	34%	36%	31%	35%	31%	31%	20%	25%	28%
an error with medicines due to incorrect prescription, incorrect dosage or incorrect method of use	50%	40%	47%	36%	53%	37%	48%	36%	42%	31%	37%	34%
an error caused by a medical treatment device	39%	27%	30%	20%	35%	21%	25%	20%	22%	15%	15%	16%

Question 4: I will now tell you about possible harmful events in medical care, which are also called
called 'adverse events'.

Please tell me in each case whether you think this will happen to you: 'yes, definitely' - 'yes, probably' - 'probably not' - or 'definitely not'? How likely is it that the following will happen to you: ...?

... an infection with dangerous germs in hospital

Basis (100%)	366	356	320	342	339	314	634	643	676	658	660	686
yes, definitely (incl. has already happened to me)	22%	14%	20%	13%	11%	15%	13%	11%	12%	8%	12%	13%
yes, probably	49%	44%	49%	50%	52%	48%	45%	44%	46%	50%	42%	45%
probably not	23%	32%	27%	33%	35%	31%	35%	37%	36%	36%	38%	36%
definitely not	5%	9%	1%	2%	2%	6%	6%	7%	5%	6%	7%	5%
wn/kA		1%	3%	2%	-		1%	1%	1%	-		
Total	100%	100%	100%	100%	100%	100%	100%	100%	100%	100%	100%	100%
yes, definitely/probably	72%	58%	69%	63%	63%	63%	58%	55%	58%	58%	54%	59%
definitely not/probably not	28%	41%	28%	35%	37%	37%	41%	44%	41%	42%	46%	41%

Question 4: I will now tell you about possible harmful events in medical care, which are also called
called 'adverse events'.

Please tell me in each case whether you think this will happen to you: 'yes, definitely' - 'yes, probably' - 'probably not' - or 'definitely not'? How likely is it that the following will happen to you
happen to you: ...?

f24.0125 Sh/Kr/Ul, May 2024						Chronic patients						
	2019 / yes	2020 / yes	2021 / yes	2022 / yes	2023 / yes	2024 / yes	2019 / no	2020 / no	2021 / no	2022 / no	2023 / no	2024 / no
... an incorrect diagnosis												
Basis (100%)	366	356	320	342	339	314	634	643	676	658	660	686
yes, definitely (incl. has already happened to me)	17%	19%	17%	15%	21%	16%	13%	12%	11%	9%	15%	12%
yes, probably	43%	34%	40%	37%	43%	40%	46%	38%	40%	40%	44%	41%
probably not	33%	41%	37%	43%	30%	35%	37%	43%	44%	46%	35%	39%
definitely not	6%	7%	5%	4%	5%	9%	4%	6%	5%	5%	6%	7%
wn/kA				-		1%		1%				1%
total	100%	100%	100%	100%	100%	100%	100%	100%	100%	100%	100%	100%
yes, definitely/probably	61%	52%	57%	53%	65%	56%	58%	50%	51%	49%	59%	53%
definitely not/probably not	39%	48%	43%	47%	35%	44%	41%	49%	49%	51%	41%	46%

Question 4: I will now tell you about possible harmful events in medical care, which are also called
called 'adverse events'.

Please tell me in each case whether you think this will happen to you: 'yes, definitely' - 'yes, probably' - 'probably not' - or 'definitely not'? How likely is it that the following will happen to you: ...?

... an error during an operation, e.g. a surgical error

Basis (100%)	366	356	320	342	339	314	634	643	676	658	660	686
yes, definitely (incl. has already happened to me)	9%	11%	15%	11%	5%	4%	4%	4%	4%	3%	6%	5%
yes, probably	43%	21%	26%	23%	31%	27%	31%	26%	27%	18%	19%	23%
probably not	41%	55%	47%	57%	53%	61%	57%	55%	59%	69%	62%	62%
definitely not	6%	12%	9%	9%	11%	7%	7%	14%	9%	10%	13%	9%
wn/kA		1%	2%				1%	1%				
total	100%	100%	100%	100%	100%	100%	100%	100%	100%	100%	100%	100%
yes, definitely/probably	53%	32%	42%	34%	36%	31%	35%	31%	31%	20%	25%	28%
definitely not/probably not	47%	67%	56%	66%	64%	69%	64%	69%	69%	79%	75%	72%

Question 4: I will now tell you about possible harmful events in medical care, which are also called
called 'adverse events'.

Please tell me in each case whether you think this will happen to you: 'yes, definitely' - 'yes, probably' - 'probably not' - or 'definitely not'? How likely is it that the following will happen to you: ...?

... an error with medicines due to incorrect prescription, incorrect dosage or incorrect method of use

f24.0125 Sh/Kr/Ul, May 2024	Chronic patients											
	2019 / yes	2020 / yes	2021 / yes	2022 / yes	2023 / yes	2024 / yes	2019 / no	2020 / no	2021 / no	2022 / no	2023 / no	2024 / no
yes, definitely (incl. has already happened to me)	11%	10%	17%	11%	15%	9%	7%	6%	9%	5%	7%	6%
yes, probably	39%	30%	31%	25%	38%	28%	41%	30%	33%	26%	30%	28%
probably not	39%	43%	42%	54%	35%	49%	45%	51%	47%	54%	51%	54%
definitely not	11%	17%	11%	9%	9%	13%	7%	12%	11%	14%	11%	12%
wn/kA	-			-	3%			1%				
total	100%	100%	100%	100%	100%	100%	100%	100%	100%	100%	100%	100%
yes, definitely/probably	50%	40%	47%	36%	53%	37%	48%	36%	42%	31%	37%	34%
definitely not/probably not	50%	59%	52%	64%	44%	62%	52%	63%	58%	69%	62%	66%

Question 4: I will now tell you about possible harmful events in medical care, which are also called
called 'adverse events'.

Please tell me in each case whether you think this will happen to you: 'yes, definitely' - 'yes, probably' - 'probably not' - or 'definitely not'? How likely is it that the following will happen to you: ...?

... an error caused by a medical treatment device

Basis (100%)	366	356	320	342	339	314	634	643	676	658	660	686
yes, definitely (incl. has already happened to me)	4%	2%	3%	1%	6%	4%	5%	3%	3%	1%	2%	2%
yes, probably	35%	25%	27%	19%	29%	17%	21%	17%	18%	14%	13%	14%
probably not	50%	59%	54%	67%	52%	60%	62%	64%	66%	65%	69%	69%
definitely not	11%	13%	14%	13%	12%	19%	12%	15%	11%	20%	15%	14%
wn/kA		1%	2%	-				1%	2%		1%	1%
total	100%	100%	100%	100%	100%	100%	100%	100%	100%	100%	100%	100%
yes, definitely/probably	39%	27%	30%	20%	35%	21%	25%	20%	22%	15%	15%	16%
definitely not/probably not	61%	72%	68%	80%	65%	78%	74%	79%	77%	85%	84%	84%

Question 5: I will now read these 'adverse events' to you again. Please tell me in each case whether you think that this can be largely avoided in future by 'suitable measures': 'yes, definitely' - 'yes, probably' - 'probably not' - or 'definitely not'? Can the following be largely avoided in future ... ?

Overview table: yes, definitely/probably

Basis (100%)	366	356	320	342	339	314	634	643	676	658	660	686
Infection with dangerous germs in hospital	67%	59%	68%	55%	61%	59%	61%	61%	66%	51%	62%	55%
an incorrect diagnosis	59%	62%	63%	48%	49%	56%	59%	57%	59%	55%	60%	60%
an error during an operation, e.g. a surgical error	60%	57%	57%	58%	52%	58%	52%	61%	58%	53%	65%	58%

f24.0125 Sh/Kr/Ul, May 2024	Chronic patients											
	2019 / yes	2020 / yes	2021 / yes	2022 / yes	2023 / yes	2024 / yes	2019 / no	2020 / no	2021 / no	2022 / no	2023 / no	2024 / no
an error with medicinal products due to incorrect prescription, incorrect dosage or incorrect method of use	60%	56%	66%	58%	54%	59%	63%	55%	63%	60%	62%	65%
an error caused by a medical treatment device	52%	57%	59%	58%	56%	55%	63%	62%	67%	65%	64%	67%

Question 5: I will now read these 'adverse events' to you again. Please tell me now in each case whether you think that this can be largely avoided in future by 'suitable measures': 'yes, definitely' - 'yes, probably' - 'probably not' - or 'definitely not'? Can the following be largely avoided in future ... ?

... infection with dangerous germs in hospital	2019 / yes	2020 / yes	2021 / yes	2022 / yes	2023 / yes	2024 / yes	2019 / no	2020 / no	2021 / no	2022 / no	2023 / no	2024 / no
Basis (100%)	366	356	320	342	339	314	634	643	676	658	660	686
yes, definitely	25%	16%	16%	18%	16%	8%	18%	12%	12%	11%	17%	17%
yes, probably	42%	43%	52%	38%	45%	51%	43%	49%	54%	40%	45%	38%
probably not	26%	32%	24%	35%	31%	32%	30%	34%	29%	39%	33%	37%
definitely not	7%	9%	8%	10%	8%	8%	8%	5%	5%	10%	5%	8%
wn/kA		-	-	-	-	-		-	-	-	-	-
Total	100%	100%	100%	100%	100%	100%	100%	100%	100%	100%	100%	100%
yes, definitely/probably	67%	59%	68%	55%	61%	59%	61%	61%	66%	51%	62%	55%
definitely not/probably not	33%	41%	32%	45%	39%	41%	38%	39%	34%	49%	38%	45%

Question 5: I will now read these 'adverse events' to you again. Please tell me in each case whether you think that this can be largely avoided in future by 'suitable measures': 'yes, definitely' - 'yes, probably' - 'probably not' - or 'definitely not'? Can the following be largely avoided in future ... ?

... an incorrect diagnosis

Basis (100%)	366	356	320	342	339	314	634	643	676	658	660	686
yes, definitely	18%	16%	14%	14%	12%	10%	17%	14%	17%	14%	17%	18%
yes, probably	41%	46%	50%	34%	38%	46%	42%	42%	43%	41%	43%	42%
probably not	32%	30%	29%	43%	45%	39%	35%	37%	35%	37%	34%	33%
definitely not	8%	8%	7%	9%	4%	5%	5%	6%	5%	8%	6%	7%
wn/kA	1%		1%	-	1%		1%	1%	1%			
total	100%	100%	100%	100%	100%	100%	100%	100%	100%	100%	100%	100%
yes, definitely/probably	59%	62%	63%	48%	49%	56%	59%	57%	59%	55%	60%	60%
definitely not/probably not	40%	37%	36%	52%	49%	44%	40%	42%	40%	45%	40%	40%

f24.0125 Sh/Kr/Ul, May 2024

Question 5: I will now read you these 'undesirable events' once again. Please tell me in each case whether you think that this can be largely avoided in future by
'suitable measures': 'yes, definitely' - 'yes, probably' - 'probably not' - or 'definitely not'? Can the following be largely avoided in future ... ?

... an error during an operation, e.g. a surgical error

	Chronic patients											
	2019 / yes	2020 / yes	2021 / yes	2022 / yes	2023 / yes	2024 / yes	2019 / no	2020 / no	2021 / no	2022 / no	2023 / no	2024 / no
Basis (100%)	366	356	320	342	339	314	634	643	676	658	660	686
yes, definitely	18%	16%	10%	16%	13%	12%	15%	14%	17%	14%	17%	19%
yes, probably	42%	42%	47%	42%	39%	46%	37%	47%	42%	39%	48%	39%
probably not	33%	33%	34%	34%	43%	35%	39%	30%	33%	38%	29%	33%
definitely not	7%	10%	9%	6%	5%	5%	8%	7%	8%	9%	6%	9%
wn/kA	1%		1%	2%		1%	1%	2%	1%			
Total	100%	100%	100%	100%	100%	100%	100%	100%	100%	100%	100%	100%
yes, definitely/probably	60%	57%	57%	58%	52%	58%	52%	61%	58%	53%	65%	58%
definitely not/probably not	39%	43%	42%	40%	48%	40%	47%	37%	41%	46%	35%	42%

Question 5: I will now read these 'adverse events' to you again. Please tell me in each case whether you think that this can be largely avoided in future by
'suitable measures': 'yes, definitely' - 'yes, probably' - 'probably not' - or 'definitely not'? Can the following be largely avoided in future ... ?

... an error with medicines due to incorrect prescription, incorrect dosage or incorrect method of use

	2019 / yes	2020 / yes	2021 / yes	2022 / yes	2023 / yes	2024 / yes	2019 / no	2020 / no	2021 / no	2022 / no	2023 / no	2024 / no
Basis (100%)	366	356	320	342	339	314	634	643	676	658	660	686
yes, definitely	23%	21%	19%	21%	14%	19%	19%	18%	19%	18%	20%	23%
yes, probably	37%	35%	47%	37%	41%	40%	44%	37%	44%	42%	41%	43%
probably not	32%	33%	27%	34%	41%	34%	32%	37%	29%	32%	34%	28%
definitely not	7%	10%	6%	8%	4%	5%	4%	7%	6%	8%	4%	6%
wn/kA		1%	1%	-		1%	1%	1%	2%	-		
Total	100%	100%	100%	100%	100%	100%	100%	100%	100%	100%	100%	100%
yes, definitely/probably	60%	56%	66%	58%	54%	59%	63%	55%	63%	60%	62%	65%
definitely not/probably not	39%	43%	33%	42%	45%	40%	36%	44%	35%	40%	38%	34%

Question 5: I will now read these 'adverse events' to you again. Please tell me in each case whether you think this will be minimised in future by

f24.0125 Sh/Kr/Ul, May 2024

'suitable measures' can be largely avoided: 'yes, definitely' - 'yes, probably' - 'probably not' - or 'definitely not'? Can the following be largely avoided in future ... ?

... an error caused by a medical treatment device

						Chronic patients						
	2019 / yes	2020 / yes	2021 / yes	2022 / yes	2023 / yes	2024 / yes	2019 / no	2020 / no	2021 / no	2022 / no	2023 / no	2024 / no
Basis (100%)	366	356	320	342	339	314	634	643	676	658	660	686
yes, definitely	18%	19%	15%	17%	14%	18%	18%	21%	19%	19%	24%	23%
yes, probably	35%	38%	44%	42%	43%	37%	45%	41%	48%	46%	41%	44%
probably not	37%	32%	30%	31%	36%	34%	28%	29%	27%	28%	30%	27%
definitely not	8%	9%	8%	10%	8%	9%	7%	7%	5%	7%	4%	6%
wn/kA	2%	2%	3%			2%	2%	2%	1%		2%	
Total	100%	100%	100%	100%	100%	100%	100%	100%	100%	100%	100%	100%
yes, definitely/probably	52%	57%	59%	58%	56%	55%	63%	62%	67%	65%	64%	67%
definitely not/probably not	46%	41%	38%	41%	44%	43%	35%	36%	32%	35%	34%	32%

Question 6 and question 7: Not applicable

Question 8: We have now talked a little about the topic of 'patient safety'. How well informed do you feel about patient safety in general: 'very well' - 'well' - 'less well' - or 'not informed at all'?
'well' - 'less well' - or 'not informed at all'?

Basis (100%)	366	356	320	342	339	314	634	643	676	658	660	686
Very good	11%	15%	20%	16%	14%	14%	8%	8%	17%	13%	10%	11%
good	53%	55%	58%	48%	55%	56%	41%	58%	48%	51%	49%	47%
less good	28%	23%	14%	25%	26%	26%	38%	25%	27%	27%	32%	32%
Not informed at all	8%	7%	9%	10%	5%	4%	12%	8%	7%	8%	8%	10%
wn/kA		-	-			1%			1%			
sum	100%	100%	100%	100%	100%	100%	100%	100%	100%	100%	100%	100%
(very) good	64%	70%	78%	64%	69%	70%	50%	66%	65%	64%	60%	57%
less good/not at all	36%	30%	22%	35%	31%	30%	50%	34%	34%	35%	40%	42%

Question 9: All in all, what do you think: As a patient, can you yourself contribute to ensuring that you receive safe care at the doctor's surgery or hospital?
'yes, definitely' - 'rather yes' - 'rather not' - or can you as a patient 'not at all' contribute to this?

Basis (100%)	366	356	320	342	339	314	634	643	676	658	660	686
yes, definitely	32%	42%	46%	39%	33%	33%	23%	30%	29%	25%	27%	26%
rather yes	42%	37%	32%	35%	42%	44%	43%	43%	44%	48%	41%	37%
rather not	19%	15%	16%	23%	17%	18%	25%	22%	19%	21%	27%	28%
not at all	6%	5%	7%	3%	8%	5%	6%	5%	8%	5%	4%	7%
wn/kA	2%				-		2%			1%		1%

	Chronic patients											
f24.0125 Sh/Kr/Ul, May 2024	2019 / yes	2020 / yes	2021 / yes	2022 / yes	2023 / yes	2024 / yes	2019 / no	2020 / no	2021 / no	2022 / no	2023 / no	2024 / no
definitely / rather yes	74%	79%	77%	73%	75%	77%	67%	73%	72%	73%	69%	64%
rather not/not at all	25%	21%	22%	26%	25%	23%	31%	27%	27%	26%	31%	35%

f24.0125 Sh/Kr/Ul, May 2024

	Chronic patients											
	2019 / yes	2020 / yes	2021 / yes	2022 / yes	2023 / yes	2024 / yes	2019 / no	2020 / no	2021 / no	2022 / no	2023 / no	2024 / no

Question 1: Not applicable

Question 2: 'Patient safety' refers to the successful endeavour to ensure error-free and harm-free medical treatment and medical healthcare. In your opinion, how likely is it that patients in Germany will come to harm as a result of medical treatment in hospital? Do you think this is 'very likely' - 'fairly likely' - 'not very likely' - or 'unlikely'?

	2019 / yes	2020 / yes	2021 / yes	2022 / yes	2023 / yes	2024 / yes	2019 / no	2020 / no	2021 / no	2022 / no	2023 / no	2024 / no
Basis (100%)	366	356	320	342	339	314	634	643	676	658	660	686
very likely	9%	14%	12%	12%	14%	10%	11%	5%	6%	10%	8%	9%
Fairly likely	39%	26%	28%	25%	22%	20%	33%	23%	15%	18%	20%	23%
not very likely	45%	51%	43%	53%	57%	58%	48%	60%	60%	60%	57%	54%
unlikely	6%	9%	17%	10%	6%	12%	8%	11%	18%	12%	14%	14%
wn/kA	1%	-	-		1%		1%	1%				
Total	100%	100%	100%	100%	100%	100%	100%	100%	100%	100%	100%	100%
very/somewhat likely	48%	40%	40%	37%	36%	29%	43%	28%	21%	27%	28%	32%
not very likely/unlikely	51%	60%	60%	63%	63%	70%	55%	71%	79%	72%	71%	68%

Question 3: And in your opinion, how likely is it that patients are harmed by medical care outside a hospital in

Germany, e.g. through outpatient treatment by a doctor or incorrect medication? Do you think this is 'very likely' - 'fairly likely' - 'not very likely'?
'fairly likely' - 'not very likely' - or
'unlikely'?

Basis (100%)	366	356	320	342	339	314	634	643	676	658	660	686
very likely	13%	10%	15%	9%	10%	5%	7%	5%	9%	6%	5%	7%
Fairly likely	31%	30%	28%	29%	29%	25%	29%	21%	18%	23%	22%	24%
not very likely	48%	53%	51%	55%	54%	61%	56%	61%	60%	63%	61%	58%
unlikely	7%	6%	6%	6%	5%	8%	7%	12%	12%	7%	11%	10%
wn/kA					2%	1%		1%	1%		1%	1%
total	100%	100%	100%	100%	100%	100%	100%	100%	100%	100%	100%	100%
very/somewhat likely	44%	40%	43%	38%	39%	30%	36%	26%	27%	29%	27%	30%
not very likely/unlikely	56%	60%	57%	61%	59%	69%	63%	73%	72%	71%	72%	68%

Question 4: I will now tell you about possible harmful events in medical care, which are also called
called 'adverse events'.

f24.0125 Sh/Kr/Ul, May 2024

Please tell me in each case whether you think this will happen to you: 'yes, definitely' - 'yes, probably' - 'probably not' - or 'definitely not'? How likely is it that the following will happen to you: ...?

Overview table: yes, definitely/probably

	Chronic patients 2019 / yes	2020 / yes	2021 / yes	2022 / yes	2023 / yes	2024 / yes	2019 / no	2020 / no	2021 / no	2022 / no	2023 / no	2024 / no
Basis (100%)	366	356	320	342	339	314	634	643	676	658	660	686
Infection with dangerous germs in hospital	72%	58%	69%	63%	63%	63%	58%	55%	58%	58%	54%	59%
an incorrect diagnosis	61%	52%	57%	53%	65%	56%	58%	50%	51%	49%	59%	53%
an error during an operation, e.g. a surgical error	53%	32%	42%	34%	36%	31%	35%	31%	31%	20%	25%	28%
an error with medicines due to incorrect prescription, incorrect dosage or incorrect method of use	50%	40%	47%	36%	53%	37%	48%	36%	42%	31%	37%	34%
an error caused by a medical treatment device	39%	27%	30%	20%	35%	21%	25%	20%	22%	15%	15%	16%

Question 4: I will now tell you about possible harmful events in medical care, which are also called
called 'adverse events'.

Please tell me in each case whether you think this will happen to you: 'yes, definitely' - 'yes, probably' - 'probably not' - or 'definitely not'? How likely is it that the following will happen to you: ...?

... an infection with dangerous germs in hospital												
Basis (100%)	366	356	320	342	339	314	634	643	676	658	660	686
yes, definitely (incl. has already happened to me)	22%	14%	20%	13%	11%	15%	13%	11%	12%	8%	12%	13%
yes, probably	49%	44%	49%	50%	52%	48%	45%	44%	46%	50%	42%	45%
probably not	23%	32%	27%	33%	35%	31%	35%	37%	36%	36%	38%	36%
definitely not	5%	9%	1%	2%	2%	6%	6%	7%	5%	6%	7%	5%
wn/kA		1%	3%	2%	-		1%	1%	1%	-		
Total	100%	100%	100%	100%	100%	100%	100%	100%	100%	100%	100%	100%
yes, definitely/probably	72%	58%	69%	63%	63%	63%	58%	55%	58%	58%	54%	59%
definitely not/probably not	28%	41%	28%	35%	37%	37%	41%	44%	41%	42%	46%	41%

Question 4: I will now tell you about possible harmful events in medical care, which are also called called 'adverse events'.

Please tell me in each case whether you think this will happen to you: 'yes, definitely' - 'yes, probably' - 'probably not' - or 'definitely not'? How likely is it that the following will happen to you happen to you: ...?

f24.0125 Sh/Kr/Ul, May 2024	Chronic patients											
	2019 / yes	2020 / yes	2021 / yes	2022 / yes	2023 / yes	2024 / yes	2019 / no	2020 / no	2021 / no	2022 / no	2023 / no	2024 / no
... an incorrect diagnosis												
Basis (100%)	366	356	320	342	339	314	634	643	676	658	660	686
yes, definitely (incl. has already happened to me)	17%	19%	17%	15%	21%	16%	13%	12%	11%	9%	15%	12%
yes, probably	43%	34%	40%	37%	43%	40%	46%	38%	40%	40%	44%	41%
probably not	33%	41%	37%	43%	30%	35%	37%	43%	44%	46%	35%	39%
definitely not	6%	7%	5%	4%	5%	9%	4%	6%	5%	5%	6%	7%
wn/kA				-		1%		1%				1%
total	100%	100%	100%	100%	100%	100%	100%	100%	100%	100%	100%	100%
yes, definitely/probably	61%	52%	57%	53%	65%	56%	58%	50%	51%	49%	59%	53%
definitely not/probably not	39%	48%	43%	47%	35%	44%	41%	49%	49%	51%	41%	46%

Question 4: I will now tell you about possible harmful occurrences in medical care, which are also called called 'adverse events'.

Please tell me in each case whether you think this will happen to you: 'yes, definitely' - 'yes, probably' - 'probably not' - or 'definitely not'? How likely is it that the following will happen to you: ...?

... an error during an operation, e.g. a surgical error

Basis (100%)	366	356	320	342	339	314	634	643	676	658	660	686
yes, definitely (incl. has already happened to me)	9%	11%	15%	11%	5%	4%	4%	4%	4%	3%	6%	5%
yes, probably	43%	21%	26%	23%	31%	27%	31%	26%	27%	18%	19%	23%
probably not	41%	55%	47%	57%	53%	61%	57%	55%	59%	69%	62%	62%
definitely not	6%	12%	9%	9%	11%	7%	7%	14%	9%	10%	13%	9%
wn/kA		1%	2%				1%	1%				
total	100%	100%	100%	100%	100%	100%	100%	100%	100%	100%	100%	100%
yes, definitely/probably	53%	32%	42%	34%	36%	31%	35%	31%	31%	20%	25%	28%
definitely not/probably not	47%	67%	56%	66%	64%	69%	64%	69%	69%	79%	75%	72%

Question 4: I will now tell you about possible harmful events in medical care, which are also called
called 'adverse events'.

Please tell me in each case whether you think this will happen to you: 'yes, definitely' - 'yes, probably' - 'probably not' - or 'definitely not'? How likely is it that the following will happen to you: ...?

... an error with medicines due to incorrect prescription, incorrect dosage or incorrect method of use

Basis (100%)	366	356	320	342	339	314	634	643	676	658	660	686

f24.0125 Sh/Kr/Ul, May 2024	Chronic patients											
	2019 / yes	2020 / yes	2021 / yes	2022 / yes	2023 / yes	2024 / yes	2019 / no	2020 / no	2021 / no	2022 / no	2023 / no	2024 / no
yes, definitely (incl. has already happened to me)	11%	10%	17%	11%	15%	9%	7%	6%	9%	5%	7%	6%
yes, probably	39%	30%	31%	25%	38%	28%	41%	30%	33%	26%	30%	28%
probably not	39%	43%	42%	54%	35%	49%	45%	51%	47%	54%	51%	54%
definitely not	11%	17%	11%	9%	9%	13%	7%	12%	11%	14%	11%	12%
wn/kA	-			-	3%			1%				
total	100%	100%	100%	100%	100%	100%	100%	100%	100%	100%	100%	100%
yes, definitely/probably	50%	40%	47%	36%	53%	37%	48%	36%	42%	31%	37%	34%
definitely not/probably not	50%	59%	52%	64%	44%	62%	52%	63%	58%	69%	62%	66%

Question 4: I will now tell you about possible harmful occurrences in medical care, which are also called called 'adverse events'.

Please tell me in each case whether you think this will happen to you: 'yes, definitely' - 'yes, probably' - 'probably not' - or 'definitely not'? How likely is it that the following will happen to you: ...?

... an error caused by a medical treatment device

Basis (100%)	366	356	320	342	339	314	634	643	676	658	660	686
yes, definitely (incl. has already happened to me)	4%	2%	3%	1%	6%	4%	5%	3%	3%	1%	2%	2%
yes, probably	35%	25%	27%	19%	29%	17%	21%	17%	18%	14%	13%	14%
probably not	50%	59%	54%	67%	52%	60%	62%	64%	66%	65%	69%	69%
definitely not	11%	13%	14%	13%	12%	19%	12%	15%	11%	20%	15%	14%
wn/kA		1%	2%	-				1%	2%		1%	1%
total	100%	100%	100%	100%	100%	100%	100%	100%	100%	100%	100%	100%
yes, definitely/probably	39%	27%	30%	20%	35%	21%	25%	20%	22%	15%	15%	16%
definitely not/probably not	61%	72%	68%	80%	65%	78%	74%	79%	77%	85%	84%	84%

Question 5: I will now read these 'adverse events' to you again. Please tell me in each case whether you think that this can be largely avoided in future by 'suitable measures': 'yes, definitely' - 'yes, probably' - 'probably not' - or 'definitely not'? Can the following be largely avoided in future ... ?

Overview table: yes, definitely/probably

Basis (100%)	366	356	320	342	339	314	634	643	676	658	660	686
Infection with dangerous germs in hospital	67%	59%	68%	55%	61%	59%	61%	61%	66%	51%	62%	55%
an incorrect diagnosis	59%	62%	63%	48%	49%	56%	59%	57%	59%	55%	60%	60%
an error during an operation, e.g. a surgical error	60%	57%	57%	58%	52%	58%	52%	61%	58%	53%	65%	58%

f24.0125 Sh/Kr/Ul, May 2024	Chronic patients											
	2019 / yes	2020 / yes	2021 / yes	2022 / yes	2023 / yes	2024 / yes	2019 / no	2020 / no	2021 / no	2022 / no	2023 / no	2024 / no
an error with medicinal products due to incorrect prescription, incorrect dosage or incorrect method of use	60%	56%	66%	58%	54%	59%	63%	55%	63%	60%	62%	65%
an error caused by a medical treatment device	52%	57%	59%	58%	56%	55%	63%	62%	67%	65%	64%	67%

Question 5: I will now read these 'adverse events' to you again. Please tell me in each case whether you think that this can be largely avoided in the future by 'suitable measures': 'yes, definitely' - 'yes, probably' - 'probably not' - or 'definitely not'? Can the following be largely avoided in future ... ?

... infection with dangerous germs in hospital	2019 / yes	2020 / yes	2021 / yes	2022 / yes	2023 / yes	2024 / yes	2019 / no	2020 / no	2021 / no	2022 / no	2023 / no	2024 / no
Basis (100%)	366	356	320	342	339	314	634	643	676	658	660	686
yes, definitely	25%	16%	16%	18%	16%	8%	18%	12%	12%	11%	17%	17%
yes, probably	42%	43%	52%	38%	45%	51%	43%	49%	54%	40%	45%	38%
probably not	26%	32%	24%	35%	31%	32%	30%	34%	29%	39%	33%	37%
definitely not	7%	9%	8%	10%	8%	8%	8%	5%	5%	10%	5%	8%
wn/kA		-	-	-	-	-		-	-	-	-	-
total	100%	100%	100%	100%	100%	100%	100%	100%	100%	100%	100%	100%
yes, definitely/probably	67%	59%	68%	55%	61%	59%	61%	61%	66%	51%	62%	55%
definitely not/probably not	33%	41%	32%	45%	39%	41%	38%	39%	34%	49%	38%	45%

Question 5: I will now read these 'adverse events' to you again. Please tell me in each case whether you think that this can be largely avoided in future by
'suitable measures': 'yes, definitely' - 'yes, probably' - 'probably not' - or 'definitely not'? Can the following be largely avoided in future ... ?

... an incorrect diagnosis

Basis (100%)	366	356	320	342	339	314	634	643	676	658	660	686
yes, definitely	18%	16%	14%	14%	12%	10%	17%	14%	17%	14%	17%	18%
yes, probably	41%	46%	50%	34%	38%	46%	42%	42%	43%	41%	43%	42%
probably not	32%	30%	29%	43%	45%	39%	35%	37%	35%	37%	34%	33%
definitely not	8%	8%	7%	9%	4%	5%	5%	6%	5%	8%	6%	7%
wn/kA	1%		1%	-	1%		1%	1%	1%			
Sum	100%	100%	100%	100%	100%	100%	100%	100%	100%	100%	100%	100%
yes, definitely/probably	59%	62%	63%	48%	49%	56%	59%	57%	59%	55%	60%	60%
definitely not/probably not	40%	37%	36%	52%	49%	44%	40%	42%	40%	45%	40%	40%

f24.0125 Sh/Kr/Ul, May 2024

Question 5: I will now read you these 'undesirable events' once again. Please tell me in each case whether you think that this can be largely avoided in future by 'suitable measures': 'yes, definitely' - 'yes, probably' - 'probably not' - or 'definitely not'? Can the following be largely avoided in future ... ?

... an error during an operation, e.g. a surgical error

	Chronic patients											
	2019 / yes	2020 / yes	2021 / yes	2022 / yes	2023 / yes	2024 / yes	2019 / no	2020 / no	2021 / no	2022 / no	2023 / no	2024 / no
Basis (100%)	366	356	320	342	339	314	634	643	676	658	660	686
yes, definitely	18%	16%	10%	16%	13%	12%	15%	14%	17%	14%	17%	19%
yes, probably	42%	42%	47%	42%	39%	46%	37%	47%	42%	39%	48%	39%
probably not	33%	33%	34%	34%	43%	35%	39%	30%	33%	38%	29%	33%
definitely not	7%	10%	9%	6%	5%	5%	8%	7%	8%	9%	6%	9%
wn/kA	1%		1%	2%		1%	1%	2%	1%			
Total	100%	100%	100%	100%	100%	100%	100%	100%	100%	100%	100%	100%
yes, definitely/probably	60%	57%	57%	58%	52%	58%	52%	61%	58%	53%	65%	58%
definitely not/probably not	39%	43%	42%	40%	48%	40%	47%	37%	41%	46%	35%	42%

Question 5: I will now read these 'adverse events' to you again. Please tell me in each case whether you think that this can be largely avoided in future by 'suitable measures': 'yes, definitely' - 'yes, probably' - 'probably not' - or 'definitely not'? Can the following be largely avoided in future ... ?

... an error with medicines due to incorrect prescription, incorrect dosage or incorrect method of use

Basis (100%)	366	356	320	342	339	314	634	643	676	658	660	686
yes, definitely	23%	21%	19%	21%	14%	19%	19%	18%	19%	18%	20%	23%
yes, probably	37%	35%	47%	37%	41%	40%	44%	37%	44%	42%	41%	43%
probably not	32%	33%	27%	34%	41%	34%	32%	37%	29%	32%	34%	28%
definitely not	7%	10%	6%	8%	4%	5%	4%	7%	6%	8%	4%	6%
wn/kA		1%	1%	-		1%	1%	1%	2%	-		
Total	100%	100%	100%	100%	100%	100%	100%	100%	100%	100%	100%	100%
yes, definitely/probably	60%	56%	66%	58%	54%	59%	63%	55%	63%	60%	62%	65%
definitely not/probably not	39%	43%	33%	42%	45%	40%	36%	44%	35%	40%	38%	34%

Question 5: I will now read these 'adverse events' to you again. Please tell me in each case whether you think this will be minimised in future by

f24.0125 Sh/Kr/Ul, May 2024

'suitable measures' can be largely avoided: 'yes, definitely' - 'yes, probably' - 'probably not' - or 'definitely not'? Can the following be largely avoided in future ... ?

Chronic patients												
	2019 / yes	2020 / yes	2021 / yes	2022 / yes	2023 / yes	2024 / yes	2019 / no	2020 / no	2021 / no	2022 / no	2023 / no	2024 / no
... an error caused by a medical treatment device												
Basis (100%)	366	356	320	342	339	314	634	643	676	658	660	686
yes, definitely	18%	19%	15%	17%	14%	18%	18%	21%	19%	19%	24%	23%
yes, probably	35%	38%	44%	42%	43%	37%	45%	41%	48%	46%	41%	44%
probably not	37%	32%	30%	31%	36%	34%	28%	29%	27%	28%	30%	27%
definitely not	8%	9%	8%	10%	8%	9%	7%	7%	5%	7%	4%	6%
wn/kA	2%	2%	3%			2%	2%	2%	1%		2%	
Total	100%	100%	100%	100%	100%	100%	100%	100%	100%	100%	100%	100%
yes, definitely/probably	52%	57%	59%	58%	56%	55%	63%	62%	67%	65%	64%	67%
definitely not/probably not	46%	41%	38%	41%	44%	43%	35%	36%	32%	35%	34%	32%

Question 6 and question 7: Not applicable

Question 8: We have now talked a little about the topic of 'patient safety'. How well informed do you feel about patient safety in general: 'very well' - 'well' - 'less well' - or 'not informed at all'?
'well' - 'less well' - or 'not informed at all'?

Basis (100%)	366	356	320	342	339	314	634	643	676	658	660	686
Very good	11%	15%	20%	16%	14%	14%	8%	8%	17%	13%	10%	11%
good	53%	55%	58%	48%	55%	56%	41%	58%	48%	51%	49%	47%
less good	28%	23%	14%	25%	26%	26%	38%	25%	27%	27%	32%	32%
Not informed at all	8%	7%	9%	10%	5%	4%	12%	8%	7%	8%	8%	10%
wn/kA		-	-			1%			1%			
sum	100%	100%	100%	100%	100%	100%	100%	100%	100%	100%	100%	100%
(very) good	64%	70%	78%	64%	69%	70%	50%	66%	65%	64%	60%	57%
less good/not at all	36%	30%	22%	35%	31%	30%	50%	34%	34%	35%	40%	42%

Question 9: All in all, what do you think: As a patient, can you yourself contribute to ensuring that you receive safe care at the doctor's surgery or hospital?
'yes, definitely' - 'rather yes' - 'rather not' - or
can you as a patient 'not at all' contribute to this?

Basis (100%)	366	356	320	342	339	314	634	643	676	658	660	686
yes, definitely	32%	42%	46%	39%	33%	33%	23%	30%	29%	25%	27%	26%
rather yes	42%	37%	32%	35%	42%	44%	43%	43%	44%	48%	41%	37%
rather not	19%	15%	16%	23%	17%	18%	25%	22%	19%	21%	27%	28%
not at all	6%	5%	7%	3%	8%	5%	6%	5%	8%	5%	4%	7%
wn/kA	2%				-		2%			1%		1%

						Chronic patients						
f24.0125 Sh/Kr/Ul, May 2024	2019 / yes	2020 / yes	2021 / yes	2022 / yes	2023 / yes	2024 / yes	2019 / no	2020 / no	2021 / no	2022 / no	2023 / no	2024 / no
definitely / rather yes	74%	79%	77%	73%	75%	77%	67%	73%	72%	73%	69%	64%
rather not/not at all	25%	21%	22%	26%	25%	23%	31%	27%	27%	26%	31%	35%

f24.0125 Sh/Kr/Ul, May 2024

Question 1: Not applicable

Question 2: 'Patient safety' refers to the successful endeavours to ensure error-free and harm-free medical treatment and medical healthcare. In your opinion, how likely is it that patients in Germany will come to harm as a result of medical treatment in hospital? Do you think this is 'very likely' - 'fairly likely' - 'not very likely' - or 'unlikely'?

	Regular use of prescription medication (according to S3C)											
	2019 / no	2020 / no	2021 / no	2022 / no	2023 / no	2024 / no	2019 / yes (in total)	2020 / yes (in total)	2021 / yes (in total)	2022 / yes (in total)	2023 / yes (in total)	2024 / yes (in total)
Basis (100%)	458	492	520	453	473	481	542	508	480	548	527	519
very likely	9%	5%	6%	12%	7%	8%	11%	12%	10%	10%	12%	10%
Fairly likely	32%	23%	17%	19%	19%	22%	38%	24%	21%	21%	22%	22%
not very likely	49%	58%	58%	56%	60%	53%	44%	56%	51%	60%	55%	58%
unlikely	9%	13%	19%	14%	13%	17%	6%	7%	17%	9%	10%	10%
wn/kA	2%	1%	-				1%	1%			1%	
Sum	100%	100%	100%	100%	100%	100%	100%	100%	100%	100%	100%	100%
very/somewhat likely	41%	28%	23%	30%	26%	30%	49%	36%	31%	31%	35%	32%
not very likely/unlikely	58%	71%	77%	70%	73%	70%	50%	63%	68%	69%	65%	68%

Question 3: And in your opinion, how likely is it that patients are harmed by medical care outside a hospital in

Germany, e.g. through outpatient treatment by a doctor or incorrect medication? Do you think this is 'very likely' - 'fairly likely' - 'not very likely'?
'fairly likely' - 'not very likely' - or
'unlikely'?

Basis (100%)	458	492	520	453	473	481	542	508	480	548	527	519
very likely	8%	4%	8%	9%	5%	5%	10%	10%	13%	5%	8%	7%
Fairly likely	28%	21%	25%	22%	22%	25%	31%	27%	18%	28%	27%	23%
not very likely	56%	63%	55%	59%	62%	58%	51%	54%	59%	62%	56%	60%
unlikely	8%	11%	12%	10%	10%	10%	7%	9%	8%	5%	8%	9%
wn/kA	-	1%		-	1%	1%			2%	1%	1%	1%
total	100%	100%	100%	100%	100%	100%	100%	100%	100%	100%	100%	100%
very/somewhat likely	36%	25%	33%	31%	27%	31%	42%	37%	31%	33%	35%	30%
not very likely/unlikely	64%	74%	67%	69%	72%	68%	58%	63%	67%	66%	63%	69%

Question 4: I will now tell you about possible harmful occurrences in medical care, which are also called
called 'adverse events'.

f24.0125 Sh/Kr/Ul, May 2024

Please tell me in each case whether you think this will happen to you: 'yes, definitely' - 'yes, probably' - 'probably not' - or 'definitely not'? How likely is it that the following will happen to you: ...?

Overview table: yes, definitely/probably

	Regular use of prescription drugs (according to S3C)											
	2019 / no	2020 / no	2021 / no	2022 / no	2023 / no	2024 / no	2019 / yes (in total)	2020 / yes (in total)	2021 / yes (in total)	2022 / yes (in total)	2023 / yes (in total)	2024 / yes (in total)
Basis (100%)	458	492	520	453	473	481	542	508	480	548	527	519
Infection with dangerous germs in hospital	53%	53%	57%	54%	50%	56%	71%	59%	67%	65%	63%	64%
an incorrect diagnosis	60%	48%	53%	51%	59%	55%	59%	53%	53%	50%	62%	52%
an error during an operation, e.g. a surgical error surgical error	35%	28%	31%	24%	25%	26%	47%	34%	38%	26%	32%	32%
an error with medicines due to incorrect prescription, incorrect dosage or incorrect method of use	49%	37%	41%	33%	39%	34%	48%	39%	47%	33%	46%	36%
an error caused by a medical treatment device	27%	17%	23%	18%	16%	16%	33%	27%	26%	16%	28%	19%

Question 4: I will now tell you about possible harmful events in medical care, which are also called called 'adverse events'.

Please tell me in each case whether you think this will happen to you: 'yes, definitely' - 'yes, probably' - 'probably not' - or 'definitely not'? How likely is it that the following will happen to you: ...?

... an infection with dangerous germs in hospital

Basis (100%)	458	492	520	453	473	481	542	508	480	548	527	519
yes, definitely (incl. has already happened to me)	10%	10%	13%	11%	11%	14%	22%	13%	17%	9%	12%	14%
yes, probably	44%	42%	44%	44%	39%	42%	49%	46%	50%	56%	50%	50%
probably not	37%	39%	37%	39%	43%	38%	25%	31%	28%	32%	33%	32%
definitely not	9%	6%	5%	6%	7%	6%	3%	9%	1%	3%	4%	4%
wn/kA	1%	2%		-	-	-			3%	1%	1%	
total	100%	100%	100%	100%	100%	100%	100%	100%	100%	100%	100%	100%
yes, definitely/probably	53%	53%	57%	54%	50%	56%	71%	59%	67%	65%	63%	64%
definitely not/probably not	46%	45%	43%	46%	50%	44%	29%	40%	30%	34%	37%	36%

Question 4: I will now tell you about possible harmful events in medical care, which are also called
called 'adverse events'.

f24.0125 Sh/Kr/Ul, May 2024

	Regular use of prescription drugs (according to S3C)											
	2019 / no	2020 / no	2021 / no	2022 / no	2023 / no	2024 / no	2019 / yes (in total)	2020 / yes (in total)	2021 / yes (in total)	2022 / yes (in total)	2023 / yes (in total)	2024 / yes (in total)
Please tell me in each case whether you think this will happen to you: 'yes, definitely' - 'yes, probably' - 'probably not' - or 'definitely not'? How likely is it that the following will happen to you: ...?												
... an incorrect diagnosis												
Basis (100%)	458	492	520	453	473	481	542	508	480	548	527	519
yes, definitely (incl. has already happened to me)	13%	12%	13%	11%	12%	13%	16%	17%	13%	11%	22%	13%
yes, probably	47%	36%	40%	40%	47%	42%	43%	36%	40%	38%	40%	40%
probably not	35%	45%	43%	44%	35%	37%	37%	40%	39%	46%	32%	38%
definitely not	5%	5%	4%	6%	5%	7%	4%	7%	7%	4%	6%	8%
wn/kA		1%				1%				-		1%
sum	100%	100%	100%	100%	100%	100%	100%	100%	100%	100%	100%	100%
yes, definitely/probably	60%	48%	53%	51%	59%	55%	59%	53%	53%	50%	62%	52%
definitely not/probably not	40%	51%	47%	49%	41%	44%	41%	47%	47%	50%	38%	47%

Question 4: I will now tell you about possible harmful events in medical care, which are also called
called 'adverse events'.

Please tell me in each case whether you think this will happen to you: 'yes, definitely' - 'yes, probably' - 'probably not' - or 'definitely not'? How likely is it that the following will happen to you: ...?

... an error during an operation, e.g. a surgical error

Basis (100%)	458	492	520	453	473	481	542	508	480	548	527	519
yes, definitely (incl. has already happened to me)	4%	5%	6%	3%	5%	5%	8%	9%	10%	7%	6%	4%
yes, probably	31%	23%	25%	20%	20%	21%	40%	26%	28%	19%	26%	28%
probably not	57%	57%	61%	68%	61%	63%	46%	53%	50%	63%	57%	61%
definitely not	8%	14%	8%	9%	14%	11%	6%	12%	10%	10%	11%	7%
wn/kA		1%	1%				1%	1%	2%			
Total	100%	100%	100%	100%	100%	100%	100%	100%	100%	100%	100%	100%
yes, definitely/probably	35%	28%	31%	24%	25%	26%	47%	34%	38%	26%	32%	32%
definitely not/probably not	65%	71%	69%	76%	75%	74%	52%	65%	60%	73%	68%	68%

f24.0125 Sh/Kr/Ul, May 2024

	Regular use of prescription drugs (according to S3C)											
	2019 / no	2020 / no	2021 / no	2022 / no	2023 / no	2024 / no	2019 / yes (in total)	2020 / yes (in total)	2021 / yes (in total)	2022 / yes (in total)	2023 / yes (in total)	2024 / yes (in total)
Please tell me in each case whether you think this will happen to you: 'yes, definitely' - 'yes, probably' - 'probably not' - or 'definitely not'? How likely is it that the following will happen to you: ...?												
... an error with medicines due to incorrect prescription, incorrect dosage or incorrect method of use												
Basis (100%)	458	492	520	453	473	481	542	508	480	548	527	519
yes, definitely (incl. has already happened to me)	6%	7%	9%	8%	6%	7%	10%	8%	15%	7%	13%	6%
yes, probably	43%	30%	32%	26%	33%	27%	38%	30%	32%	26%	33%	30%
probably not	42%	52%	50%	53%	49%	56%	43%	45%	40%	55%	43%	49%
definitely not	9%	11%	8%	14%	11%	10%	8%	16%	13%	12%	9%	14%
wn/kA		1%			1%		-			-	2%	
total	100%	100%	100%	100%	100%	100%	100%	100%	100%	100%	100%	100%
yes, definitely/probably	49%	37%	41%	33%	39%	34%	48%	39%	47%	33%	46%	36%
definitely not/probably not	51%	62%	59%	67%	61%	66%	52%	61%	53%	67%	52%	64%

Question 4: I will now tell you about possible harmful events in medical care, which are also called
called 'adverse events'.

Please tell me in each case whether you think this will happen to you: 'yes, definitely' - 'yes, probably' - 'probably not' - or 'definitely not'? How likely is it that the following will happen to you: ...?

... an error caused by a medical treatment device

Basis (100%)	458	492	520	453	473	481	542	508	480	548	527	519
yes, definitely (incl. has already happened to me)	5%	4%	3%	1%	2%	2%	4%	2%	4%	1%	4%	3%
yes, probably	22%	14%	20%	17%	13%	14%	29%	25%	22%	14%	24%	16%
probably not	60%	65%	66%	61%	68%	70%	56%	59%	58%	69%	59%	63%
definitely not	13%	15%	11%	20%	15%	14%	11%	13%	12%	15%	13%	17%
wn/kA		2%	1%		1%				3%		1%	1%
Sum	100%	100%	100%	100%	100%	100%	100%	100%	100%	100%	100%	100%

Question 5: I will now read these 'adverse events' to you again. Please tell me in each case whether you think this will be minimised in the future by

TK Patient Safety Monitor: time comparison 2019 to 2024

f24.0125 Sh/Kr/Ul, May 2024

'suitable measures' can be largely avoided: 'yes, definitely' - 'yes, probably' - 'probably not' - or 'definitely not'? Can the following be largely avoided in the future ... ?

Overview table: yes, definitely/probably

	Regular use of prescription drugs (according to S3C)											
	2019 / no	2020 / no	2021 / no	2022 / no	2023 / no	2024 / no	2019 / yes (in total)	2020 / yes (in total)	2021 / yes (in total)	2022 / yes (in total)	2023 / yes (in total)	2024 / yes (in total)
Basis (100%)	458	492	520	453	473	481	542	508	480	548	527	519
Infection with dangerous germs in hospital	61%	61%	64%	52%	61%	55%	66%	60%	70%	52%	63%	58%
an incorrect diagnosis	61%	59%	61%	54%	61%	60%	57%	58%	60%	51%	52%	58%
an error during an operation, e.g. a surgical error	53%	64%	59%	58%	64%	60%	57%	56%	57%	53%	57%	56%
an error with medicines due to incorrect prescription, wrong dosage or wrong type of application	67%	56%	67%	61%	62%	66%	58%	55%	61%	58%	57%	61%
an error caused by a medical treatment device	65%	66%	67%	66%	65%	69%	54%	55%	62%	60%	59%	58%

Question 5: I will now read these 'adverse events' to you again. Please tell me in each case whether you think that this can be largely avoided in future by

'suitable measures': 'yes, definitely' - 'yes, probably' - 'probably not' - or 'definitely not'? Can the following be largely avoided in future ... ?

... infection with dangerous germs in hospital

Basis (100%)	458	492	520	453	473	481	542	508	480	548	527	519
yes, definitely	17%	13%	15%	13%	19%	17%	24%	14%	12%	13%	15%	12%
yes, probably	44%	48%	50%	39%	42%	38%	42%	46%	58%	39%	48%	46%
probably not	31%	34%	30%	38%	34%	36%	27%	32%	24%	37%	31%	34%
definitely not	8%	5%	5%	9%	5%	9%	7%	8%	6%	10%	6%	8%
wn/kA		-	-	-	-	-		-	-	-	-	-
Total	100%	100%	100%	100%	100%	100%	100%	100%	100%	100%	100%	100%
yes, definitely/probably	61%	61%	64%	52%	61%	55%	66%	60%	70%	52%	63%	58%
definitely not/probably not	39%	39%	36%	48%	39%	45%	34%	40%	30%	48%	37%	42%

f24.0125 Sh/Kr/Ul, May 2024	Regular use of prescription drugs (according to S3C)											
	2019 / no	2020 / no	2021 / no	2022 / no	2023 / no	2024 / no	2019 / yes (in total)	2020 / yes (in total)	2021 / yes (in total)	2022 / yes (in total)	2023 / yes (in total)	2024 / yes (ins-total)
Basis (100%)	458	492	520	453	473	481	542	508	480	548	527	519
yes, definitely	18%	15%	16%	16%	20%	19%	16%	14%	16%	13%	11%	13%
yes, probably	43%	44%	45%	38%	42%	41%	41%	44%	45%	39%	41%	45%
probably not	33%	35%	33%	35%	33%	34%	35%	34%	33%	42%	42%	35%
definitely not	5%	5%	6%	11%	6%	6%	7%	8%	5%	6%	5%	7%
wn/kA	1%	1%	1%				1%		1%		1%	
total	100%	100%	100%	100%	100%	100%	100%	100%	100%	100%	100%	100%
yes, definitely/probably	61%	59%	61%	54%	61%	60%	57%	58%	60%	51%	52%	58%
definitely not/probably not	38%	40%	39%	45%	39%	40%	42%	42%	39%	49%	48%	42%

Question 5: I will now read these 'adverse events' to you again. Please tell me in each case whether you think that this can be largely avoided in future by
'suitable measures': 'yes, definitely' - 'yes, probably' - 'probably not' - or 'definitely not'? Can the following be largely avoided in future ... ?

... an error during an operation, e.g. a surgical error

Basis (100%)	458	492	520	453	473	481	542	508	480	548	527	519
yes, definitely	15%	13%	19%	16%	20%	23%	18%	16%	10%	13%	12%	11%
yes, probably	38%	51%	40%	42%	45%	37%	39%	40%	46%	39%	45%	45%
probably not	39%	30%	33%	31%	30%	32%	35%	32%	34%	41%	38%	36%
definitely not	8%	5%	7%	11%	5%	8%	7%	11%	9%	5%	6%	7%
wn/kA	1%	2%					1%	1%	1%	1%		1%
Sum	100%	100%	100%	100%	100%	100%	100%	100%	100%	100%	100%	100%
yes, definitely/probably	53%	64%	59%	58%	64%	60%	57%	56%	57%	53%	57%	56%
definitely not/probably not	47%	35%	41%	42%	35%	40%	42%	43%	42%	46%	43%	43%

Question 5: I will now read these 'adverse events' to you again. Please tell me in each case whether you think that this can be largely avoided in future by

... an error with medicines due to incorrect prescription, incorrect dosage or incorrect method of use

	Regular use of prescription medication (according to S3C)											
	2019 / no	2020 / no	2021 / no	2022 / no	2023 / no	2024 / no	2019 / yes (in total)	2020 / yes (in total)	2021 / yes (in total)	2022 / yes (in total)	2023 / yes (in total)	2024 / yes (in total)
Basis (100%)	458	492	520	453	473	481	542	508	480	548	527	519
yes, definitely	21%	20%	18%	21%	23%	23%	20%	18%	19%	17%	14%	20%
yes, probably	46%	36%	48%	39%	39%	43%	38%	37%	42%	41%	43%	41%
probably not	27%	37%	27%	29%	34%	29%	36%	34%	30%	35%	38%	31%
definitely not	6%	6%	6%	10%	4%	5%	5%	10%	7%	7%	4%	6%
wn/kA	1%	1%		-			1%	1%	2%	-		1%
total	100%	100%	100%	100%	100%	100%	100%	100%	100%	100%	100%	100%
yes, definitely/probably	67%	56%	67%	61%	62%	66%	58%	55%	61%	58%	57%	61%
definitely not/probably not	32%	43%	33%	39%	38%	34%	41%	44%	37%	42%	42%	38%

TK Patient Safety Monitor: time comparison 2019 to 2024

f24.0125 Sh/Kr/Ul, May 2024

Question 5: I will now read these 'adverse events' to you again. Please tell me in each case whether you think that this can be largely avoided in future by 'suitable measures': 'yes, definitely' - 'yes, probably' - 'probably not' - or 'definitely not'? Can the following be largely avoided in future ... ?

... an error caused by a medical treatment device

Basis (100%)	458	492	520	453	473	481	542	508	480	548	527	519
yes, definitely	19%	22%	20%	20%	24%	27%	17%	19%	15%	16%	17%	17%
yes, probably	46%	44%	47%	46%	40%	42%	37%	36%	46%	43%	42%	42%
probably not	26%	25%	27%	24%	29%	26%	36%	35%	29%	34%	34%	32%
definitely not	8%	7%	6%	10%	5%	5%	7%	8%	6%	7%	6%	8%
wn/kA	1%	2%			1%		2%	2%	3%		1%	1%
Sum	100%	100%	100%	100%	100%	100%	100%	100%	100%	100%	100%	100%
yes, definitely/probably	65%	66%	67%	66%	65%	69%	54%	55%	62%	60%	59%	58%
definitely not/probably not	34%	33%	33%	34%	34%	31%	43%	43%	35%	40%	41%	40%

Question 6 and question 7: Not applicable

Question 8: We have now talked a little about the topic of 'patient safety'. How well informed do you feel about patient safety in general: 'very well' - 'well' - 'less well' - or 'not informed at all'?
'well' - 'less well' - or 'not informed at all'?

Basis (100%)	458	492	520	453	473	481	542	508	480	548	527	519

very good	8%	9%	18%	14%	11%	12%	10%	12%	17%	14%	13%	12%
good	39%	51%	44%	44%	49%	43%	51%	62%	59%	56%	53%	55%
less good	38%	31%	30%	30%	31%	32%	31%	18%	15%	24%	29%	28%
Not informed at all	14%	9%	7%	12%	9%	13%	8%	7%	8%	6%	5%	4%
wn/kA		-	-			1%			2%			
total	100%	100%	100%	100%	100%	100%	100%	100%	100%	100%	100%	100%
(very) good	48%	60%	62%	58%	60%	55%	61%	75%	76%	70%	66%	67%
less good/not at all	52%	40%	38%	42%	40%	45%	39%	25%	23%	30%	34%	33%

TK Patient Safety Monitor: Time comparison 2019 to 2024

f24.0125 Sh/Kr/Ul, May 2024	Regular use of prescription medication (according to S3C)											
	2019 / no	2020 / no	2021 / no	2022 / no	2023 / no	2024 / no	2019 / yes (in total)	2020 / yes (in total)	2021 / yes (in total)	2022 / yes (in total)	2023 / yes (in total)	2024 / yes (in total)
Question 9: All in all, what do you think: Can you yourself as a patient yourself as a patient to ensure that you receive safe care in the doctor's surgery or hospital? 'yes, definitely' - 'rather yes' - 'rather not' - or can you as a patient 'not at all' contribute to this?												
Basis (100%)	458	492	520	453	473	481	542	508	480	548	527	519
yes, definitely	21%	30%	28%	26%	25%	26%	31%	38%	41%	33%	33%	31%
rather yes	45%	40%	45%	44%	44%	40%	41%	42%	34%	43%	39%	39%
rather not	27%	25%	21%	22%	25%	28%	19%	15%	16%	22%	22%	23%
not at all	6%	5%	6%	7%	5%	5%	7%	4%	9%	2%	6%	7%
wn/kA	1%					1%	2%			1%	-	1%
total	100%	100%	100%	100%	100%	100%	100%	100%	100%	100%	100%	100%
definitely/rather yes	66%	70%	73%	71%	69%	66%	72%	80%	75%	75%	73%	69%
rather not/not at all	33%	30%	27%	29%	31%	33%	26%	20%	25%	24%	27%	30%

f24.0125 Sh/Kr/Ul, May 2024

Question 1: Not applicable

Question 2: 'Patient safety' is the successful endeavour to ensure error-free and harm-free medical treatment and medical healthcare. In your opinion, how likely is it that patients in Germany will come to harm as a result of medical treatment in hospital? Do you think this is 'very likely' - 'fairly likely' - 'not very likely' - or 'unlikely'?

	Information status 'Patient safety' (according to F8)											
	2019 / (very) good	2020 / (very) good	2021 / (very) good	2022 / (very) good	2023 / (very) good	2024 / (very) good	2019 / less/g ar not	2020 / less/g ar not	2021 / less/g ar not	2022 / less/g ar not	2023 / less/g ar not	2024 / less/g ar not
Basis (100%)	549	676	688	645	628	611	448	323	304	354	370	384
very likely	8%	8%	8%	7%	8%	7%	13%	10%	7%	18%	13%	12%
Fairly likely	32%	20%	17%	19%	19%	18%	39%	32%	25%	23%	25%	28%
not very likely	50%	62%	58%	62%	60%	59%	42%	46%	50%	49%	53%	49%
unlikely	8%	10%	17%	12%	13%	15%	6%	12%	19%	10%	9%	10%
wn/kA	2%	1%			1%				-		1%	1%
Total of	100%	100%	100%	100%	100%	100%	100%	100%	100%	100%	100%	100%
very/somewhat likely	40%	27%	25%	25%	27%	25%	52%	42%	32%	40%	38%	40%
not very likely/unlikely	59%	71%	74%	75%	73%	75%	48%	57%	68%	59%	62%	60%

Question 3: And in your opinion, how likely is it that patients are harmed by medical care outside a hospital in

Germany, e.g. through outpatient treatment by a doctor or incorrect medication? Do you think this is 'very likely' - 'fairly likely' - 'not very likely'?
'fairly likely' - 'not very likely' - or
'unlikely'?

Basis (100%)	549	676	688	645	628	611	448	323	304	354	370	384
very likely	8%	6%	10%	4%	7%	5%	11%	8%	14%	12%	6%	7%
Fairly likely	30%	19%	22%	24%	21%	19%	30%	36%	21%	28%	31%	33%
not very likely	54%	64%	57%	65%	60%	64%	52%	47%	57%	52%	56%	51%
unlikely	8%	10%	11%	6%	10%	11%	7%	9%	8%	9%	7%	7%
wn/kA		1%			2%	1%				1%	1%	2%
Total	100%	100%	100%	100%	100%	100%	100%	100%	100%	100%	100%	100%
very/somewhat likely	38%	25%	31%	28%	28%	24%	41%	44%	35%	39%	37%	41%
not very likely/unlikely	62%	74%	68%	72%	70%	75%	59%	55%	65%	60%	63%	58%

Question 4: I will now tell you about possible harmful events in medical care, which are also called
called 'adverse events'.

f24.0125 Sh/Kr/Ul, May 2024

Please tell me in each case whether you think this will happen to you: 'yes, definitely' - 'yes, probably' - 'probably not' - or 'definitely not'? How likely is it that the following will happen to you: ...?

	Information status 'Patient safety' (according to F8)											
	2019 / (very) good	2020 / (very) good	2021 / (very) good	2022 / (very) good	2023 / (very) good	2024 / (very) good	2019 / less/g ar not	2020 / less/g ar not	2021 / less/g ar not	2022 / less/g ar not	2023 / less/g ar not	2024 / less/g ar not
Overview table: yes, definitely/probably												
Basis (100%)	549	676	688	645	628	611	448	323	304	354	370	384
Infection with dangerous germs in hospital	61%	55%	60%	57%	54%	55%	65%	59%	67%	65%	61%	68%
an incorrect diagnosis	52%	44%	46%	41%	54%	48%	69%	64%	68%	67%	73%	63%
an error during an operation, e.g. a surgical error surgical error	43%	28%	34%	21%	26%	24%	41%	38%	37%	32%	33%	36%
an error with medicines due to incorrect prescription, incorrect dosage or incorrect method of use	44%	32%	38%	28%	38%	30%	54%	51%	57%	42%	51%	43%
an error caused by a medical treatment device	29%	20%	22%	13%	18%	15%	33%	26%	31%	24%	28%	22%

Question 4: I will now tell you about possible harmful occurrences in medical care, which are also called called 'adverse events'.

Please tell me in each case whether you think this will happen to you: 'yes, definitely' - 'yes, probably' - 'probably not' - or 'definitely not'? How likely is it that the following will happen to you: ...?

... an infection with dangerous germs in hospital												
Basis (100%)	549	676	688	645	628	611	448	323	304	354	370	384
yes, definitely (incl. has already happened to me)	11%	9%	13%	8%	10%	10%	22%	17%	19%	14%	15%	20%
yes, probably	50%	45%	47%	50%	44%	45%	43%	43%	48%	51%	46%	49%
probably not	33%	36%	36%	37%	41%	38%	28%	34%	27%	31%	31%	29%
definitely not	6%	8%	4%	4%	5%	6%	5%	6%	3%	4%	7%	3%
wn/kA		1%		1%			1%	1%	3%	-	1%	
total	100%	100%	100%	100%	100%	100%	100%	100%	100%	100%	100%	100%
yes, definitely/probably	61%	55%	60%	57%	54%	55%	65%	59%	67%	65%	61%	68%
definitely not/probably not	39%	44%	40%	42%	46%	45%	34%	39%	30%	35%	38%	31%

Question 4: I will now tell you about possible harmful events in medical care, which are also called
called 'adverse events'.

f24.0125 Sh/Kr/Ul, May 2024	Information status 'Patient safety' (according to F8)											
	2019 / (very) good	2020 / (very) good	2021 / (very) good	2022 / (very) good	2023 / (very) good	2024 / (very) good	2019 / less/g ar not	2020 / less/g ar not	2021 / less/g ar not	2022 / less/g ar not	2023 / less/g ar not	2024 / less/g ar not
Please tell me in each case whether you think this will happen to you: 'yes, definitely' - 'yes, probably' - 'probably not' - or 'definitely not'? How likely is it that the following will happen to you: ...?												
... an incorrect diagnosis												
Basis (100%)	549	676	688	645	628	611	448	323	304	354	370	384
yes, definitely (incl. has already happened to me)	12%	11%	10%	9%	13%	10%	17%	22%	21%	15%	24%	17%
yes, probably	39%	33%	36%	32%	40%	38%	52%	42%	47%	52%	49%	46%
probably not	42%	48%	47%	54%	39%	42%	28%	31%	30%	29%	24%	30%
definitely not	6%	7%	7%	5%	7%	9%	3%	5%	2%	4%	3%	6%
wn/kA		1%		-		1%		1%		-	-	1%
total	100%	100%	100%	100%	100%	100%	100%	100%	100%	100%	100%	100%
yes, definitely/probably	52%	44%	46%	41%	54%	48%	69%	64%	68%	67%	73%	63%
definitely not/probably not	48%	55%	54%	59%	46%	52%	31%	35%	32%	33%	27%	36%

Question 4: I will now tell you about possible harmful events in medical care, which are also called
called 'adverse events'.

Please tell me in each case whether you think this will happen to you: 'yes, definitely' - 'yes, probably' - 'probably not' - or 'definitely not'? How likely is it that the following will happen to you: ...?

... an error during an operation, e.g. a surgical error

Basis (100%)	549	676	688	645	628	611	448	323	304	354	370	384
yes, definitely (incl. has already happened to me)	4%	5%	7%	5%	4%	2%	9%	10%	11%	6%	8%	10%
yes, probably	39%	23%	27%	16%	22%	23%	32%	28%	26%	26%	25%	26%
probably not	49%	57%	54%	68%	62%	65%	53%	50%	57%	60%	55%	57%
definitely not	8%	14%	10%	11%	12%	11%	6%	11%	6%	8%	12%	6%
wn/kA		1%	2%					1%				1%
total	100%	100%	100%	100%	100%	100%	100%	100%	100%	100%	100%	100%
yes, definitely/probably	43%	28%	34%	21%	26%	24%	41%	38%	37%	32%	33%	36%
definitely not/probably not	57%	71%	65%	79%	74%	75%	59%	61%	63%	68%	67%	64%

f24.0125 Sh/Kr/Ul, May 2024

Please tell me in each case whether you think this will happen to you: 'yes, definitely' - 'yes, probably' - 'probably not' - or 'definitely not'? How likely is it that the following will happen to you: ...?

... an error with medicines due to incorrect prescription, incorrect dosage or incorrect method of use

	Information status 'Patient safety' (according to F8)											
	2019 / (very) good	2020 / (very) good	2021 / (very) good	2022 / (very) good	2023 / (very) good	2024 / (very) good	2019 / less/g ar not	2020 / less/g ar not	2021 / less/g ar not	2022 / less/g ar not	2023 / less/g ar not	2024 / less/g ar not
Basis (100%)	549	676	688	645	628	611	448	323	304	354	370	384
yes, definitely (incl. has already happened to me)	7%	7%	11%	5%	7%	5%	10%	10%	12%	12%	15%	9%
yes, probably	37%	25%	27%	23%	31%	25%	44%	41%	45%	31%	36%	34%
probably not	45%	54%	49%	57%	49%	54%	39%	36%	38%	49%	41%	51%
definitely not	10%	14%	13%	15%	12%	16%	7%	13%	5%	8%	8%	6%
wn/kA		1%		-	2%			-	-	-		
total	100%	100%	100%	100%	100%	100%	100%	100%	100%	100%	100%	100%
yes, definitely/probably	44%	32%	38%	28%	38%	30%	54%	51%	57%	42%	51%	43%
definitely not/probably not	56%	68%	62%	72%	60%	70%	46%	49%	43%	58%	49%	57%

Question 4: I will now tell you about possible harmful events in medical care, which are also called
called 'adverse events'.

Please tell me in each case whether you think this will happen to you: 'yes, definitely' - 'yes, probably' - 'probably not' - or 'definitely not'? How likely is it that the following will happen to you: ...?

... an error caused by a medical treatment device

Basis (100%)	549	676	688	645	628	611	448	323	304	354	370	384
yes, definitely (incl. has already happened to me)	5%	2%	3%	1%	3%	1%	5%	4%	4%	2%	5%	5%
yes, probably	24%	18%	18%	12%	16%	14%	28%	22%	27%	22%	24%	17%
probably not	59%	64%	65%	69%	66%	66%	57%	59%	56%	59%	59%	68%
definitely not	12%	15%	11%	18%	15%	20%	10%	13%	13%	17%	12%	9%
wn/kA		1%	2%		1%			2%		-	1%	1%
total	100%	100%	100%	100%	100%	100%	100%	100%	100%	100%	100%	100%

f24.0125 Sh/Kr/Ul, May 2024

'suitable measures' can be largely avoided: 'yes, definitely' - 'yes, probably' - 'probably not' - or 'definitely not'? Can the following be largely avoided in the future ... ?

Overview table: yes, definitely/probably

	Information status 'Patient safety' (according to F8)											
	2019 / (very) good	2020 / (very) good	2021 / (very) good	2022 / (very) good	2023 / (very) good	2024 / (very) good	2019 / less/g ar not	2020 / less/g ar not	2021 / less/g ar not	2022 / less/g ar not	2023 / less/g ar not	2024 / less/g ar not
Basis (100%)	549	676	688	645	628	611	448	323	304	354	370	384
Infection with dangerous germs in hospital	66%	61%	67%	54%	61%	58%	60%	58%	67%	50%	63%	54%
an incorrect diagnosis	61%	57%	61%	52%	58%	62%	56%	62%	58%	54%	54%	55%
an error during an operation, e.g. a surgical error surgical error	60%	59%	59%	52%	59%	59%	49%	60%	57%	60%	63%	57%
an error with medicines due to incorrect prescription, incorrect dosage or incorrect method of use	65%	55%	64%	56%	60%	67%	59%	57%	63%	65%	59%	58%
an error caused by a medical treatment device	58%	58%	62%	60%	62%	65%	62%	65%	71%	66%	60%	62%

Question 5: I will now read these 'adverse events' to you again. Please tell me in each case whether you think that this can be largely avoided in future by

'suitable measures': 'yes, definitely' - 'yes, probably' - 'probably not' - or 'definitely not'? Can the following be largely avoided in future ... ?

... infection with dangerous germs in hospital

	2019 / (very) good	2020 / (very) good	2021 / (very) good	2022 / (very) good	2023 / (very) good	2024 / (very) good	2019 / less/g ar not	2020 / less/g ar not	2021 / less/g ar not	2022 / less/g ar not	2023 / less/g ar not	2024 / less/g ar not
Basis (100%)	549	676	688	645	628	611	448	323	304	354	370	384
yes, definitely	23%	13%	12%	13%	14%	15%	19%	13%	16%	14%	20%	13%
yes, probably	44%	48%	54%	41%	47%	42%	42%	46%	51%	36%	43%	42%
probably not	28%	32%	28%	37%	34%	35%	30%	36%	26%	39%	30%	35%
definitely not	6%	7%	5%	9%	5%	7%	9%	5%	8%	11%	7%	10%
wn/kA		-	-	-	-	-		-	-	-	-	-
Total	100%	100%	100%	100%	100%	100%	100%	100%	100%	100%	100%	100%
yes, definitely/probably	66%	61%	67%	54%	61%	58%	60%	58%	67%	50%	63%	54%
definitely not/probably not	33%	39%	33%	46%	39%	42%	40%	42%	33%	50%	37%	46%

Question 5: I will now read these 'adverse events' to you again. Please tell me in each case whether you think that this can be largely avoided in future by

'suitable measures': 'yes, definitely' - 'yes, probably' - 'probably not' - or 'definitely not'? Can the following be largely avoided in future ... ?

... an incorrect diagnosis

TK Patient Safety Monitor: Time comparison 2019 to 2024

f24.0125 Sh/Kr/Ul, May 2024	Information status 'Patient safety' (according to F8)											
	2019 / (very) good	2020 / (very) good	2021 / (very) good	2022 / (very) good	2023 / (very) good	2024 / (very) good	2019 / less/g ar not	2020 / less/g ar not	2021 / less/g ar not	2022 / less/g ar not	2023 / less/g ar not	2024 / less/g ar not
Basis (100%)	549	676	688	645	628	611	448	323	304	354	370	384
yes, definitely	18%	15%	16%	13%	14%	18%	16%	14%	12%	16%	17%	12%
yes, probably	43%	42%	45%	39%	44%	44%	40%	48%	45%	38%	37%	43%
probably not	31%	35%	33%	41%	38%	32%	38%	32%	33%	36%	38%	38%
definitely not	7%	7%	5%	7%	4%	6%	6%	5%	7%	10%	8%	7%
wn/kA	1%	1%			1%		-	1%	1%			1%
total	100%	100%	100%	100%	100%	100%	100%	100%	100%	100%	100%	100%
yes, definitely/probably	61%	57%	61%	52%	58%	62%	56%	62%	58%	54%	54%	55%
definitely not/probably not	38%	42%	38%	48%	42%	38%	44%	37%	41%	46%	46%	45%

Question 5: I will now read these 'adverse events' to you again. Please tell me in each case whether you think that this can be largely avoided in future by

'suitable measures': 'yes, definitely' - 'yes, probably' - 'probably not' - or 'definitely not'? Can the following be largely avoided in future ... ?

... an error during an operation, e.g. a surgical error

Basis (100%)	549	676	688	645	628	611	448	323	304	354	370	384
yes, definitely	18%	16%	15%	13%	14%	16%	14%	10%	13%	18%	17%	17%
yes, probably	42%	43%	44%	39%	44%	43%	35%	50%	44%	42%	46%	39%
probably not	33%	31%	33%	38%	36%	34%	40%	32%	36%	32%	31%	32%
definitely not	6%	9%	8%	8%	5%	6%	9%	7%	7%	8%	6%	10%
wn/kA	1%	1%	1%	1%		1%	2%	1%	1%	-		
Total	100%	100%	100%	100%	100%	100%	100%	100%	100%	100%	100%	100%
yes, definitely/probably	60%	59%	59%	52%	59%	59%	49%	60%	57%	60%	63%	57%
definitely not/probably not	39%	39%	40%	47%	41%	40%	49%	39%	43%	40%	37%	43%

Question 5: I will now read these 'adverse events' to you again. Please tell me in each case whether you think that this can be largely avoided in future by

f24.0125 Sh/Kr/Ul, May 2024

'suitable measures': 'yes, definitely' - 'yes, probably' - 'probably not' - or 'definitely not'? Can the following be largely avoided in future ... ?

... an error with medicines due to incorrect prescription, incorrect dosage or incorrect method of use

	Information status 'Patient safety' (according to F8)											
	2019 / (very) good	2020 / (very) good	2021 / (very) good	2022 / (very) good	2023 / (very) good	2024 / (very) good	2019 / less/g ar not	2020 / less/g ar not	2021 / less/g ar not	2022 / less/g ar not	2023 / less/g ar not	2024 / less/g ar not
Basis (100%)	549	676	688	645	628	611	448	323	304	354	370	384
yes, definitely	21%	19%	21%	18%	18%	23%	20%	20%	15%	21%	19%	20%
yes, probably	45%	36%	43%	38%	42%	44%	38%	38%	48%	44%	40%	38%
probably not	30%	36%	29%	35%	36%	27%	34%	35%	28%	28%	37%	34%
definitely not	4%	8%	5%	9%	4%	4%	7%	7%	8%	7%	4%	8%
wn/kA		1%	1%	-		1%	1%	1%	1%	-		
Total	100%	100%	100%	100%	100%	100%	100%	100%	100%	100%	100%	100%
yes, definitely/probably	65%	55%	64%	56%	60%	67%	59%	57%	63%	65%	59%	58%
definitely not/probably not	34%	44%	35%	44%	40%	32%	40%	42%	36%	35%	41%	42%

Question 5: I will now read these 'adverse events' to you again. Please tell me in each case whether you think that this can be largely avoided in future by 'suitable measures': 'yes, definitely' - 'yes, probably' - 'probably not' - or 'definitely not'? Can the following be largely avoided in future ... ?

... an error caused by a medical treatment device

Basis (100%)	549	676	688	645	628	611	448	323	304	354	370	384
yes, definitely	17%	20%	19%	17%	22%	22%	20%	21%	16%	21%	18%	22%
yes, probably	41%	38%	43%	44%	40%	43%	42%	45%	55%	46%	42%	40%
probably not	33%	32%	29%	32%	31%	27%	29%	26%	24%	25%	34%	32%
definitely not	7%	8%	7%	8%	6%	7%	8%	7%	4%	8%	5%	6%
wn/kA	2%	2%	2%		1%	1%	1%	1%	1%			1%
total	100%	100%	100%	100%	100%	100%	100%	100%	100%	100%	100%	100%
yes, definitely/probably	58%	58%	62%	60%	62%	65%	62%	65%	71%	66%	60%	62%
definitely not/probably not	40%	40%	36%	39%	36%	34%	37%	33%	29%	33%	39%	38%

Question 6 and question 7: Not applicable

Question 8: We have now talked a little about the topic of 'patient safety'. How well informed do you feel about patient safety in general: 'very well' - 'well' - 'less well' - or 'not informed at all'?

'well' - 'less well' - or 'not informed at all'?

Basis (100%)	549	676	688	645	628	611	448	323	304	354	370	384

very good	17%	16%	26%	22%	19%	19%	-	-	-	-	-	-
good	83%	84%	74%	78%	81%	81%	-	-	-	-	-	-
less good	-	-	-	-	-	-	76%	75%	75%	75%	81%	78%
not informed at all	-	-	-	-	-	-	24%	25%	25%	25%	19%	22%
wn/kA	-	-	-	-	-	-	-	-	-	-	-	-
Total	100%	100%	100%	100%	100%	100%	100%	100%	100%	100%	100%	100%
(very) good	100%	100%	100%	100%	100%	100%	-	-	-	-	-	-
less good/not at all	-	-	-	-	-	-	100%	100%	100%	100%	100%	100%

TK Patient Safety Monitor: Time comparison 2019 to 2024

f24.0125 Sh/Kr/Ul, May 2024	Information status 'Patient safety' (according to F8)											
	2019 / (very) good	2020 / (very) good	2021 / (very) good	2022 / (very) good	2023 / (very) good	2024 / (very) good	2019 / less/g ar not	2020 / less/g ar not	2021 / less/g ar not	2022 / less/g ar not	2023 / less/g ar not	2024 / less/g ar not
Question 9: All in all, what do you think: As a patient, can you yourself yourself as a patient to ensure that you receive safe care at the doctor's surgery or hospital? 'yes, definitely' - 'rather yes' - 'rather not' - or can you as a patient 'not at all' contribute to this?												
Basis (100%)	549	676	688	645	628	611	448	323	304	354	370	384
yes, definitely	35%	40%	41%	36%	38%	37%	16%	23%	19%	17%	16%	15%
rather yes	44%	45%	41%	46%	44%	40%	42%	33%	39%	39%	37%	38%
rather not	17%	13%	14%	14%	15%	20%	30%	33%	28%	37%	38%	34%
not at all	4%	2%	4%	3%	3%	2%	9%	11%	14%	7%	9%	13%
wn/kA	1%			1%		1%	2%			-		
Total	100%	100%	100%	100%	100%	100%	100%	100%	100%	100%	100%	100%
definitely/rather yes	78%	84%	82%	83%	82%	77%	59%	56%	57%	56%	52%	53%
rather not/not at all	21%	15%	18%	17%	18%	22%	39%	44%	42%	44%	48%	47%
